# Prolyl-isomerase Pin1 controls normal and cancer stem cells of the breast

**DOI:** 10.1002/emmm.201302909

**Published:** 2013-12-16

**Authors:** Alessandra Rustighi, Alessandro Zannini, Luca Tiberi, Roberta Sommaggio, Silvano Piazza, Giovanni Sorrentino, Simona Nuzzo, Antonella Tuscano, Vincenzo Eterno, Federica Benvenuti, Libero Santarpia, Iannis Aifantis, Antonio Rosato, Silvio Bicciato, Alberto Zambelli, Giannino Del Sal

**Affiliations:** 1 Laboratorio Nazionale CIB (LNCIB), Area Science ParkTrieste, Italy; 2 Dipartimento di Scienze della Vita, Università degli Studi di TriesteTrieste, Italy; 3 Dipartimento di Scienze Oncologiche e Chirurgiche, Università degli Studi di Padova e Istituto Oncologico Veneto IRCCSPadova, Italy; 4 Center for Genome Research, Dipartimento di Scienze della Vita, Università degli Studi di Modena e Reggio EmiliaModena, Italy; 5 Oncology Department IRCCS Fondazione Salvatore MaugeriPavia, Italy; 6 International Centre for Genetic Engineering and Biotechnology (ICGEB), Area Science ParkTrieste, Italy; 7 Translational Research Unit, Istituto Toscano TumoriPrato, Italy; 8 Howard Hughes Medical Institute and Department of Pathology, NYU School of MedicineNew York, NY, USA

**Keywords:** breast cancer, Fbxw7 E3 ubiquitin-ligase, Notch, prolyl-isomerase Pin1, stem cells

## Abstract

Mammary epithelial stem cells are fundamental to maintain tissue integrity. Cancer stem cells (CSCs) are implicated in both treatment resistance and disease relapse, and the molecular bases of their malignant properties are still poorly understood. Here we show that both normal stem cells and CSCs of the breast are controlled by the prolyl-isomerase Pin1. Mechanistically, following interaction with Pin1, Notch1 and Notch4, key regulators of cell fate, escape from proteasomal degradation by their major ubiquitin-ligase Fbxw7α. Functionally, we show that Fbxw7α acts as an essential negative regulator of breast CSCs' expansion by restraining Notch activity, but the establishment of a Notch/Pin1 active circuitry opposes this effect, thus promoting breast CSCs self-renewal, tumor growth and metastasis *in vivo*. In human breast cancers, despite Fbxw7α expression, high levels of Pin1 sustain Notch signaling, which correlates with poor prognosis. Suppression of Pin1 holds promise in reverting aggressive phenotypes, through CSC exhaustion as well as recovered drug sensitivity carrying relevant implications for therapy of breast cancers.

## Introduction

Breast cancer is the most frequently diagnosed cancer and the leading cause of cancer mortality in females worldwide (Siegel *et al*, 2011). Despite advances in diagnosis and treatment, a significant percentage of breast cancer patients still die, due to the development and dissemination of metastases (Steeg & Theodorescu, 2008). It is increasingly acknowledged that a subpopulation of cancer cells, termed cancer stem cells (CSCs) play a major role in cancer growth, metastasis formation and chemoresistance (Dean *et al*, 2005; Stingl & Caldas, 2007; Visvader & Lindeman, 2012). Like their normal counterpart, CSCs are able to self-renew and maintain a reservoire of cancer-initiating cells, that may produce a more differentiated progeny of cells and contribute to intratumor heterogeneity (Stingl & Caldas, 2007). This evidence has been observed for breast cancers, where it has been shown that poorly differentiated, more aggressive tumors (histological grade 3) have an increased number of CSCs than well differentiated (histological grade 1) tumors (Pece *et al*, 2010).

Considerable similarities are found between normal and CSCs regarding the molecular pathways and stem cell factors that determine the undifferentiated state of these cells, which suggested that CSCs originate from the transformation of adult tissue stem cells or from more differentiated progenitors that have acquired self-renewal ability (Reya *et al*, 2001; Ben-Porath *et al*, 2008; Visvader & Lindeman, 2012). Several studies indicated that oncogenic activation of pathways involved in the regulation of normal stem cells, such as Notch, Wnt, SHH, RTKs, and PI3K/AKT among others, might be involved in self-renewal properties and aggressive features of CSCs (Polyak & Weinberg, 2009; Thiery *et al*, 2009; Visvader & Lindeman, 2012). However, how these signaling networks govern CSCs still remains to be elucidated.

One appealing candidate as a fine-tuner of stem cell traits might be the prolyl-isomerase Pin1. This unique enzyme catalyzes the *cis/trans* conversion of specific motifs composed by phosphorylated Serines or Threonines preceding a Proline in certain proteins, thereby inducing conformational changes required for the full activity and cross-talk of a plethora of signaling pathways (Liou *et al*, 2011). Specifically, Serine/Threonine-Proline motifs (Ser/Thr-Pro) are exclusive phosphorylation sites for a series of proline-directed kinases, such as glycogen synthase kinase 3 beta, cyclin-dependent and MAP kinases, that fulfill key roles in the control of signal transduction.

The discovery of Pin1-catalyzed *cis/trans* isomerization of phospho-Ser/Thr-Pro motifs revealed a post-phosphorylation mechanism critical for several biological processes involved in physiology and disease (Lu & Zhou, 2007; Yeh & Means, 2007). In particular, Pin1 is required for full activity and cross-talk of a variety of oncogenic pathways in breast and other cancers (Wulf *et al*, 2005), acting as an amplifier of phosphorylation signals. Of note, deregulated levels of Pin1 have been shown to disrupt cellular polarity of breast epithelial cells (Ryo *et al*, 2002) and found associated to high tumor grade and aggressiveness in breast cancer (Wulf *et al*, 2001; Girardini *et al*, 2011). However, so far Pin1-dependent signaling mechanisms have not been linked to breast CSCs' biology.

In this work, by performing *in vivo* and *in vitro* functional studies in mouse models and cell lines, we show that Pin1 acts as a fundamental regulator of stem cell features both in normal stem cells and CSCs of the mammary gland. Pin1 controls CSC self-renewal, replicative potential and frequency by antagonizing the negative effect of Fbxw7α E3 ubiquitin-ligase on the Notch receptor pathway, a fundamental regulator of cell fate frequently subverted in breast cancer (Han *et al*, 2011; Ranganathan *et al*, 2011; Reedijk, 2012). At the biochemical level, we demonstrate that Notch1 and Notch4 escape from Fbxw7α-dependent proteasomal degradation following interaction with Pin1 and that phospho-specific prolyl-isomerization of Notch1 triggers de-phosphorylation by the PP2A phosphatase, preventing Fbxw7α interaction and subsequent poly-ubiquitination. While mouse xenograft experiments prove the relevance of Pin1 in tumor growth and metastasis formation *in vivo,* gene expression and immunohistochemical analyses of primary tumors from breast cancer patients show that Pin1 overexpression is significantly linked to activated Notch, irrespectively of the coexistance of functional Fbxw7α. Clinical implications of our findings are relevant for breast cancer, since inhibition of Pin1 could suppress aggressive phenotypes through CSC exhaustion as well as recovered sensitivity to chemotherapeutic drugs.

## Results

### The prolyl-isomerase Pin1 is required for the self-renewal of normal mammary stem cells

**Figure 1:**
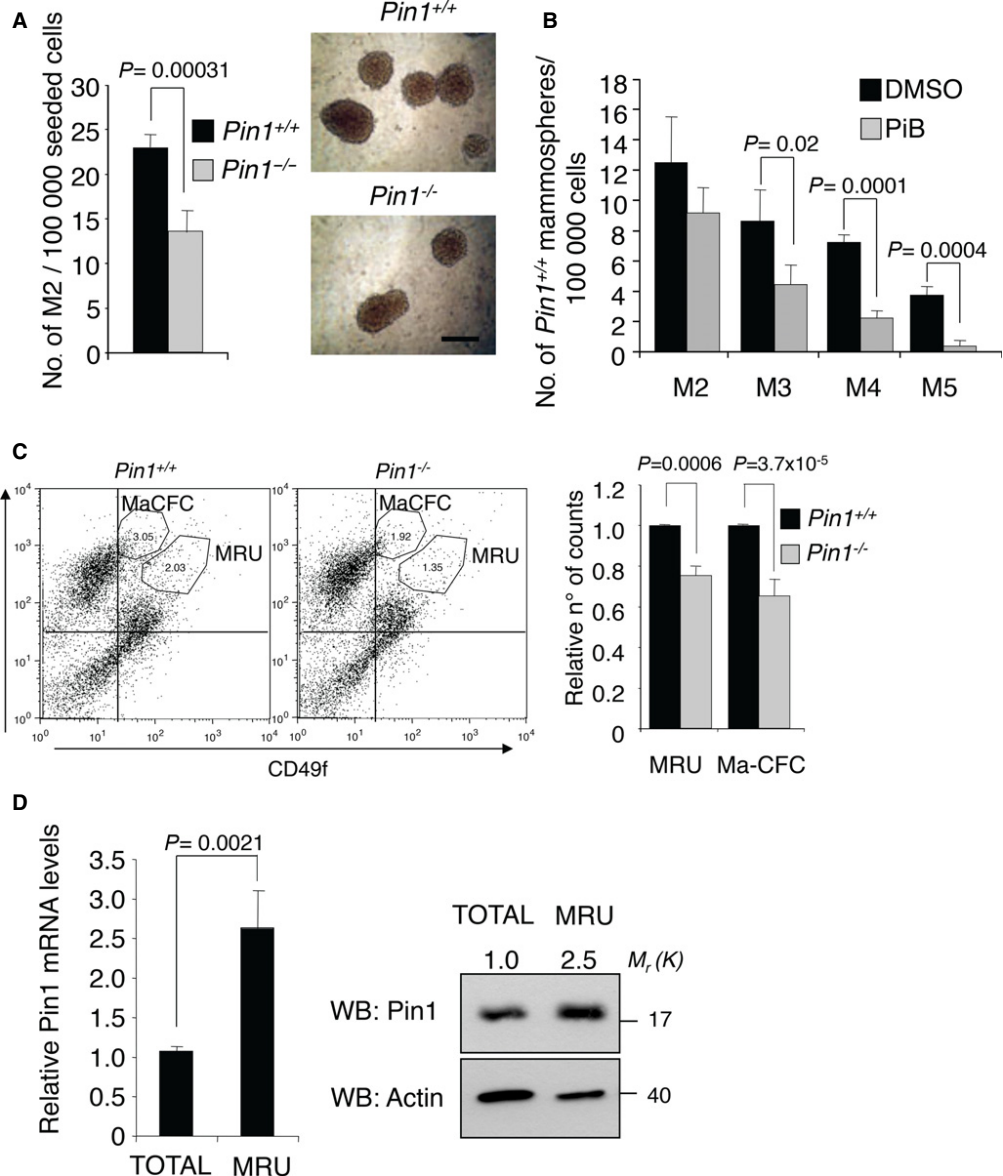
Pin1^*−/−*^ mice have decreased mammary epithelial stem/progenitor cells. A  *Pin1*^*−/−*^ mice display decreased self-renewal of mammary stem cells. Left panel: Number of secondary mammospheres (M2) generated from primary mammary epithelial cells of indicated mice. Means, standard deviations and *P*-values (*t*-test, *n* = 4) are indicated in the histogram. Right panel: representative M2 microscope images with 200 μm scale bar. B  Inhibition of Pin1 affects replicative potential of mammary stem cells. Serial replating of mammospheres (M1–M5) generated from *Pin*^*+/+*^ mice treated with DMSO or PiB (1.5 μM). C  Bipotent stem cell and luminal progenitor number is decreased in *Pin1*^*−/−*^ mammary tissue. Left panel: representative FACS analyses of mammary epithelial cells from indicated mice. CD24/CD49f plots and gatings for MRU and Ma-CFC populations are indicated. Right panel: histogram of mean counts of MRU and MA-CFC populations from *Pin*^*−/−*^ normalized to *Pin1*^*+/+*^ mice. Means, standard deviations and *P*-values (*t*-test, *n* = 3) are indicated. D  Pin1 mRNA and protein levels are upregulated in the mammary stem cell compartment. Left panel: qRT-PCR of endogenous Pin1 mRNA in MRU sorted populations relative to total population. Means, standard deviations and *P*-values (*t*-test, *n* = 3) are indicated. Right panel: Western blot analysis of the same cell populations as in the left panel. Fold change in Pin1 protein levels determined by Image J software (Rasband, 1997–2012) with respect to actin levels is indicated by a number, Molecular weights in kDa (*M*_r_ (K)) are shown on the right.

Pin1 knock-out mice show a number of developmental defects (Atchison & Means, 2004) affecting among others mammary epithelium, that fails to undergo the dynamic changes required to its expansion during pregnancy (Liou *et al*, 2002). Based on this, we hypothesized a possible function of Pin1 in governing the functions of mammary stem cells and thus we evaluated the stem cell activity of mammary epithelial cells from wild-type (*Pin1*^*+/+*^) and knock-out (*Pin1*^*−/−*^) mice. To this aim, mammary tissues from 8 to 10 weeks old virgin female mice were dissociated, prepared as single cell suspensions of purified, lineage-depleted epithelial cells (Sleeman *et al*, 2006; Stingl *et al*, 2006) and grown in suspension cultures to form secondary mammospheres (M2) (Dontu *et al*, 2003). Whereas cells obtained from *Pin1*^*+/+*^ mice formed an average of 22.9 (±1.44) M2 mammospheres per 100 000 seeded cells, we observed a 40% reduction of M2 formation from *Pin1*^*−/−*^ cells (Fig 1A). In addition, to assess the impact of Pin1 on the replicative potential of mammary stem cells, we serially replated wild-type cells from primary mammospheres (M1) for four more times (M2–M5) (Fig 1B). As expected in these conditions, we observed a progressive decrease in mammosphere formation at each passage, due to exhaustion of adult stem cells (Cicalese *et al*, 2009). Notably, this effect was significantly exacerbated by addition of the Pin1 small molecule inhibitor PiB (Uchida *et al*, 2003): mammosphere formation efficiency of *Pin1*^*+/+*^ shrunk progressively and was reduced by almost 50% at the stadium of quaternary mammospheres (M4) and did not reach the M5 level. This evidence indicates a role for Pin1 in determining self-renewal and replicative potential of mammary stem cells thus implying alterations of the mammary stem cell compartment in *Pin1*^*−/−*^ mice. To better characterize this aspect, we analyzed the proportion of stem cells and progenitors by Flow cytometric analyses and sorting (FACS) analysis using the surface markers CD24 and CD49f. These markers are widely used to identify two populations of cells functionally characterized as stem/bipotent progenitors (CD24^med^/CD49f^high^ or mammary repopulating units, MRU) and luminal progenitors (CD24^high^/CD49f^low^ or mammary colony forming cells, Ma-CFCs) (Stingl *et al*, 2006). In line with our hypothesis, the MRU and Ma-CFC cell populations from *Pin1*^*−/−*^ mammary glands were present at lower proportion as compared to *Pin1*^*+/+*^ mice (Fig 1C and supplementary Fig S1A). In addition, we found almost three times higher Pin1 mRNA and protein levels in the MRU cell population as compared to the total of mammary epithelial cells (Fig 1D). This evidence confirmed our hypothesis and suggests a prominent role of Pin1 in sustaining the mammary stem cell compartment *in vivo*.

### Pin1 is required to sustain CSCs from mouse and human mammary tumor cells

**Figure 2:**
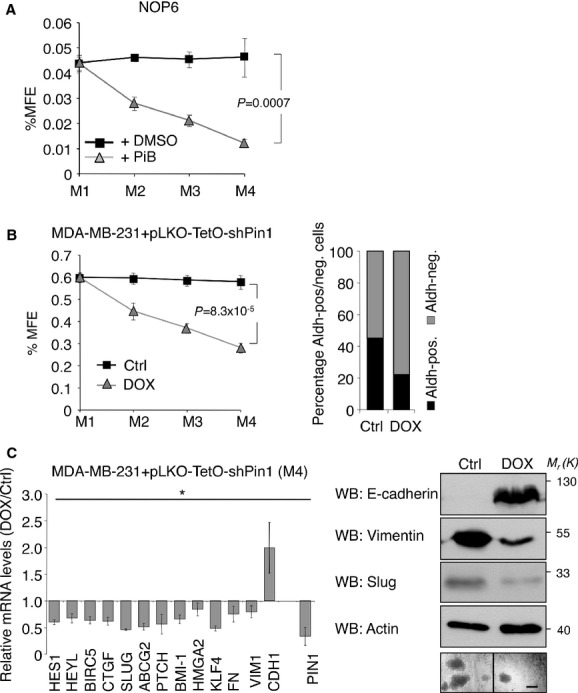
Pin1 inhibition strongly affects mouse and human mammary CSCs traits. (A) and (B) Means and standard deviations and *P*-values are indicated (*t*-test, *n* = 3, M4). A  Pin1 inhibition decreases self-renewal of mouse mammary tumor cells. Serial replating of mammospheres (M1–M4) generated from NOP6 cells treated with DMSO or PiB (1.5 μM). Mammosphere formation efficiency (%MFE) was calculated as percentage of mammospheres divided by the number of plated cells. B  Pin1 knockdown decreases self-renewal of human breast cancer cells. Left panel: MFE of MDA-MB-231-pLKO-shPin1 control cells (Ctrl) compared to shPin1 induced cells (DOX) upon serial passages. Right panel: Quantification of Aldh-positive and Aldh-negative cells from control- and shPin1 induced M4, as assessed by FACS. C  Pin1 knockdown affects expression of stem cell markers. Left panel: qRT-PCR of the indicated stemness and EMT marker genes from MDA-MB-231-pLKO-shPin1 quaternary mammospheres (M4) upon shPin1 induction (DOX) with respect to control cells (Ctrl). Standard deviations are indicated, *P*-values * <0.02 (*t*-test, *n* = 3). Right panel: Western Blot analysis of EMT markers of the same cells. Molecular weights *M*_r_(K) are indicated in kDa. Representative microphotographs of M4 are shown, 200 μm scale bar is indicated.

Stem cell traits in a subpopulation of mammary tumor cells are thought to be implicated in treatment resistance (Dean *et al*, 2005) and metastasis dissemination (Malanchi *et al*, 2012; Rosenthal *et al*, 2012; Visvader & Lindeman, 2012) and high levels of Pin1 correlate with high grade breast cancer and chemoresistance (Wulf *et al*, 2001; Ding *et al*, 2008; Kim *et al*, 2009; Girardini *et al*, 2011). Therefore we next chose to investigate whether Pin1 could also control mammary CSCs. NOP6 mouse mammary tumor cells, harboring the Her2/Neu amplification, were grown as mammospheres in presence or absence of the Pin1 inhibitor (Fig 2A). NOP6 cells formed very fast growing spheres that did not decrease when propagated to M3 or M4, indicating that mammosphere-forming cells were self-renewing at a constant rate. Conversely, when cells were treated with Pin1 inhibitor, mammosphere formation efficiency (MFE) was strongly impaired already at the M2 level.

We next tested whether Pin1 could be required for the maintenance of human breast CSCs. To address this question, the MDA-MB-231 breast cancer cell line expressing a doxycycline-inducible knockdown costruct for Pin1 (pLKO-TetO-shPin1) was generated and tested in mammosphere formation assays. As shown in Fig 2B (left), in agreement with other reports (Harrison *et al*, 2010; Cordenonsi *et al*, 2011), non-induced cells had on average 0.6% of MFE, remaining constant throughout serial replating to M4. Instead, in Pin1 silenced (+DOX) cells, MFE decreased already at M2 stage and progressively at M3 and M4. Similarly, pharmacological Pin1 inhibition shrunk mammosphere forming efficiency in a dose dependent manner in MDA-MB-231 and other breast cancer cell lines (BT-549 and SUM-159), (supplementary Fig S2A left and right). Pin1 inhibition impaired also self-renewal of CSCs derived from two aggressive primary breast cancers (supplementary Fig S2B), demonstrating that loss of Pin1 activity impairs breast CSC self-renewal and replicative potential in a broad spectrum of breast cancer cells. In fact, the content of putative stem cells was lower following Pin1 silencing or inhibition, as confirmed by the Aldefluor assay, that evaluates the activity of Aldehyde dehydrogenase 1 (Aldh), a marker for breast CSCs (Ginestier *et al*, 2007) (Fig 2B, right). Conversely, overexpression of Pin1 by retroviral infection of MDA-MB-231 cells increased M2 formation by almost twofold and produced an increase of Aldh-positive cells compared to empty vector harboring cells (supplementary Fig S2C). In agreement with a role of Pin1 in the maintenance of breast CSCs, its mRNA levels were found up-regulated in Aldh-positive as compared to Aldh-negative cells sorted from MDA-MB-231 M2 (supplementary Fig S2D).

Digging deeper into the effects of Pin1 depletion, we next evaluated the expression of several genes acting within pathways governing the stemness phenotypes of breast CSCs (Leong *et al*, 2007; Yu *et al*, 2007, 2011; Polyak & Weinberg, 2009; Cordenonsi *et al*, 2011; Visvader & Lindeman, 2012). As shown in Fig 2C (left panel), the expression of all tested factors (Hes1, HeyL, Birc5, CTGF, Slug, ABCG2, Ptch, Bmi-1, HMGA2 and Klf4) decreased by Pin1 knockdown.

Epithelial-mesenchymal plasticity in breast carcinoma has recently been linked to acquisition of stem cell traits by tumor cells (Mani *et al*, 2008). We therefore also analysed the impact of Pin1 modulation on this process by analyzing markers of epithelial-mesenchymal transition (EMT). Of note, Pin1 downmodulation caused enhanced mRNA expression of the epithelial marker E-cadherin (CDH1) while that of mesenchymal markers Vimentin and Fibronectin (VIM1, FN) was reduced (Fig 2C, left panel), in parallel with a strong recovery of E-cadherin and decay of Slug and Vimentin at the protein level (Fig 2C, right panel).

All together these results indicate that high Pin1 levels are required to sustain mesenchymal traits and to keep pro-stemness signaling constant.

### Pin1 is required for Notch-dependent induction and maintenance of stem cell self-renewal in normal and cancer cells of the breast

**Figure 3:**
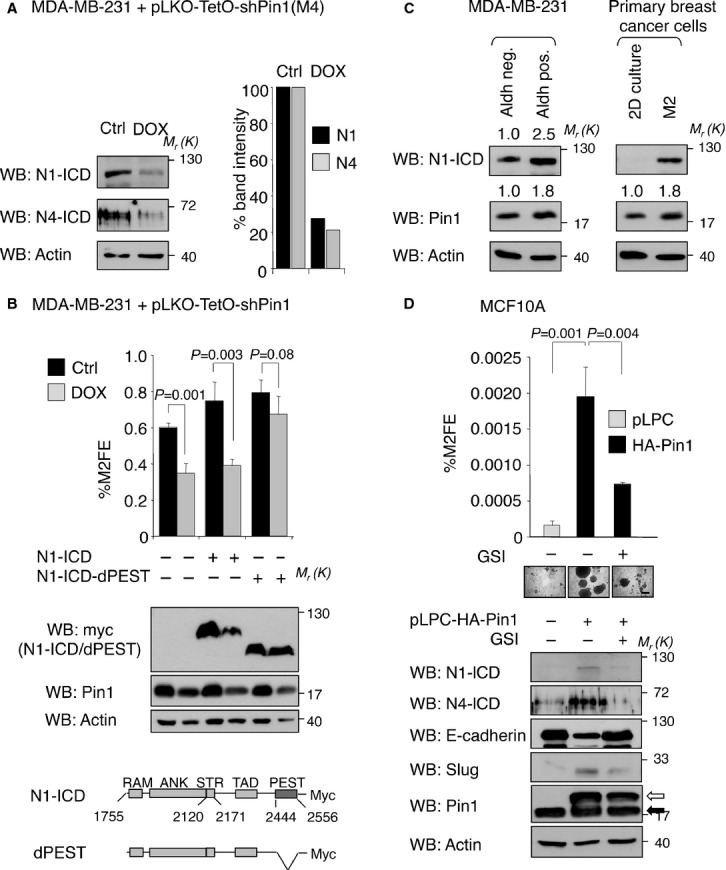
Pin1 controls breast CSC self-renewal through N1-ICD stabilization. A  Pin1 depletion causes reduced N1- and N4-ICD protein levels. Left panel: Western Blot analysis of N1- and N4-lCD protein from MDA-MB-231-pLKO-shPin1 M4 control cells (Ctrl) and shPin1 induced cells (DOX). Molecular weights (*M*_r_) are indicated in kDa. Right panel: histogram representing the percentage of band intensity with respect to actin levels. B  Expression of N1-ICD-dPEST stable mutant rescues M2FE following Pin1 depletion. Upper panel: Percentage of secondary mammosphere formation efficiency (%M2FE) of control (Ctrl, black bars) or Pin1 silenced (DOX, grey bars) cells, transduced with empty (–), N1-ICD or N1-ICD-dPEST vectors (+). Means, standard deviations and *P*-values (*t*-test, *n* = 3) are indicated. Middle panel: Western blot analysis of the indicated proteins from cells grown as M2. Lower panel: Scheme of protein domains of overexpressed N1-ICD forms. Numbering refers to Swissprot entry P46531. C  Pin1 and N1-ICD levels are upregulated in the breast CSC compartment. Comparative Western blot analyses of Aldh-positive (stem) versus Aldh-negative cells (non-stem) sorted from MDA-MB-231 M2 (left panel) and patient-derived breast cancer secondary M2 mammospheres (stem) versus cells cultured in adherence (2D) (right panel). Relative fold change in Pin1 or N1-ICD protein levels determined by Image J software with respect to actin levels is indicated by a number. D  Pin1 overexpression promotes stem cell phenotypes through regulation of the Notch pathway. Upper panel: %M2FE of MCF10A breast epithelial cells transduced with empty (pLPC) or HA-Pin1 overexpressing vectors (pLPC-HA-Pin1) and treated with DMSO (–) or 10 μM GSI. Means, standard deviations and *P*-values (*t*-test, *n* = 3) are indicated. Middle panel: Representative microphotographs of M2 are shown, scale bar of 200 μm is indicated. Lower panel: Western Blot of cell lysates from corresponding MCF10A clones. White and black arrows indicate over-expressed and endogenous Pin1, respectively.

The majority of genes described above are controlled by the Notch pathway (Lee *et al*, 2008; Ranganathan *et al*, 2011; Li *et al*, 2012), which was shown to be required for EMT induction (Leong *et al*, 2007) and regulation of both normal stem cells of the mammary gland and breast CSC (Dontu *et al*, 2004; Bouras *et al*, 2008; Raouf *et al*, 2008; Harrison *et al*, 2010; Xing *et al*, 2012). We therefore investigated whether the action of Pin1 in breast CSCs maintenance is driven by Notch function. Notch proteins are membrane-bound receptors, that upon ligand binding, are subjected to cleavage by gamma-secretase, releasing an intracellular domain (N-ICD) directly involved in transcriptional control (Ranganathan *et al*, 2011). In particular, two members of the family, Notch1 and Notch4, have been linked to induction and maintenance of breast CSC features (Farnie *et al*, 2007; Grudzien *et al*, 2010; Harrison *et al*, 2010). Notably, the levels of their active forms (N1-ICD and N4-ICD) were strongly reduced (about five fold) by Pin1 knockdown in M4 mammospheres compared to control cells (Fig 3A). The robust downregulation that we observed could not be totally ascribed to an inefficient cleavage of Notch1 at the membrane due to Pin1 downmodulation, as previously described (Rustighi *et al*, 2009). Rather, since the Notch ICDs are tightly regulated by proteasomal degradation we hypothesized that Pin1 could regulate Notch ICDs' stability.

To address the question whether the effect of Pin1 on breast CSC relies on its action on N1-ICD levels, we tested the ability of wild-type N1-ICD or of a constitutively stable N1-ICD mutant (dPEST) to rescue M2 formation following Pin1 knockdown. This mutant lacks the cdc4-phosphodegron constituting the consensus for the E3 ubiquitin-ligase Fbxw7α, the major negative regulator of the intracellular Notch signal (O'Neil *et al*, 2007; Thompson *et al*, 2007). As expected, M2FE of MDA-MB-231-pLKO-shPin1 cells decreased upon Pin1 silencing (+DOX) (Fig 3B). Notably, M2FE did not further increase following ectopic expression of N1-ICD in control cells, since in these cells endogenous Notch pathway is already strongly activated (Harrison *et al*, 2010). Moreover, N1-ICD overexpression was not able to rescue M2FE in Pin1 silenced cells. By contrast, overexpression of N1-ICD-dPEST was able to rescue M2FE. Consistent with this, protein levels of over-expressed N1-ICD, but not those of N1-ICD-dPEST, were strongly decreased upon Pin1 depletion (Fig 3B, lower panel). Similar results were obtained in SK-BR-3 cells when N1-ICD or N1-ICD-dPEST were ectopically expressed and the function of Pin1 blocked by the Pin1 inhibitor PiB (supplementary Fig S3A). These data suggest that Pin1 critically supports breast CSC self-renewal by sustaining high intracellular levels of N1-ICD. To strengthen this finding, we analysed the endogenous levels of both Pin1 and N1-ICD in CSCs. To this aim, we sorted the Aldh-positive cells from MDA-MB-231 mammospheres or collected patient derived mammospheres and analysed their protein content. As shown in Fig 3C (left and right panels) in both conditions the protein levels of Pin1 and N1-ICD were almost two times higher in the stem cell fraction as compared to those in their differentiated counterpart. Moreover we found that also *in vivo*, in breast cancer tissues, the expression of Pin1 colocalized with Aldh-positive cells (supplementary Fig S3B).

We next tested whether Pin1 alone is sufficient to confer stem cell traits in a Notch-dependent manner to a non-transformed epithelial breast cell line. To this aim, we engineered MCF10A cells to overexpress Pin1 and analysed them by FACS for expression of CD44/CD24 surface molecules, two other well-established breast stem cell markers (Al-Hajj *et al*, 2003). Pin1 overexpression caused an enrichment of the CD44high/CD24low stem cell population as compared to empty vector cells (supplementary Fig S3C). Accordingly, while empty vector transduced MCF10A cells hardly formed secondary mammospheres, Pin1 overexpression strongly induced their formation (Fig 3D). This was accompanied by an EMT as demonstrated by down- and upregulation of the two markers E-cadherin and Slug, respectively. Crucially, the levels of cleaved Notch1 and Notch4, normally undetectable in these cells, were upregulated by Pin1 overexpression and were essential for mammosphere formation, since block of Notch cleavage by a gamma-secreatase inhibitor (GSI) was sufficient to blunt this process (Fig 3D).

All together our results demonstrate that Pin1 is a *bona fide* stem cell factor by promoting EMT and maintaining a mesenchymal/stem cell fate mainly through regulation of the Notch pathway.

### Suppression of Pin1 sensitizes breast CSC to chemotherapy *in vitro* and *in vivo*

**Figure 4:**
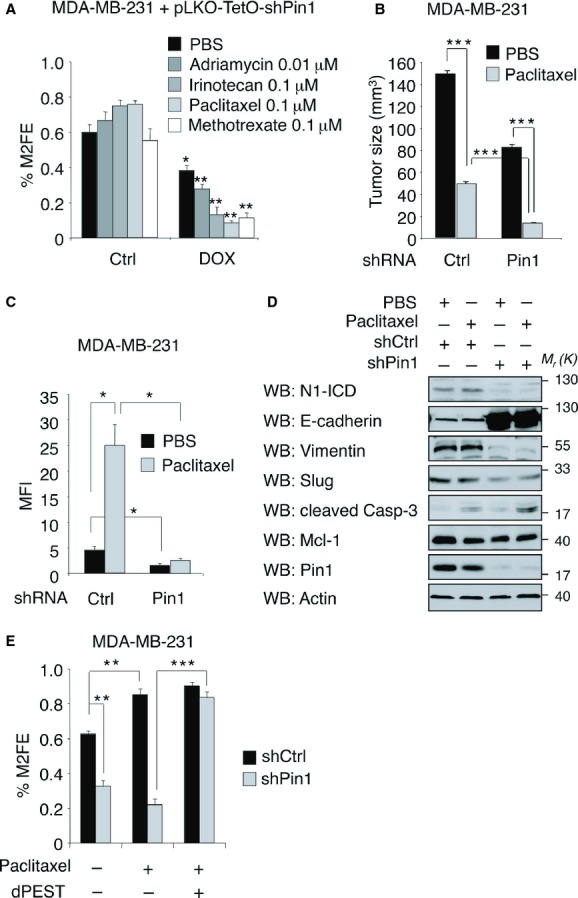
Pin1 downmodulation sensitizes breast CSCs to chemotherapeutic treatment in vitro and in *vivo*. A  Pin1 knockdown synergizes with chemotherapy treatment to block breast CSCs' self-renewal. Percentage of M2FE of control MDA-MB-231-pLKO-shPin1 cells (Ctrl) compared to shPin1-induced cells (DOX) treated with indicated drugs or PBS. Means and standard deviations are indicated, *P*-values are * = 0.001, **< 0.0003 (*t*-test, *n* = 3). B  Pin1 knockdown synergizes with paclitaxel to block breast cancer growth. Tumor growth of MDA-MB-231 xenografts espressing the indicated shRNAs and treated with paclitaxel (grey bars) or left untreated (PBS) (black bars). Means and standard deviations are indicated, *P*-values are *** < 0.0003 (*t*-test, *n* = 12). C  Pin1 knockdown blocks chemotherapy-induced breast CSCs' expansion *in vivo*. Histogram representing the Aldefluor mean fluorescent intensity (MFI) of cells from control- and shPin1 MDA-MB-231 xenografts treated with Paclitaxel or PBS. Means and standard deviations are indicated, *P*-values are * = 0.001 (*t*-test, *n* = 3 for each condition). D  Pin1 knockdown induces reversal of EMT and cell death in breast cancer xenografts in combination with paclitaxel. Western blot analyses of tumor xenografts from (B). E  Expression of stable N1-ICD-dPEST rescues resistance to paclitaxel treatment in Pin1 silenced cells. Percentage of M2FE of control (black bars) or Pin1 shRNA (grey bars) expressing MDA-MB-231 cells transduced with empty (–) or N1-ICD-dPEST (+) expressing vectors, treated with Paclitaxel (+) or PBS (–). Means and standard deviations are indicated, *P*-values are **0.0001, ***<0.00003 (*t*-test, *n* = 3).

Breast CSCs and cells that have undergone an EMT exhibit increased drug resistance (Dean *et al*, 2005; Gupta *et al*, 2009). In addition, treatment of cancers with chemotherapeutic agents has been shown to cause enrichment of CSCs (Yu *et al*, 2007; Levina *et al*, 2008). This evidence prompted us to investigate the consequences of Pin1 ablation on breast CSCs' drug resistance. To this aim we analysed the efficiency of M2 formation of MDA-MB-231-pLKO-shPin1 cells in presence of different chemotherapeutic agents (Fig 4A). As expected, treatment of control cells with adriamycin, paclitaxel, irinotecan or methotrexate, elicited a slight increase or had no effect on M2 formation. Instead, Pin1 silencing, which alone had already a negative impact on mammosphere formation, elicited a strong synergistic toxicity in combination with all four compounds, as demonstrated by the consistent drop of M2 formation efficiency in all conditions.

To test the *in vivo* impact of these findings, we injected MDA-MB-231 cells, stably expressing a control- or a Pin1-specific shRNA, into the inguinal mammary fat pads of immunocompromised mice. When tumors became visible, each group was randomized and treated with either paclitaxel or PBS and tumor growth was monitored for two more weeks. As shown in Fig 4B, the size of Pin1 specific shRNA expressing tumors reached half of those with control shRNA and treatment with paclitaxel induced a significant growth inhibition in both conditions. We next analysed the CSC content (Aldh-pos) in these tumors. As shown in Fig 4C, in paclitaxel treated control tumors the Aldh-positive cell population was heavily increased with respect to that derived from PBS treated xenografts (Fig 4C). In contrast, tumors in which Pin1 was silenced were characterized by a drastic impoverishment of Aldh-positive CSCs in both treatment conditions, indicating that high Pin1 levels are required for chemotherapy-induced CSCs expansion. We next analysed protein lysates from these tumors by Western blot. As shown in Fig 4D, we detected a reversal of the EMT phenotype in shPin1 expressing tumors, as evidenced by increased E-cadherin and decreased Slug and Vimentin levels. Accordingly, N1-ICD levels were downregulated in these tumors. In addition, paclitaxel treatment induced caspase-3 cleavage that was strongly increased by Pin1 silencing, indicating that reduced levels of Pin1 synergize with paclitaxel in inducing apoptosis and hence causing maximal tumor shrinkage. Mcl-1 is an important mediator of paclitaxel resistance in breast cancer and other tumors and a critical prosurvival protein (Ding *et al*, 2008; Inuzuka *et al*, 2011; Wertz *et al*, 2011). Being also stabilized by Pin1 (Ding *et al*, 2008), we wondered whether Mcl-1 downmodulation could be involved in apoptosis induction upon Pin1 silencing and paclitaxel treatment. This experiment indicated that Mcl-1 levels were indeed affected either by Pin1 silencing or paclitaxel treatment. However, in xenografts silenced for Pin1 and treated with paclitaxel, we did not observe further changes in Mcl-1, indicating that other important mediators of chemoresistance were involved in this context. Nevertheless, these effects appeared to be Notch-dependent, since MDA-MB-231 cells treated with two different Pin1 shRNAs and transduced with a N1-ICD-dPEST expressing vector were able to fully regain chemoresistance, as shown by recovery of M2FE in presence of the chemotherapeutic agent (Fig 4E and supplementary Fig S4A).

All together these results indicate that Pin1 is crucial in maintaining the self-renewal and replicative potential of both normal stem cells and CSCs of the mammary gland and that genetic or pharmacological ablation of Pin1 impairs their expansion with a strong effect in CSCs, eliciting sensitivity to chemotherapeutic drugs.

### Pin1 regulates N1-ICD stability through the cdc4-phosphodegron

**Figure 5:**
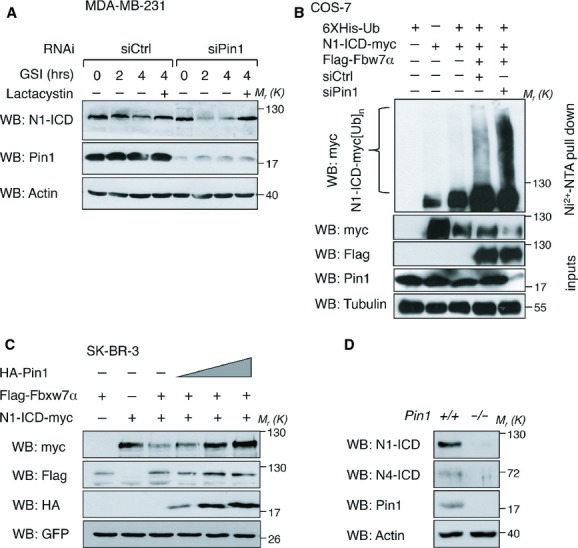
Pin1 rescues N1- and N4-ICD from Fbxw7α-mediated proteasome-dependent degradation. Molecular weights (*M*_r_) are indicated in kDa. A  Pin1 knockdown accelerates the decay of endogenous N1-ICD. Western blot of endogenous N1-ICD following RNA interference (RNAi) with the indicated siRNAs and time points following GSI or GSI plus Lactacystin (+) chase is shown. B  Pin1 depletion enhances Fbxw7α-dependent poly-ubiquitination of N1-ICD. Western blot analysis of high molecular weight N1-ICD-myc products (N1-ICD-myc[Ub]_*n*_) from a Ni-NTA pull-down in COS-7 cells transfected with the indicated vectors along with control- or Pin1 siRNA. Input levels of over-expressed proteins are shown. C  Pin1 overexpression rescues N1-ICD levels in presence of Fbxw7α. Western blot analysis of lysates from SK-BR-3 cells over-expressing N1-ICD-myc, Flag-Fbxw7α along with empty (–) or increasing amounts of HA-Pin1 expressing vector, normalized for co-expressed GFP protein. D  *Pin1*^*−/−*^ mammary epithelial cells have impaired Notch pathway activation. Western blot analyses of primary MECs from indicated female mice.

We next analyzed in more detail the impact of Pin1 on N1-ICD protein stability. As shown in Fig 5A, Pin1 depletion by siRNA in MDA-MB-231 breast cancer cells treated with gamma-secretase inhibitor to blunt Notch cleavage at the membrane (Ranganathan *et al*, 2011), caused a strong decay of cytoplasmic N1-ICD. This effect was proteasome-dependent since N1-ICD levels were rescued following addition of the proteasome inhibitor Lactacystin. Also in the SK-BR-3 breast cancer cell line the half-life of overexpressed N1-ICD was strongly reduced from 2 h to 40 min upon Pin1 siRNA treatment with respect to control silencing (supplementary Fig S5A). Importantly, reintroduction of a siRNA resistant Pin1 construct (Pin1r) in Pin1 depleted cells almost completely reversed this effect, demonstrating that N1-ICD levels are Pin1 dependent.

Pin1 is known to bind several substrates on phosphorylated TP or SP residues and to affect their stability (Liou *et al*, 2011). *In vitro* and *in vivo* binding experiments demonstrated that Pin1 binds to N1-ICD and this interaction was direct (supplementary Fig S5B and C). Moreover, domain mapping analysis showed a marked reduction in Pin1 binding to N1-ICD when the C-terminal PEST domain containing the cdc4-phosphodegron was deleted (supplementary Fig S5D). Intriguingly, the residues T2512/P2513 within the phosphodegron, known to be critical for recognition by the ubiquitin-ligase Fbxw7α upon phoshorylation of T2512 (O'Neil *et al*, 2007; Thompson *et al*, 2007), might also become a Pin1 binding site. Indeed, when we performed *in vitro* GST-Pin1 binding assays with N1-ICD or a point mutant replacing the threonine residue 2512 with alanine (T2512A), the interaction was decreased (supplementary Fig S5E). Moreover Pin1 overexpression did not affect the levels of this mutant in protein stability assays, as it did instead with wild-type N1-ICD (supplementary Fig S5F). All together these results raise the possibility that, when phosphorylated, aminoacids T2512/P2513 constitute the docking site for Pin1, which might exert N1-ICD stabilization by interfering with Fbxw7α-binding.

### Pin1 sustains N1- and N4-ICD levels *in vitro* and *in vivo* despite expression of Fbxw7α

To test whether the accumulation of nuclear N1-ICD could depend on the ability of Pin1 to antagonize Fbxw7α recognition and activity on N1-ICD, we evaluated the effect of Pin1 on Fbxw7α-dependent poly-ubiquitination of N1-ICD by Ni-NTA pull down in COS-7 cells (Fig 5B). As expected (O'Neil *et al*, 2007; Thompson *et al*, 2007), co-transfection of N1-ICD and Fbxw7α caused an enhancement of poly-ubiquinated N1-ICD as compared to empty vector control. Conversely, simultaneous depletion of Pin1 markedly increased ubiquitin conjugation, demonstrating that Pin1 antagonizes Fbxw7α-mediated N1-ICD poly-ubiquitination. Moreover, in MDA-MB-231 cells, reduced N1-ICD levels caused by Pin1 knockdown, were recovered by concomitant Fbxw7α silencing, thus indicating that Pin1 action was epistatic to Fbxw7α for N1-ICD stabilization (supplementary Fig S5G). Accordingly, when N1-ICD and Fbxw7α were ectopically expressed in SK-BR-3 cells and in *Pin1*^*−/−*^ mouse embryo fibroblasts, introduction of Pin1 efficiently rescued N1-ICD levels in the presence of the E3 ubiquitin-ligase (Fig 5C and supplementary Fig S5H). Moreover, N1-ICD stabilized by Pin1 over-expression was fully efficient at inducing transcription of target promoters, despite presence of functional Fbxw7α (supplementary Figs S5I and J).

The cdc4-phosphodegron is present also in N4-ICD (supplementary Fig S5K, upper part) and has been shown to be targeted for degradation by Fbxw7α (Wu *et al*, 2001). Pin1 bound efficiently also to N4-ICD, and silencing of Pin1 accelerated N4-ICD's proteasome-dependent degradation (supplementary Fig S5K, middle and lower panels). Accordingly, Pin1 over-expression enhanced both the levels and transcriptional activity of N4-ICD towards RBP-Jκ/LUC in the presence of over-expressed Fbxw7α (supplementary Fig S5L).

To confirm *in vivo* that N1- and N4-ICDs levels were Pin1-dependent we performed immunohistochemical analyses of mammary ducts from 8 weeks old virgin *Pin1*^*+/+*^ and *Pin1*^*−/−*^ mice. Supplementary Fig S5M clearly shows that while endogenous Fbxw7α protein levels remained unperturbed, the staining of both nuclear Notch1 and Notch4 was lower in *Pin1*^*−/−*^ mammary ducts as compared to those of *Pin1*^*+/+*^ mice. These results were quantitatively confirmed by Western blot analyses of mammary epithelial cell lysates obtained from *Pin1*^*+/+*^ and *Pin1*^*−/−*^ mammary ducts (Fig 5D).

Pin1 was previously shown to be a direct transcriptional target of N1-ICD (Rustighi *et al*, 2009), therefore we asked if N4-ICD might be a Pin1 regulator as well. By performing chromatin-immunoprecipitation and luciferase assays of the human Pin1 promoter (supplementary Figs S5N and O) we demonstrated that Pin1 is a direct N4-ICD transcriptional target. All together these data demonstrate *in vitro* and *in vivo* that Pin1 is critical for regulating the levels of both N1 and N4-ICDs. Moreover the degradation of both N1- and N4-ICDs by Fbxw7α can be efficiently overcome by Pin1, which restores their functional transcriptional activity, demonstrating the existence of a feed-forward molecular circuitry between Pin1 and the intracellular forms of both Notch1 and Notch4.

### Pin1 isomerase activity protects N1-ICD from Fbxw7α-dependent degradation

**Figure 6:**
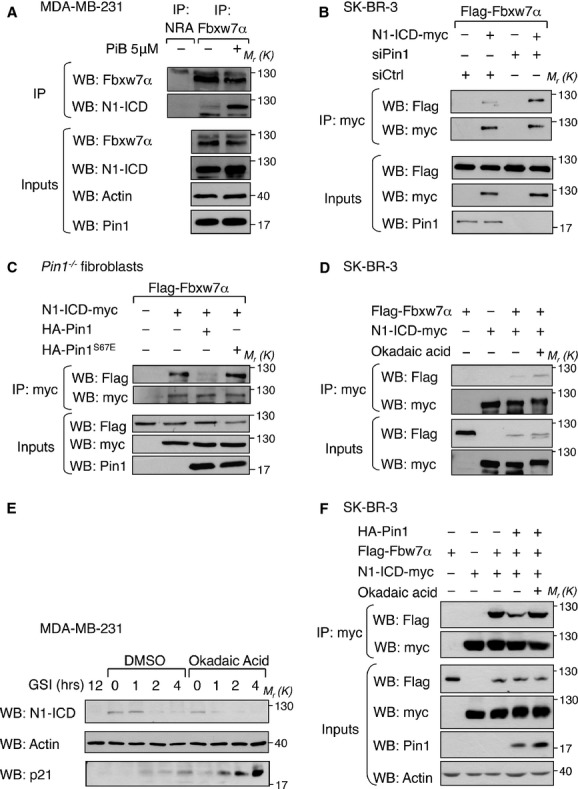
Pin1 controls N1-ICD half-life by uncoupling it from Fbxw7α. Experiments in (A)-(D) and (F) were performed in presence of proteasome inhibitor to avoid misinterpretation of binding results due to protein degradation. (A–F) Molecular weights (*M*_r_) are indicated in kDa. A  Inhibition of Pin1 increases interaction between endogenous Fbxw7α and N1-ICD proteins. Western blot analysis of co-immunoprecipitation (Co-IP) experiments between endogenous Fbxw7α and N1-ICD from MDA-MB-231 cells treated with DMSO (–) or PiB (+). Anti-Fbxw7α or non related antibody (NRA) immunoprecipitates (IP) were recognized with anti-N1-ICD (Val1744) antibody and after stripping with an anti-Fbxw7α antibody. Input levels are shown below. B  Depletion of Pin1 increases Fbxw7α-N1-ICD interaction. Representative Western blot analysis of Co-IP experiments between over-expressed N1-ICD-myc and Flag-Fbxw7α in SK-BR-3 cells. Over-expressed N1-ICD-myc was immunoprecipitated (IP) and subjected to anti-Flag Western Blot to reveal Flag-Fbxw7α Co-IP. Input levels of over-expressed or silenced proteins are shown below. C  Pin1 catalytic activity is required to uncouple N1-ICD from Fbxw7α. Co-IP as in (B) in *Pin1*^*−/−*^ embryo fibroblasts transduced with the indicated vectors. D  Inhibition of PP2A enforces Fbxw7α-N1-ICD interaction. Co-IP as above in SK-BR-3 cells treated with DMSO (–) or okadaic acid and transduced with the indicated vectors. E  Inhibition of PP2A accelerates the half-life of endogenous N1-ICD. Western blot analysis of a GSI chase of MDA-MB-231 cells treated with vehicle (DMSO) or okadaic acid. Anti-p21^Cip1Waf1^ immunoblot was added as control for Okadaic acid functioning (Park *et al*, 2001). F  PP2A is required for Pin1-dependent N1-ICD detachment from Fbxw7α. Co-IP as above in SK-BR-3 cells transduced with the indicated vectors and treated with DMSO (–) or okadaic acid.

To dissect the molecular mechanism governing the antagonistic interplay between Pin1 and Fbxw7α, we investigated the impact of Pin1 on the N1-ICD-Fbxw7α or N4-ICD-Fbxw7α association. As judged by co-immunoprecipitation (Co-IP) experiments performed with both endogenous and overexpressed proteins, we consistently observed increased binding between Fbxw7α and N1-ICD or N4-ICD upon inhibition of Pin1 activity by the small molecule inhibitor PiB (Fig 6A and supplementary Fig S6A) or depletion by siRNA (Fig 6B and supplementary Fig S6B). Importantly, in *Pin1*^*−/−*^ mouse embryo fibroblasts, expression of Pin1, but not of the catalytic inactive mutant Pin1^S67E^ (Rustighi *et al*, 2009), reduced their association (Fig 6C). Like wild-type Pin1, Pin1^S67E^ interacts with N1-ICD (supplementary Fig S6C), therefore these data clearly indicate that it is the isomerase activity of Pin1 that is crucial to oppose N1-ICD-Fbxw7α interaction, ruling out the possibility of a mere competition between the two enzymes for binding to the phosphorylated sites within the cdc4-phosphodegron.

Prolyl-isomerization of specific phospho-S/T-P sites leads, on certain substrates, to recognition and subsequent dephosphorylation by the trans-specific phosphatase PP2A (Liou *et al*, 2011). We therefore hypothesized that, following isomerization by Pin1, the Notch cdc4-phosphodegron could be dephosphorylated by PP2A, thus eluding recognition by Fbxw7α. To evaluate this possibility we assessed the interaction between N1-ICD and Fbxw7α by co-imunoprecipitation in SK-BR-3 cells treated with PP2A inhibitor okadaic acid or silenced for PP2A expression. As shown in Fig 6D and supplementary Fig S6D, the interaction between N1-ICD and Fbxw7α increased when cells were treated with okadaic acid or upon PP2A silencing, with consequent reduction of the levels and half-life of endogenous N1-ICD (Fig 6E). We next analyzed the dynamics of this interaction following Pin1 overexpression in the same conditions as above. As shown in Fig 6F and supplementary Fig S6E, Pin1 expression consistently reduced the interaction between N1-ICD and Fbxw7α but only in the presence of functionally active PP2A, indicating that PP2A is required for Pin1-dependent N1-ICD accumulation.

### Pin1 and Fbxw7α control Notch1/4-dependent stem cell self-renewal and frequency in an antagonistic fashion

**Figure 7:**
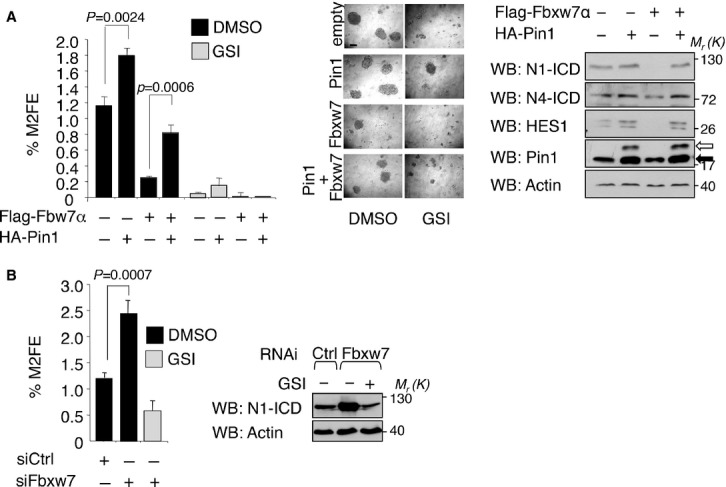
Pin1 and Fbxw7α modulate N1-ICD stem cell activity in *vitro*. A  Fbxw7α overexpression reduces breast CSCs self-renewal. Left panel: %M2FE of MDA-MB-231 cells overexpressing the indicated vectors. Black and grey bars indicate DMSO or GSI treated mammospheres, respectively. Means, standard deviations and *P*-values (*t*-test, *n* = 3), are indicated. Middle panel: Representative microphotographs of M2 are shown, 200 μm scale bar is indicated. Right panel: Western Blot of cell lysates from M2. White and black arrows indicate over-expressed and endogenous Pin1, respectively. B  Fbxw7α genetic ablation increases breast CSCs' self-renewal in a Notch-dependent manner. Left panel: Histogram showing %M2FE of MDA-MB-231 cells with indicated siRNA and treated with vehicle (black bars) or GSI (grey bars). Means, standard deviations and *P*-values (*t*-test, *n* = 3), are indicated. Right panel: Western Blot of cell lysates from M2. Molecular weights (*M*_r_) are indicated in kDa.

Having dissected the biochemistry of the Pin1/Fbxw7α antagonism in the Notch pathway, we next investigated the impact of this interplay on the stem cell functions of Notch signaling in breast cancer cells. We performed secondary mammosphere (M2) formation assays in MDA-MB-231 cells ectopically expressing Fbxw7α and Pin1. As shown in Fig 7A and supplementary Fig S7A, the efficiency of mammosphere formation, the levels of both N1- and N4-ICD and the size of the Aldh-positive cell population were all curbed in Fbxw7α over-expressing cells as compared to the empty vector control. Of note, this effect was almost completely rescued by simultaneous expression of Pin1, which recovered both N1- and N4-ICD protein levels and transcriptional activity towards endogenous targets (HES-1 and Pin1, Fig 7A, right panel). These effects were Notch-dependent, as indicated by the decrease, in all conditions, of M2 formation efficiency upon administration of GSI or Notch1 specific siRNA (Fig 7A and supplementary Fig S7B).

As shown above, Fbxw7α had a potent inhibitory effect on CSCs. Since Fbxw7α acts on several important targets (Wang *et al*, 2011) we assessed the relevance of Notch activity for this phenotype. As shown in Fig 7B downmodulation of Fbxw7α caused a consistent increase of N1-ICD levels and spured M2FE and treatment with GSI elicited a strong reduction of both mammosphere formation and Notch pathway activation (Fig 7B and supplementary Fig S7C), thus demonstrating that among several Fbxw7α targets, Notch is the critical one in this cellular context.

Next, to demonstrate the impact of Pin1 and Fbxw7α interplay on CSC frequency *in vivo*, we injected increasingly diluted single-cell preparations in the inguinal mammary gland of SCID mice (Table 1). At 300 000 transplanted cells, only MDA-MB-231 cells transduced with empty vector still developed palpable tumors in all mice, while Fbxw7α over-expressing cells originated tumors in only two out of nine. Cells overexpressing Pin1 and Fbxw7α, instead, showed a significant recovery of tumor formation, since tumors grew in seven out of nine mice, a trend that was observed also for the subsequent dilution of 150 000 cells.

**Table 1:** Frequency of CSCs in MDA-MB-231 xenografts.

	1 000 000	600 000	300 000	150 000
Empty	6/6	9/9	9/9	12/15
Fbxw7α	6/6	9/9	2/9	1/15
Fbxw7α + Pin1	6/6	7/8	7/9	5/15
	Estimate (0.95)	Lower–Upper	*P* value	*P* value
Empty	1:144 232	1:84 380–1:49 364		
Fbxw7α	1:776 940	1:485 184–1:302 989	1.58 × 10^–6^	
Fbxw7α + Pin1	1:466 587	1:302 232–1:195 771	1.04 × 10^–2^	0.0239

Mice were transplanted with decreasing numbers of MDA-MB-231 cells overexpressing empty, Fbxw7, or Fbxw7 + Pin1 vectors (number of injected cells is indicated). Results are shown as the number of tumors per number of injected mice (upper panel). CSC frequencies (estimates and upper/lower limits) were calculated by limiting dilution analysis, as described in Materials and methods. Differences in CSC frequencies are indicated for each sample against the empty vector and for Fbxw7 + Pin1 against Fbxw7 only. Their significance is indicated by a *P* value (lower panel).

**Figure 8:**
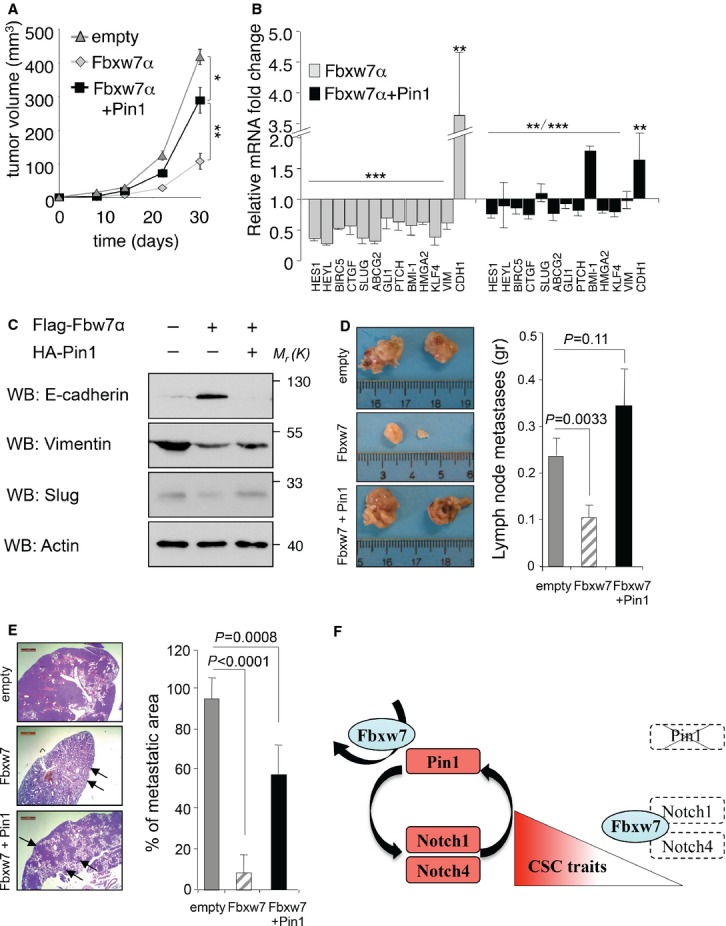
Pin1 and Fbxw7α modulate N1-ICD stem cell activity in vivo. (A), (B) and (D), (E) Means, standard errors of the mean and *P*-values (*t*-test, *n* = 6), are indicated. (C) Molecular weights *M*_r_(K) are indicated in kDa. A  Pin1 rescues tumor growth in Fbxw7 overexpressing xenografts. Tumor volume of orthotopic xenografts in SCID mice obtained from the indicated MDA-MB-231 cell clones. B   Pin1 rescues stem cell marker expression in Fbxw7 overexpressing xenografts. qRT-PCR of Fbxw7α and Fbxw7α + Pin1 tumor xenografts relative to control tumors (empty) explanted at the end of the experiment. C   Pin1 recovers EMT marker expression in Fbxw7 overexpressing xenografts. Western blot analyses of the indicated proteins from the same tumor xenografts as in (A). D,E  Pin1 rescues metastasis formation of Fbxw7 overexpressing primary tumors. Comparison of lymph node and pulmonary metastases growth derived from the above xenografts, respectively. Representative images of colonized lymph nodes (D) and hematoxylin and eosin stained pulmonary sections (E) are shown. Rulers and Scale bars (1 mm) are indicated for calibration, arrows indicate metastatic areas. F  Schematic representation summarizing the role of Pin1 in sustaining CSCs through Notch1 and Notch4 by antagonizing Fbxw7α-mediated destruction.

We further explored the behavior of these cells in tumor growth and dissemination *in vivo*. To this aim, we injected 1 000 000 cells/flank to allow tumor formation in all transplanted mice. Compared to control, MDA-MB-231 cells overexpressing Fbxw7α produced only small tumors with decreased levels of N1-ICD, reduced expression of a series of Notch1 target genes, loss of mesenchymal and recovery of epithelial markers (Fig 8A–C and supplementary Fig S8A). Conversely, Pin1 overexpression bypassed the tumor suppressor function of Fbxw7α, as demonstrated by tumors co-expressing both proteins, that displayed increased volume, rescued N1-ICD levels, its transcriptional activity on target genes as well as the levels of mesenchymal markers. Consistently, Pin1 overexpression in Fbxw7α overexpressing cells recovered also the ability of tumors to develop metastasis into nearby lymph nodes and to the lungs (Fig 8D and E).

### Aberrant Notch signaling in primary breast cancers is sustained by Pin1 in spite of Fbxw7α expression

In human T-cell acute lymphoblastic leukaemia and lymphoma (T-ALLs) aberrant Notch activity is caused by mutations of *NOTCH1* and *FBXW7* in more than 50% of patients (Ferrando, 2009). Like in T-ALLs, also in human patients with breast cancer high expression of Notch receptors and ligands is causally involved and has been linked to poor clinical outcomes (Han *et al*, 2011; Xu *et al*, 2012). However, according to Cosmic, CONAN and TCGA databases and recent publications, these genes are rarely mutated in breast cancer (ranging from 2 to 8% depending on the analysis) (Byrd *et al*, 2008; Mao *et al*, 2008; Ibusuki *et al*, 2011; Santarpia *et al*, 2012), raising the possibility that in this context, in the absence of mutations, high Pin1 expression might contribute to sustain levels and function of nuclear N1- and N4-ICD by interfering with their degradation by Fbxw7α.

To evaluate this hypothesis, we analysed tissue sections from two groups of breast cancer patients. The first derived from 43 breast cancers, previously characterized for the amount of activated N1-ICD protein by IHC (Vermezovic *et al*, submitted) and the second group consisting of 38 breast cancer tissues of the triple negative (TNBC) subtype. The first group was analysed by quantitative RT-PCR for the expression levels of *PIN1* and *FBXW7* mRNA (supplementary Fig S9A, and B), known to provide a direct correspondence to their protein levels (Girardini *et al*, 2011; Ibusuki *et al*, 2011). Samples were grouped into high or low N1-ICD expressing cases and, in agreement with previous IHC data on other cohorts (Farnie *et al*, 2007; Rustighi *et al*, 2009), N1-ICD levels were high in 40% (17/43 cases) of the analyzed tumors (supplementary Fig S9A). Of note, in 65% of these cases (11/17 cases) we found also high *FBXW7* expression. Strikingly, all the samples simultaneously displaying high N1-ICD and high *FBXW7* expression, also expressed high levels of *PIN1* (supplementary Fig S9A and B), indicating that in a consistent proportion of breast cancer patients (11/43 cases) high N1-ICD levels coexist with Fbxw7α thanks to high Pin1 expression.

**Figure 9:**
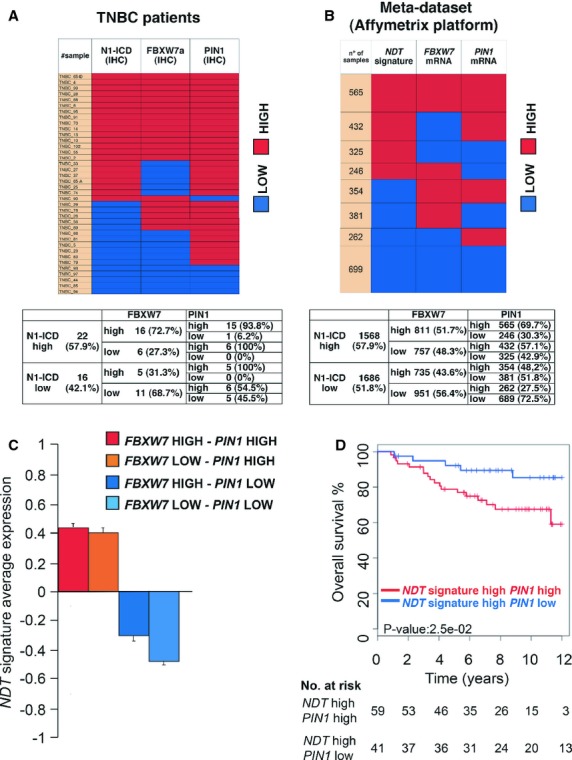
High N1-ICD levels in human breast cancers coexist with Fbxw7 thanks to high Pin1 expression. A  Immunohistochemical analysis of the indicated proteins in a panel of 38 triple negative breast cancer samples. Upper panel: heatmap representing the protein levels of Pin1, Fbxw7α and N1-ICD in a cohort of 38 breast cancer patients. The colors represent high (red) or low (blue) protein levels according to protein expression scores (see supplementary Methods). Lower panel: Contingency table showing percentage of each category calculated on the precedent category of patients; chi-square test was performed for independence between the variables and the *P*-value = 10^–5^. B  *NDT* expression analysis in a Meta-dataset of 3254 breast cancer patients. Upper panel: heatmap representing the contingency table frequencies of samples classified as having high or low levels of *FBXW7*, of *PIN1* and of the *NDT* gene signature. Number of samples in each category is indicated on the left. The association among high levels of *NDT* gene signature, *PIN1*, and *FBXW7* resulted statistically significant (*P *<* *0.001; chi-square test). Lower panel: Contingency table showing percentage of each category calculated on the precedent category of patients. C  Expression correlation between NDT and PIN1 and FBXW7 mRNA levels. Average expression of *NDT* gene signature in breast cancer samples stratified according to high or low expression of *PIN1* and *FBXW7* mRNA. Data are shown as mean ± standard error of the mean (s.e.m.). D  Survival analysis of Grade 3 high *NDT* expressing patients in function of *PIN1* expression. Kaplan–Meier survival curve is indicated for high *NDT* signature, grade 3 breast cancer patients of the metadataset in function of high or low *PIN1* mRNA levels. *P*-value and the number of subjects at risk at each time point is indicated below.

We next stained serial sections of the second group (38 TNBC samples) of breast cancer tissues with anti-N1-ICD, anti-Pin1 and anti-Fbxw7 antibodies (Fig 9A and supplementary Fig S9C). Among 22 patients with high intracellular Notch1 immunoreactivity we found a high percentage of patients with a strong nuclear Fbxw7α signal (72.7%). Again, the majority of these samples (93.8%) also displayed high Pin1 levels, that might be responsible for the simultaneous presence of high N1-ICD and its ub-ligase (Fig 9A and supplementary Fig S9C).

We finally confirmed these results on a larger cohort of patients by analyzing a meta-dataset of 19 independent breast cancer expression studies, collectively consisting of 3254 individuals (see supplementary Data S1 and Table S1). Specifically we used the classifier described in Adorno *et al* (2009) to stratify patients into groups of tumors expressing high or low levels of *FBXW7* and *PIN1* mRNA. Considering that mRNA levels of Notch receptors are frequently not representative of the protein levels of N1-ICD, activated Notch1 pathway status in this cohort was inferred from expression levels of a Notch-dependent gene signature, built up by selecting published Notch1 targets, for which Notch responsiveness and/or direct promoter binding as well as their expression in breast cancer was demonstrated (Notch direct target gene signature, *NDT,* supplementary Table S2). More than 48% of all samples expressed high levels of *NDT* signature genes and this correlated with poorer overall survival (Fig 9B and supplementary Fig S9D), consistent with previously published analyses (Farnie *et al*, 2007) and confirming the usefulness of this signature as a surrogate of activated Notch1 pathway. Interestingly, we found high *FBXW7* levels expression in 51.7% of patients with hyperactive Notch (Fig 9B). Similarly to the findings in the cohorts of Fig 9A and supplementary Fig S9A, we found a high correlation with *PIN1* overexpression in the great majority of these cases, while those with low *PIN1* expression were underrepresented with respect to any other possible category of patients with high levels of *FBXW7* mRNA (Fig 9B). Notably, the average expression value of *NDT* gene signature was contingent on *PIN1* mRNA levels (Fig 9C), while *FBXW7* was non influential, therefore highlighting the biological dominance of Pin1 over Fbxw7α in regulating Notch signaling in breast cancer. To evaluate the clinical significance of this finding we searched for the effect of high or low Pin1 expression levels on the survival of patients with high or low *NDT* signature. While Pin1 levels did not affect the clinical outcome in all patients, we found that in grade 3 breast cancer high Pin1 levels correlate with a worse outcome in patients with activated Notch1 signature (high *NDT*-high *PIN1*, Fig 9D), but not in patients with low *NDT* signature expression (supplementary Fig S9E). Moreover, pathway enrichment showed association with several stem cell pathway signature genes in this group of patients (high *NDT*-high *PIN1,* supplementary Table S3).

## Discussion

A consistent body of evidence accumulated in the past years supports the relevance of Pin1-mediated prolyl *cis*/*trans* isomerization in controlling the fate of phosphoproteins, hence a wide spectrum of cellular events (Liou *et al*, 2011). In this study a novel role for this unique isomerase is highlighted in the maintainance of stem cell traits of both normal SCs and CSCs of the breast.

Normal and CSCs manifest considerable similarities regarding the molecular pathways and factors that determine their undifferentiated state and their expansion (Visvader & Lindeman, 2012). We present here a scenario in which, through modulation of Notch signaling, Pin1 fine-tunes stem cell pathways in the normal gland and is fundamental for the induction of mouse and human mammary CSCs. Reduction of its levels, or inhibition of its activity, in this context, is sufficient to revert stem cell traits and rescue sensitivity to chemotherapeutic agents *in vitro* and *in vivo*. We have also shown that stem cell and EMT markers are subjected to Pin1 levels, providing a molecular basis for the observed phenotypes.

It has been shown that Pin1 and Notch1 are mediators of chemoresistance (Ding *et al*, 2008; Ranganathan *et al*, 2011; Domingo-Domenech *et al*, 2012), another critical trait of CSCs. Notch1, in particular, plays a major role in this process, since it promotes the expression of cell survival (e.g. *BIRC5* or *SURVIVIN* and *BCL-2*) and drug efflux pump genes (e.g. *ABCG2*) (Bhattacharya *et al*, 2007; Ranganathan *et al*, 2011). It is conceivable that the regained sensitivity of CSCs to drug treatment that we observed *in vitro* and *in vivo* by downregulation of Pin1 could be the consequence of reduced cell survival and drug efflux that are under direct control of Notch1 (Fig 4).

In our animal models, the stemness properties governed by Pin1 and Notch1 match well with their tumorigenic and metastatic potential. In parallel, in G3 breast cancers, high Pin1 levels are associated with a worse clinical outcome in patients with high *NDT* signature (Fig 9D). Pin1 levels have been clearly demonstrated to correlate with tumor grade (Wulf *et al*, 2001). This evidence coupled with the observation that G3 aggressive and poorly differentiated tumors, characterized by bad prognosis, display the highest stem cells' content (Pece *et al*, 2010), suggest that high Pin1 levels and activated Notch foster tumor progression by safeguarding self-renewal and expansion of their CSCs pool.

The influence of Pin1 on the Notch pathway is likely not limited to its direct action on Notch proteins. Indeed, among Notch targets several are also Pin1 substrates (e.g. cyclin D1, NF-κB, Survivin) (Wulf *et al*, 2005; Cheng *et al*, 2013), suggesting that in addition to its role in sustaining Notch1 protein levels, Pin1 could promote breast cancer aggressiveness also by enhancing the activity of some Notch-induced Pin1 targets.

Notch proteins sustain breast CSC and drive breast cancer progression primarily as a consequence of aberrant levels of their intracellular domains, resulting in constitutive signaling (Ranganathan *et al*, 2011). According to the current model, in normal conditions, after recruitment of p300 acetyl-transferase to the Mastermind-N1-ICD complex on target promoters, phosphorylation of the N1-ICD cdc4-phosphodegron causes recognition by the Fbxw7α E3 ubiquitin-ligase, leading to extinction of the Notch cascade as a fail safe mechanism against prolonged signaling (Ranganathan *et al*, 2011). The fact that this event is crucial in switching off the Notch signal, is witnessed by the significant percentage (more than 50% of the cases) of T-ALLs that loose this control due to mutations hitting *NOTCH1* or *FBXW7* (Ferrando, 2009). Our findings provide new insights unveiling a mechanism centered on phosphorylation-dependent prolyl-isomerization by Pin1 as responsible for sustained Notch signaling in breast cancer cells, regardless of Fbxw7α status. We show that isomerization of N1- and N4-ICD induced by interaction with Pin1 blocks recognition and degradation by Fbxw7α in a PP2A-dependent manner.

The impact of Pin1 catalytic activity on Notch proteins goes even beyond. This isomerase, in fact, also enhances p300-dependent N1-ICD acetylation (our unpublished observations), a modification shown to interfere with ubiquitination and degradation of both N1- and N4-ICD (Guarani *et al*, 2011; Ranganathan *et al*, 2011). Pin1 and Notch are even more intertwined than this: Pin1 favors Notch1 cleavage and activation by gamma-secretase (Rustighi *et al*, 2009) and, in turn, is a direct transcriptional target of both Notch1 and Notch4. Hence, deregulated signaling in cancer may fuel a Notch-Pin1 feed-forward loop, strongly contributing to tumor progression. Reinforcing this circuitry, the death associated protein kinase 1 (DAPK1), a tumor suppressor found hypermethylated in tumors and a crucial inhibitor of Pin1 isomerase activity (Lee *et al*, 2011), was shown to be repressed by N1-ICD (Li *et al*, 2008), delineating a possible scenario in which N1-ICD boosts not only expression but also activity of Pin1.

The interplay of Pin1 with the two Notch1 and Notch4 receptors implies its relevance in controlling Notch paralog-specific functions in the breast. In this respect, since Notch4 was suggested to be relevant in regulating the basal breast SC population, while Notch1 function could be more likely confined to luminal precursors both in the normal mammary gland as well as in breast cancer (Dontu *et al*, 2004; Bouras *et al*, 2008; Raouf *et al*, 2008; Harrison *et al*, 2010), our findings now would place Pin1 as a key regulator of stage-specific physiologic and pathologic Notch functions in this epithelial compartment.

Fbxw7α was previously shown to be involved in the maintenance of normal neural, colon and haematopoietic stem cells mostly through degradation of N1-ICD (Wang *et al*, 2011). The evidence that we present here indicates this tumor suppressor as a key negative force acting against expansion of the breast CSC compartment, causing degradation of N1- and N4-ICD and potently opposing breast CSC self-renewal and metastatic spread *in vivo*. However, more important to note in this context, is the antagonistic role of Pin1, which, despite Fbxw7α expression, could revert these effects by sustaining high levels of N1-ICD and N4-ICD, as depicted in the model of Fig 8F. Based on the above evidence, it is conceivable that aberrant expression or activity of Pin1 strongly reduce the selective pressure for activating mutations in NOTCH genes, or in genes encoding negative regulators of Notch signaling, such as Fbxw7α. This likely occurs in breast cancer, where *NOTCH1* or *FBXW7* manifest low mutational rates (Byrd *et al*, 2008; Mao *et al*, 2008; Ibusuki *et al*, 2011; Santarpia *et al*, 2012) and where, instead, high levels of Pin1 are frequent (Wulf *et al*, 2001; Girardini *et al*, 2011) and strongly correlate with activated N1-ICD. Our data on two different groups of breast cancer patients and from a meta-analysis, in which Notch signaling is constitutively activated despite high levels of Fbxw7, strongly support this hypothesis.

Besides its pivotal role in controlling transcription as a cofactor of CSL (CBF1/RBPJ-κ /Su(H) Lag1) family of DNA binding proteins, the ability of Notch to endow breast cancer cells with aggressive and stemness features results also from the interplay with several pathways, among them PI3K/Akt, Jak/STAT, NF-kappaB, ErbB2, Wnt, and HIF1 (Ranganathan *et al*, 2011). Strikingly, considering that most of them are also Pin1 substrates (Kim *et al*, 2008; Liou *et al*, 2011) this isomerase may contribute to increase Notch-induced oncogenesis also by amplifying the signalling emerging from these cross-talks. In this context, previously described connections of Notch family members with the p53 pathway (Beverly *et al*, 2005; Kim *et al*, 2007; Sun *et al*, 2010) deserve particular attention. In mammary stem cells wtp53 has a pivotal role in preventing symmetric division and uncontrolled expansion of stem cells (Cicalese *et al*, 2009; Insinga *et al*, 2013). Interestingly, this effect can be linked, at least in part, to the inhibition of the Notch pathway. Enhanced mammosphere formation observed by lack of p53, in fact, can be prevented by treatment with gamma-secretase inhibitors (Tao *et al*, 2011).

However not only the emerging roles of p53 in mammary stem cell biology may have relevant connections to our findings. Wild type p53 (wtp53), as well as its mutant counterparts (mutp53), in fact, are well known targets of Pin1 (Wulf *et al*, 2002; Zacchi *et al*, 2002; Zheng *et al*, 2002; Mantovani *et al*, 2007; Girardini *et al*, 2011; Grison *et al*, 2011; Sorrentino *et al*, 2013), raising the question on whether in the mammary stem cell compartment Pin1 could act in modulating p53 and Notch functions in concert, unveiling novel intersections between these two pathways. While in differentiated cells the activity of Pin1 on p53 has been well characterized in the context of genotoxic stress signaling acting on the *wildtype* protein, their interplay in the stem cell compartment remains to be elucidated. Therefore any consideration would be premature, even if the evidence that *Pin1*^*−/−*^ mice have reduced mammary stem cells (Fig 1C), in contrast with what has been observed in the *TP53*^*−/−*^ genotype (Cicalese *et al*, 2009; Tao *et al*, 2011), allows to speculate a more complex scenario for a wtp53-Pin1 interplay in these cells.

On the other hand, also for oncogenic gain of function (GOF) mutant p53 growing evidence supports a role in promoting cellular reprogramming and EMT and inciting expansion of mammary epithelial stem cells giving rise to mammary tumors (Sarig *et al*, 2010; Chang *et al*, 2011; Dong *et al*, 2013; Lu *et al*, 2013). In this context, we have recently demonstrated a key role of Pin1 in full unleashing mutp53 GOF in breast cancer (Girardini *et al*, 2011). On these bases, it is conceivable that in CSCs lacking wtp53 or expressing oncogenic mutp53, increased levels of Pin1, due to a hyperactivated Notch pathway, foster stem cell traits by acting both on mutp53 oncogenic properties and on Notch itself. In support of this hypothesis, our analysis revealed that poorly differentiated grade 3 and *NDT* high-*PIN1* high breast cancers are enriched for the expression of stem cell and mutp53 signature genes (supplementary Table S3).

From a clinical perspective, Pin1 promises to be a critical target in aggressive breast cancers. Our data demonstrate, in fact, that Pin1 inhibition, alone or coupled together with chemotherapeutic agents, could provide the opportunity to hit CSCs, restore chemosensitivity and inhibit metastatic spread.

## Materials and Methods

### Cell lines and treatments

MDA-MB-231, SK-BR-3, BT-549, and SUM-159 are human breast carcinoma cells, MCF10A are human normal immortalized epithelial breast cells, HEK 293T is a human embryonic kidney cell line with SV40 large T, immortalized *Pin1*^*−/−*^ fibroblasts have been obtained by spontaneous immortalization from Mouse Embryo Fibroblasts of C57BL6/129Sv mixed background (Rustighi *et al*, 2009). COS-7 are monkey kidney cells immortalized with SV40 large T antigen. NOP6 is a mouse mammary tumor cell line (Yang *et al*, 2009). Primary breast cancer cells from patients were obtained by disaggregation of surgical samples/biopsies from the Oncology Department at IRCCS S. Maugeri Foundation in Pavia (Italy). Cell culture media, transfections, infections and treatments are detailed in supplementary Data S1.

### Isolation and purification of mammary epithelial cells

Mammary glands from 8 to 12-week-old virgin female mice were enzymatically digested and single cell suspensions of purified mammary epithelial cells were obtained, as described (Sleeman *et al*, 2006; Stingl *et al*, 2006). Briefly, Mammary glands from 8 to 12-week-old virgin female mice were digested for 1–2 h at 37°C in EpiCult-B medium (StemCell Technologies Inc, Vancouver, Canada) with 600 U/ml collagenase (Sigma-Aldrich, St. Louis, MO, USA) and 200 U/ml hyaluronidase (Sigma). After lysis of the red blood cells with NH4Cl, the remaining cells were washed with PBS/0.02% w/v EDTA to allow cell-cell contacts begin to break down. Cells were then dissociated with 2 ml trypsin 0.25%w/v, 0.2% w/v EDTA for 2 min by gentle pipetting, then incubated in 5 mg/ml Dispase II (Sigma) plus 1 μg/ml DNase I (Sigma) for 5 min followed by filtration through a 40 μM cell strainer (BD Falcon, San Jose, CA, USA). Mammary epithelial cells were then purified using the EasySep Mouse Mammary Stem Cell Enrichment Kit (StemCell Technologies Inc).

### Flow cytometric analyses and sorting

Mouse mammary epithelial lineage-depleted cells, pre-enriched using the EasySep Mouse Mammary Stem Cell Enrichment Kit (see above), were analysed by FACS and sorted with antibodies against CD49f and CD24. FACS analyses and sorting from mammospheres based on aldehyde dehydrogenase (Aldh) activity were performed using the Aldefluor kit (StemCell Technologies Inc) following the manufacturer's instructions and are detailed in Supporting Information. Sorting of the populations of interest was performed on ARIA II cell sorter (Beckton Dickinson) to near purity (85%).

### Mammosphere cultures

To obtain mammospheres, cells from monolayer cultures were enzymatically disaggregated (0.05% trypsin–EDTA, Gibco) to a single cell suspension, passed though a 40 μm cell strainer (BD Falcon), plated at clonogenic density (2500 cells/cm^2^), and grown in nonadherent culture conditions, as described (Dontu *et al*, 2003). In detail, cells were grown for 7–10 days in DMEM:F12 (1:1) supplemented with B27 (Invitrogen Corporation, Carlsbad, CA, USA), 20 ng/ml EGF (PROSPEC, East Brunswick, NJ, USA), 20 ng/ml bFGF (BD Biosciences, San Jose, CA, USA), 4 μg/ml heparin (StemCell Technologies Inc.), 0.5 μg/ml hydrocortisone (Sigma) and 5 μg/ml Insulin (Sigma) in low attachment 24 or 96 well plates (Coroning) in a humified incubator at 37°C, 5% CO_2_. Primary mammospheres (≥200 μm) were obtained, collected, counted and again enzymatically disaggregated as above to re-plate cells at clonogenic densities to obtain secondary mammospheres. The same procedure was applied starting from secondary mammospheres to proceed to tertiary and quaternary mammospheres. Percentages of mammosphere forming efficiencies (%MFE) were calculated as number of mammospheres divided by the plated cell number and multiplied by a hundred. Mammospheres were counted with a 20× objective on an Olympus CK30 microscope (Olympus Italia Srl, Milan, Italy).

### Quantitative Real Time PCR (qRT–PCR) analysis

Total RNA from cell lines and xenografts was extracted with QIAzol Lysis Reagent (Qiagen Srl-Italy, Milan, Italy). Total RNA from formalin-fixed, paraffin-embedded samples of breast cancer patients was extracted starting from 2 to 3 20 μm slices with the HighPure RNA paraffin Kit (Roche SpA, Monza, Italy) and cDNA was transcribed with QuantiTect (Qiagen) in accordance with the manufacturer's protocols, then amplified on a StepOne Plus cycler (Applied Biosystems, Life Technologies Europe BV, Monza, Italy), using SYBR Green Universal PCR Master Mix (Applied Biosystems). Histone H3 and GAPDH mRNA were used as internal controls.

### Western blot, *in vitro* binding, and immunoprecipitation

*In vitro* binding assays, immuno- and co-immunoprecipitations, Western blot and immunofluorescence analyses were performed by standard procedures, as described (Rustighi *et al*, 2009; Girardini *et al*, 2011), and are detailed in Supporting Information. Co-IP experiments were performed in presence of proteasome inhibitor to avoid misinterpretation of binding results due to protein degradation.

### Ubiquitination analysis (Ni-NTA pull-down)

COS-7 cells were transfected with indicated combinations of vectors expressing His-Ubiquitin, N1-ICD-myc, Flag-Fbxw7α and control- or Pin1 specific siRNA. At 48 h post-transfection, cells were treated for 6 h with proteasome inhibitor MG132 (Sigma) and ubiquitin-aldehyde (Sigma) to preserve ubiquitin conjugates, then cells were lysed in highly denaturing conditions with lysis buffer containing 8 M urea, 0.5% triton and 10 mM imidazole and incubated with Ni^2+^ nitrilotriacetic acid (Quiagen N.1018244) beads for 4 h at room temperature according to manufacturers' recomendations. Beads were washed three times with lysis buffer and then analyzed by Western blot.

### Immunohistochemical analyses

Immunohistochemical analyses of mammary tissues from *Pin1*^*+/+*^ and *Pin1*^*−/−*^ mice and paraffin embedded tissue sections from 43 grade I–III invasive ductal and 38 triple negative breast carcinomas were performed by standard procedures, as previously described (Girardini *et al*, 2011) and are explained in detail in the Supporting Information section. Representative bright field images were taken with a Leica DM4000B microscope (Leica Microsystems S.r.l., Milan, Italy). The mouse and human studies were approved respectively by the ethical Committee of the University of Trieste, Italy (1444DEL12), and by the Institutional Review Ethical Board at the Hospital of Prato, Istituto Toscano Tumori, Prato, Italy and at the IRCCS S. Maugeri Foundation in Pavia, Italy.

### *In vivo* tumor growth experiments

For *in vivo* studies 7 weeks old SCID female mice (Charles River Laboratories, Lecco, Italy) were used and housed in a specific pathogen-free (SPF) animal facility. Procedures involving animals and their care were in conformity with institutional guidelines (D.L. 116/92 and subsequent complementing circulars) and all experimental protocols were approved by the ethical Committee of the University of Padua (CEASA). For xenograft studies of breast cancer, 150 000 to 10^6^ cells of indicated MDA-MB-231 clones were resuspended in 100 μl of DMEM, injected into the mammary fat pad on both flanks, of previously anesthetized mice (1–3% isoflurane, Merial Italia S.p.A, Italy). Tumor growth at the injection site was monitored by repeated caliper measurements (Rustighi *et al*, 2009). Tumor volume was calculated using the formula: tumor volume (mm^3^) = D × d2/2, where D and d are the longest and the shortest diameters, respectively. After 30 days the animals were anesthetized and the primary tumors were extracted and directly frozen in liquid nitrogen to perform molecular analyses. For metastasis studies the animals were then sacrificed at day 49. At this time point the lymph nodes and the lungs were excised and paraffin-embedded and formalin-fixed (DIAPATH S.P.A., Bergamo, Italy) for hematoxylin-eosin staining. For limiting dilution experiments data summarizing the tumor formation were uploaded into the web-based ELDA (Extreme Limiting Dilution Analysis) statistical software at http://bioinf.wehi.edu.au/software/elda/index.html (Hu & Smyth, 2009), which uses the frequency of tumor positive and negative animals at each transplant dose to determine the frequency of self-renewing cells. For chemotherapy experiments, 1 week after implantation of the cells, when the tumors became visible, paclitaxel (Taxol, Bristol-Myers Squibb Italia, Italy) was injected intravenously once a week at a concentration of 20 mg/ml. The tumors were monitored by measuring with the caliper and after 3 weeks from cells injection, the mice were sacrificed and the primary tumors were extracted and collected in PBS. All *in vivo* tumor growth experiments were conducted according to the UK Coordinating Committee on Cancer Research (UKCCCR) guidelines 1989 for the welfare of animals in experimental neoplasia. During *in vivo* experiments, animals in all experimental groups were examined daily for a decrease in physical activity and other signs of disease; severely ill animals were euthanized.

### Statistical analyses

For comparison of mammosphere forming efficencies, mRNA expression levels, protein half-lives, Co-IP analyses, transfections, and growth curves the *P*-values were obtained by applying one-tailed, type 2 *t*-test (assuming equal variances) using Microsoft Excel.

### Breast cancer data collection and processing

Twenty-one datasets comprising microarray data of breast cancer samples and annotations on patients' clinical outcome were collected. All data were measured on Affymetrix arrays and have been downloaded from Gene Expression Omnibus (http://www.ncbi.nlm.nih.gov/geo) and ArrayExpress (http://www.ebi.ac.uk/arrayexpress/). The complete list of datasets is provided in supplementary Table S1. Prior to analysis, the datasets were reorganized eliminating duplicate samples and samples without outcome information and each original study was renamed after the medical center where patients were recruited. The original studies have been modified as described in detail in supplementary Data S1. Details on the procedure of Classification of Tumors into low or high *NDT* signature groups appear in the Supporting Information section.

### Survival analysis

To evaluate the prognostic value of the *NDT* signatures, we estimated, using the Kaplan–Meier method (Kalbfleisch and Prentice), the probabilities that patients would remain free of death (survival). To confirm these findings, the Kaplan–Meier curves were compared using the log-rank or Mantel–Haenszel test (Harrington and Fleming). *P*-values were calculated according to the standard normal asymptotic distribution. *NDT* and *PIN1* signature levels were obtained as previously described (Adorno *et al*, 2009). Survival analyses and Kaplan–Meier plots were obtained using R survival and survcomp packages. Kaplan–Meier curves have been compared using the log-rank test of the surv_test function (coin R package).
