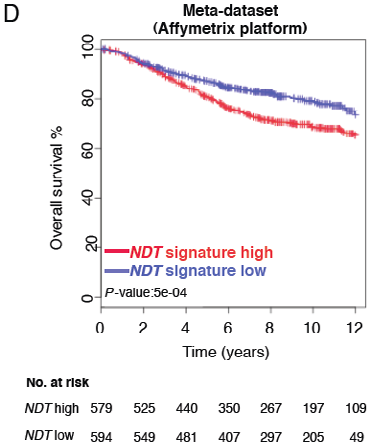# Prolyl-isomerase Pin1 controls normal and cancer stem cells of the breast

**DOI:** 10.1002/emmm.201470050

**Published:** 2014-05-06

**Authors:** Alessandra Rustighi, Alessandro Zannini, Luca Tiberi, Roberta Sommaggio, Silvano Piazza, Giovanni Sorrentino, Simona Nuzzo, Antonella Tuscano, Vincenzo Eterno, Federica Benvenuti, Libero Santarpia, Iannis Aifantis, Antonio Rosato, Silvio Bicciato, Alberto Zambelli, Giannino Del Sal



A typesetting error led to the deletion of the Y-axis label in Figure 1C. The label should have read “CD24,” and the correct figure is provided below. An author error resulted in the mislabeling of one of the data series in Supplementary Figure S9D. The blue label inside the Kaplan-Meier graphic should read “NDT signature low” instead of “NDT signature high.” The correct Supplementary Figure S9D is provided here. We apologize for any confusion these errors may have caused.

**Figure 1:**
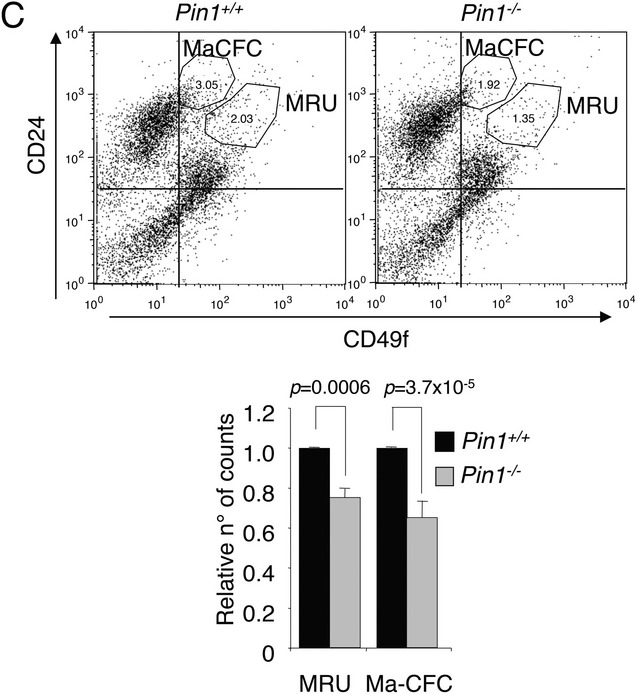


**Supplementary Figure S9:**