# Supplementary material for: Prolyl-isomerase Pin1 controls normal and cancer stem cells of the breast
Source: EMBO Mol Med. 2013 Dec 16;6(1):99–119. doi: 10.1002/emmm.201302909 (PMC3936488; doi:10.1002/emmm.201302909)
Supplement: Supplementary file 1 [file emmm0006-0099-sd1.pdf]

EMBO Molecular Medicine

Prolyl-isomerase Pin1 controls normal and cancer stem cells of the breast

Alessandra Rustighi, Alessandro Zannini, Luca Tiberi, Roberta Sommaggio, Silvano Piazza, Giovanni Sorrentino, Simona Nuzzo, Antonella Tuscano, Vincenzo Eterno, Federica Benvenuti, Libero Santarpia, Iannis Aifantis, Antonio Rosato, Silvio Bicciato, Alberto Zambelli, and Giannino Del Sal

Corresponding author: Giannino Del Sal, Laboratorio Nazionale CIB LNCIB and University of Trieste

Review timeline:

Submission date:	16 April 2013
Editorial Decision:	08 May 2013
Revision received:	13 September 2013
Editorial Decision:	30 September 2013
Revision received:	07 October 2013
Accepted:	08 October 2013

Transaction Report:

(Note: With the exception of the correction of typographical or spelling errors that could be a source of ambiguity, letters and reports are not edited. The original formatting of letters and referee reports may not be reflected in this compilation.)

Editor: Roberto Buccione

1st Editorial Decision 08 May 2013

Thank you for the submission of your manuscript to EMBO Molecular Medicine.

We experienced unusual difficulties in securing three appropriate reviewers in a timely manner. Since we cannot justify a further delay, I am making a decision based on the consistent evaluations of two Reviewers at this time.

You will see that while the Reviewers are generally supportive of you work, they express a number of concerns that prevent us from considering publication at this time. I will not dwell into much detail, as the evaluations are detailed and self-explanatory. I would like, however, to highlight a few main points.

Reviewer 1 notes that the functional analysis and clinical validation aspects of the study require further work. S/he mentions that in vivo experiments should be performed to strengthen the clinical implications of the cancersphere-based Pin1 knock-down/chemotherapy synergy. Reviewer 1 would also like you to evaluate the association between the Pin1/N1-ICD correlation and clinical outcome. Finally, s/he also mentions that further experimentation is required to consolidate the cancer stem frequency data based on tumour formation.

Reviewer 2 is concerned about the quantitative aspects of an IHC-based analysis and suggests that cells collected from the mice be grown and analysed biochemically. Similarly to Reviewer 1, s/he

also notes that the crucial data presented in Figure 7 should be significantly integrated and strengthened by probing serial sections and by providing a single-cell analysis for CSCs.

Considered all the above, while publication of the paper cannot be considered at this stage, we would be prepared to consider a revised submission, with the understanding that the Reviewers' concerns must be fully addressed, with additional experimental data where appropriate and that acceptance of the manuscript will entail a second round of review.

Please note that it is EMBO Molecular Medicine policy to allow a single round of revision only and that, therefore, acceptance or rejection of the manuscript will depend on the completeness of your responses included in the next, final version of the manuscript.

As you know, EMBO Molecular Medicine has a "scooping protection" policy, whereby similar findings that are published by others during review or revision are not a criterion for rejection. However, I do ask you to get in touch with us after three months if you have not completed your revision, to update us on the status. Please also contact us as soon as possible if similar work is published elsewhere.

I look forward to seeing a revised form of your manuscript as soon as possible.

***** Reviewer's comments *****

Referee #1 (Comments on Novelty/Model System):

This study provides intriguing evidence for a link between Pin1, Notch and Fbxw7alpha to stem cell properties in cancer promotion, metastatic spread and resistance to therapeutic efforts. Although the association between Pin1 and Fbxw7alpha as well as Fbxw7alpha and Notch have been shown in different contexts, this manuscript links this mechanism convincingly to breast cancer stem cells and unifies rather fragmented observations in a clinically relevant context. The biochemistry is convincing but revisions are needed on the functional analysis and clinical validation. Therefore, I cannot recommend the manuscript to be published in EMBO molecular medicine in the current form. However, I am generally positive about the study and with improvements I would recommend it as an article.

Referee #1 (Remarks):

This manuscript by Rustighi et al. follows and extends a previous study by the same group in which they described how the prolyl isomerase Pin1 enhances Notch1 transcriptional and tumorigenic activity (Rustighi et al Nat Cell Biol 2009). In the current manuscript, Rustighi and colleagues provide further molecular insights into the Pin1 mediated regulation of Notch signaling. While the previous study showed how Pin1 potentiates -secretase mediated cleavage of Notch1 receptor, the current study demonstrates that Pin1 protects Notch1 and Notch4 cytoplasmic domains (N1-ICD and N4-ICD) from proteasomal degradation in breast cancer cells. Pin1 action is exerted by inhibiting the interaction between N1-ICD and N4-ICD with the ubiquitin ligase Fbxw7alpha that normally restrains Notch signaling. The authors provide evidence for the importance of these molecular interactions to maintain stem cell properties in normal mammary epithelial cells and cancer cells promoting metastatic outgrowth and resistance to therapy. Overall, this is an interesting study that links previous molecular findings to mammary stem cells and a major clinical problem of cancer metastasis and resistance to chemotherapy. However, while the biochemical analyses are convincing, this reviewer believes the study needs to strengthen the functional evidence and clinical relevance. Details are provided below.

Specific points.

1.The authors show that Pin1 inhibition sensitizes cancer-spheroids to chemotherapy. In order to demonstrate that this is caused by Pin1 promotion of CSC phenotype, the investigators need to address the changes in CSC number (based on CD44+CD24- or Aldh1+) in Pin1 knockdowns in response to chemotherapy. Moreover, analysis of apoptosis would also be necessary to gain insights into the nature of Pin1 induced chemoresistance. This is particularly important since Fbwx7alpha

(the proposed mediator of Pin1 function) has been demonstrated to target the apoptosis inhibitor MCL1 for proteasomal degradation, thereby sensitizing cancer cells to chemotherapy (Wertz et al Nature 2011). Is MCL1 affected by Pin1 inhibition?

2.The synergy of Pin1 knockdown and chemotherapy to inhibit cultured cancer-spheres is convincingly demonstrated. However, a growing tumor in an animal provides importantly a more physiological context that needs to be addressed. In vivo experiments should be done to address synergy of Pin1 knockdown and chemotherapy.

3.The investigators show a reduced sphere formation and reduced Aldh1 positive population in MDA-MB-231. The choice of MDA-MB-231 cell line for sphere formation assay is surprising. This cell line is not known to form spheres efficiently but rather generates loosely associated aggregates. However, a widely used FACS profile for breast cancer stem cells is CD44+ CD24- and the MDA-MB-231 exhibits over 90% of its population within this profile. Does the CD44+ CD24- population require Pin1?

4.It is not clear to this reviewer how important the Notch pathway is as mediator of Pin1 promoted metastasis, since Fbwx7alpha regulates multiple targets.

5.The reduction of stem cell target genes upon Fbxw7alpha expression is fairly modest (Figure 6D). Very few genes reach 50% reduction. How can such a modest effect lead to a dramatic reduction in metastasis (Figure 6F). Please discuss. Are these genes really the mediators? Is MCL1 affected?

6.In Figure 7A an intriguing connection is observed between Pin1 and N1-ICD in patient samples. However, the practical significance of this correlation is missing. It would be important to address how Pin1 and N1-ICD correlation associates with clinical outcome?

7.In Table 1, the cancer stem cell frequency is determined based on tumor formation. The results are rather weak and statistics are missing. An increased number of mice and further limited dilution would strengthen this finding.

8.How specific to stem cell properties is the proposed phenotype of Pin1 mediated inhibition of Fbwx7alpha? Would this interaction not affect all cells?

9.Please discuss the difference between Pin-/- and Pin inhibition in an M2 sphere assay (Figure 1A and B). Pin-/- shows reduction in M2 spheres but Pin inhibition not.

10.In Figures 1D and 6D the error bars are missing for controls.

11.Figure 7B needs significant reconstruction. It is very confusing.

Referee #2 (Comments on Novelty/Model System):

In this study, Rustighi et al. describe an interesting new aspect of the oncogenic activity of PIN1 in breast cancer.
In details, the authors describe the ability of PIN1 to stabilize the intracellular NOTCH1 and 4 domains N1- and N4-ICD by protecting them from the pro-degradation activity of Fbxw7a. In the breast tumor cancer stem cells the sustained NOTCH pathway activity mediated by PIN1 promotes stem cells self-renewal and pro-survival stimuli under different genotoxic treatments.

The study is robust and the data presented support the conclusions.

Referee #2 (Remarks):

In this study, Rustighi et al. describe an interesting new aspect of the oncogenic activity of PIN1 in breast cancer.

In details, the authors describe the ability of PIN1 to stabilize the intracellular NOTCH1 and 4 domains N1- and N4-ICD by protecting them from the pro-degradation activity of Fbxw7a. In the breast tumor cancer stem cells the sustained NOTCH pathway activity mediated by PIN1 promotes stem cells self-renewal and pro-survival stimuli under different genotoxic treatments.

The study is robust and the data presented support the conclusions.

The following comments highlight specific concerns and suggestions regarding the manuscript that should be considered by the authors to further increase the quality and the robustness of their data.

1. Del Sal's group has already published several important papers regarding PIN1, mostly focused on the functional interaction between PIN1 and p53 either wild type or mutant. One of the last in particular describes the oncogenic function of PIN1 in breast cancer through the activation of mutant p53. Surprisingly, however, in this manuscript the possible implications of PIN1 activity on wild type p53 in the context of breast cancer stem cell self renewal and maintenance especially under specific genotoxic stress are never discussed (MDA-MB-231 for example are characterized by mutant p53). An integrated discussion regarding the PIN1-NOTCH1 and PIN1-P53 (wild type or mutant) pathways in CSCs biology is recommended.

2. PIN1 dependent role in breast CSCs maintenance through stabilization on N1-ICD and N4-ICD is shown as the potential of M2 to form tertiary and quaternary mammospheres. What it is no clear to this reviewer is the final biological outcome (i.e. differentiation, apoptosis, senescence) responsible for the exhaustion of the breast CSCs that experienced PIN1 loss-of-function (shPIN1 or PiB). Up-regulation of epithelial marker E-Cadherin and down-regulation of the mesenchimal markers Vimentin and Fibronectin is puzzling since it suggests a mesenchimal signature in breast tumor mammospheres that growth as tightly associated cells. The authors should better define this point.

3. Although the authors at the end exclude a direct competition between PIN1 and Fbxw7a for N1-ICD, the use of the T2512A N1-ICD mutant is useless to show that this site is fundamental for PIN1 dependent stabilization since the same site seems to be also important for the interaction between N1-ICD and Fbxw7a. Furthermore, although the half-life of the mutant form of N1-ICD is clearly longer than the wt, the levels of T2512A don't look to be higher than the wt at t=0 in Fig. 3D. The author should clarify if they transfected different amount of the 2 plasmids and if so, show the relative amount of the two transcripts by qPCR.

4. To correctly evaluate and compare the amount of a specific protein in different samples by IHC is very difficult. Yet, throughout the manuscript the authors appear to be able to collect primary mammary epithelial cells from both wt and Pin1-/- mice by FACS. The suggestion of this reviewer is to grow these cells, and compare by Western blot the amount of N1- and N4-ICDs.

5. The authors describe the correlation between PIN1, FBXW7a, and N1-ICD in human breast cancer patients in Figure 7. This pool of data is vital for all the manuscript, but the presentation of these results is at least unusual. The authors compare the IHC for N1-ICD (presented in a different manuscript submitted somewhere else) with the expression levels of PIN1 and FBXW7a mRNAs in 43 breast cancer specimens. Although the result looks encouraging, it is opinion of this reviewer that this analysis should be performed by IHC for PIN1, FBXW7a, and N1-ICD on serial sections of breast cancer specimens. Furthermore, since the authors are claiming that PIN1 dependent pro-NOTCH pathway activity is fundamental for the CSCs, a single cell evaluation by IHC becomes mandatory because these cells represent a small subgroup in the cancer tissue.

6. The authors suggest in the discussion that PIN1 inhibition might be a new important clinical approach for breast cancer therapy. Even if the data presented in this manuscript and in a previous one published by the same group (Girardini et al., 2011) robustly indicate PIN1 as a potential important new target in breast cancer, this group has also described PIN1 as a potent oncosuppressor through its positive activity on wild type p53. Rustighi et al., should further discuss this point, addressing for example patient stratification on the status of p53.

1st Revision - authors' response 13 September 2013

As requested by the reviewers, we have performed additional experiments to meet all the concerns. Details are listed below in point-by-point answers.

Referee #1 *(Comments on Novelty/Model System):*

This study provides intriguing evidence for a link between Pin1, Notch and Fbxw7alpha to stem cell properties in cancer promotion, metastatic spread and resistance to therapeutic efforts. Although the association between Pin1 and Fbxw7alpha as well as Fbxw7alpha and Notch have been shown in different contexts, this manuscript links this mechanism convincingly to breast cancer stem cells and unifies rather fragmented observations in a clinically relevant context. The biochemistry is convincing but revisions are needed on the functional analysis and clinical validation. Therefore, I cannot recommend the manuscript to be published in EMBO molecular medicine in the current form. However, I am generally positive about the study and with improvements I would recommend it as an article.

Referee #1 (Remarks):

This manuscript by Rustighi et al. follows and extends a previous study by the same group in which they described how the prolyl isomerase Pin1 enhances Notch1 transcriptional and tumorigenic activity (Rustighi et al Nat Cell Biol 2009). In the current manuscript, Rustighi and colleagues provide further molecular insights into the Pin1 mediated regulation of Notch signaling. While the previous study showed how Pin1 potentiates gamma-secretase mediated cleavage of Notch1 receptor, the current study demonstrates that Pin1 protects Notch1 and Notch4 cytoplasmic domains (N1-ICD and N4-ICD) from proteasomal degradation in breast cancer cells. Pin1 action is exerted by inhibiting the interaction between N1-ICD and N4-ICD with the ubiquitin ligase Fbxw7alpha that normally restrains Notch signaling. The authors provide evidence for the importance of these molecular interactions to maintain stem cell properties in normal mammary epithelial cells and cancer cells promoting metastati

outgrowth and resistance to therapy. Overall, this is an interesting study that links previous molecular findings to mammary stem cells and a major clinical problem of cancer metastasis and resistance to chemotherapy. However, while the biochemical analyses are convincing, this reviewer believes the study needs to strengthen the functional evidence and clinical relevance. Details are provided below.

Specific points.

1.The authors show that Pin1 inhibition sensitizes cancer-spheroids to chemotherapy. In order to demonstrate that this is caused by Pin1 promotion of CSC phenotype, the investigators need to address the changes in CSC number (based on CD44+CD24- or Aldh1+) in Pin1 knockdowns in response to chemotherapy.

Moreover, analysis of apoptosis would also be necessary to gain insights into the nature of Pin1 induced chemoresistance. This is particularly important since Fbwx7alpha (the proposed mediator of Pin1 function) has been demonstrated to target the apoptosis inhibitor MCL1 for proteasomal degradation, thereby sensitizing cancer cells to chemotherapy (Wertz et al Nature 2011). Is MCL1 affected by Pin1 inhibition?

To address this point, we performed an experiment *in vivo* by treating mice - harboring tumor xenografts of MDA-MB-231 cells expressing shCtrl or shPin1 - with Paclitaxel or vehicle (PBS) (new Fig. 4B). We analyzed both tumor growth and their content in Aldh positive cells As shown in new Figures 4B and 4C, Pin1 silencing not only retards tumor growth itself, but also blocks expansion of Aldh1-positive CSCs, therefore greatly improving the efficacy of Paclitaxel treatment. Now, the text has been changed accordingly.

Next, to gain insights into the nature of Pin1 induced chemoresistance we analyzed by Western blot

© EMBO

the cell lysates of the above tumors. As shown in Fig 4D we detected decreased N1-ICD expression in the tumours silenced for Pin1. Accordingly, a reversal of the EMT phenotype was observed, as shown by increased E-cadherin and decreased Slug and Vimentin protein levels (new Fig. 4D). In addition, Paclitaxel treatment induced caspase-3 cleavage that was strongly increased by Pin1 silencing, indicating that reduced levels of Pin1 synergize with Paclitaxel in inducing apoptosis and hence causing maximal tumour shrinkage.

The reviewer also asked if other Fbxw7a targets, such as Mcl-1, might be responsible for the observed chemosensitization by Pin1 downregulation. To answer this question, we performed Western blot analysis of the MDA-MB-231 xenografts of Fig. 4B treated with Paclitaxel or left untreated. This experiment indicated that Mcl-1 levels were indeed affected by Pin1 silencing. However, we did not observe further changes following Paclitaxel treatment, which did not allow to clearly assert the causal involvement of this protein in Pin1-dependent chemoresistance of these tumors. This information has now been included in the text (new Fig. 4D).

2.The synergy of Pin1 knockdown and chemotherapy to inhibit cultured cancer-spheres is convincingly demonstrated. However, a growing tumor in an animal provides importantly a more physiological context that needs to be addressed. In vivo experiments should be done to address synergy of Pin1 knockdown and chemotherapy.

We agree with this comment. In fact we assessed the synergy of Pin1 knockdown and chemotherapy in tumor xenografts as described above answering to the question n.1.

3. The investigators show a reduced sphere formation and reduced Aldh1 positive population in MDA-MB-231. The choice of MDA-MB-231 cell line for sphere formation assay is surprising. This cell line is not known to form spheres efficiently but rather generates loosely associated aggregates. However, a widely used FACS profile for breast cancer stem cells is CD44+ CD24- and the MDA-MB-231 exhibits over 90% of its population within this profile. Does the CD44+ CD24- population require Pin1?

We chose high Aldh1 activity as a marker for CSC in the MDA-MB-231 cell line, following the indications of Croker et al. (J. Cell. Mol. Med. 2009) that compared existing breast stem cell markers on several breast cancer cell lines, among them MDA-MB-231. Indeed, as also stated by the reviewer, over 90% of these cells express the CD44 stem cell marker when grown in adherence, therefore the stem cell population, constituted by a small proportion of cells, is more easily detected by using the Aldh1 profile rather than that of CD44high/CD24low.

As for the second concern of the reviewer, others groups successfully grew mammospheres from this cell line (Cordenonsi et al., Cell 2011; Prud'homme et al., PLoS ONE 2010; Grudzien et al., Anticancer Res. 2010; Phillips et al.; JNCI 2006). Notwithstanding, we were aware that MDA-MB-231 cells tend to form aggregates when grown in low-attachment conditions. Therefore, we used 0.1% methylcellulose to avoid aggregation between cells and could observe growth of mammospheres from single cells day by day. Once spheres had formed, their disaggregation - to obtain single cells for replanting or for FACS analyses - required a thorough trypsin treatment, indicating tight association in these conditions. We confirmed requirement of Pin1 for the self-renewal of MDA-MB-231 also by growing mammospheres from other breast/mammary cell lines, as shown in Figures 2A, 2B and Supporting Information 2A, right panel (BT-549, SUM-159, SK-BR-3) and from cells freshly derived from 2 primary breast cancers (new Supporting Information Fig. S2B). In addition, we further assessed requirement of Pin1 for other stem cell features by analyzing chemoresistance, tumor growth following limiting dilution and metastasis.

However, to address the reviewers' concerns we have engineered Pin1 overexpressing MCF10A cells and performed CD44/CD24 FACS analyses, that allowed us to demonstrate that high Pin1

levels cause an enrichment of the $CD44^{high}/24^{low}$ population. This result was now added as a new figure (Fig. 3D and Supporting Information Fig. S3C).

4.It is not clear to this reviewer how important the Notch pathway is as mediator of Pin1 promoted metastasis, since Fbwx7alpha regulates multiple targets.

Notch activation is fundamental for migration and metastasis of breast cancer cells, as demonstrated also by others (Leong et al., JEM 2007; McGowan et al., PNAS 2011; Xing et al., EMM 2012). Our results indicate that Pin1 is required for this aspect and the proposed mechanism is the protection of Notch1 and Notch4 from destruction by Fbxw7 ub-ligase. To strengthen this observation, we added another panel, Figure 8C (old Fig. 6D) showing that besides mRNA also the protein levels of several stemness, EMT (E-cadherin, Vimentin) and metastatic (Slug) targets controlled by the Notch pathway, that are downregulated by Fbxw7, are consistently recovered by Pin1 co-overexpression. Notably, these genes and their proteins are modulated as a group, indicating common regulation by Notch.

We are aware that Fbxw7 has several other targets perturbed by Pin1 that may concur to this phenotype and whose contribution cannot be completely excluded. Nevertheless the role of Pin1/Fbxw7/Notch axis in these phenotypes, as demonstrated by several approaches, appears to be of pivotal relevance.

5.The reduction of stem cell target genes upon Fbxw7alpha expression is fairly modest (Figure 6D). Very few genes reach 50% reduction. How can such a modest effect lead to a dramatic reduction in metastasis (Figure 6F). Please discuss. Are these genes really the mediators? Is MCL1 affected?

To demonstrate that the mRNA reduction of Notch target genes is sufficient to cause a phenotypic effect, we have performed Western blot analyses that allowed us to show modulation also at the protein level. For example, strong modulation of E-cadherin, Vimentin and Slug at the protein level (new Fig. 8C), is reminiscent of transition from a mesenchymal to an epithelial phenotype, as well as reduction of metastatic potential, as already shown by others (Guo et al., 2012; Leong et al., 2007). This might indicate that simultaneous reduction of several Notch target genes following Fbxw7alpha over-expression is enough to suppress aggressive features such as EMT, migration and invasion that are strictly connected to stemness and metastasis. In the same conditions, Mcl-1 protein levels did not change and therefore seems not to be involved.

6.In Figure 7A an intriguing connection is observed between Pin1 and N1-ICD in patient samples. However, the practical significance of this correlation is missing. It would be important to address how Pin1 and N1-ICD correlation associates with clinical outcome?

To address the question of how Pin1 and N1-ICD correlation in breast cancer patients associates with clinical outcome and to overcome the limitations of clinical information of this group of patients, we analysed our metadataset of Fig. 9B, (old Fig.7B, now reconstructed) for which clinical data were available. When all patients were analysed, Pin1 levels did not affect the clinical outcome neither in NDT high nor in NDT low expressing patients. Notably, when only grade 3 breast cancer patients were considered, high Pin1 levels were determinant for a worse outcome of patients with activated Notch1 signature (high NDT-high Pin1), but not for those with low NDT signature. These results are now included in the new Fig. 9D and Supporting Information Fig. S9E.

7. In Table 1, the cancer stem cell frequency is determined based on tumor formation. The results are rather weak and statistics are missing. An increased number of mice and further limited dilution would strengthen this finding.

Following this suggestion, we have now added new xenograft experiments with more dilutions and numbers of mice that also allowed a statistical analysis of the data. New Table 1 now displays 4 sequential dilutions, indicating that overexpression of Fbxw7α strongly decreases the number of CSCs frequency with respect to empty vector transduced cells. Conversely, co-overexpression of Pin1 significantly recovered CSCs frequency.

8. How specific to stem cell properties is the proposed phenotype of Pin1 mediated inhibition of Fbwx7alpha? Would this interaction not affect all cells?

To address this point we now performed a new set of experiments in different cell types and conditions that allowed us to compare the protein levels of Pin1 in stem cells to those in non stem cells:

1) we sorted mouse mammary stem cells (MRU) and compared them to the total cell population (new panel in old Fig. 1D);

2) we sorted Aldh1-positive and Aldh1-negative cells from MDA-MB-231 secondary mammospheres (new Fig. 3C, left panel);

3) we performed M2 cultures with primary cells isolated from breast cancers and compared them to the same cells cultured in adherence (new Fig.3C, right panel).

In all these settings we consistently detected higher Pin1 protein levels in the stem cell compartments, both normal and transformed, suggesting that high levels of Pin1 are specifically required in this pool of cells. Indeed, following ablation of Pin1, both genetically (*Pin1*$^{-/-}$, shRNA) and pharmacologically (Pin1 small molecule inhibitor PiB), we repeatedly detected impairment of stem cell features.

Concerning the question of the importance of Pin1 mediated inhibition of Fbxw7α in these cells, we have provided several indications that high Pin1 levels are important in inhibiting Fbxw7α function towards Notch in stem cells. Pin1, Fbxw7α and Notch interactions are supposed to occur also in non-stem cells, but in different biological processes, such as proliferation, apoptosis etc. However, since we found consistently higher levels of both Pin1 and N1-ICD in various stem cell compartments compared to their differentiated counterpart (Fig. 1D and new Fig. 3C), we may highlight them as stem cell factors.

9.Please discuss the difference between Pin-/- and Pin inhibition in an M2 sphere assay (Figure1A, B). Pin-/- shows reduction in M2 spheres but Pin inhibition not.

Treatment with the Pin1 inhibitor PiB was not as potent as a knock-out, since it was done starting from M1. However at late passages, under chronic prolonged exposure at the M3 level the decrease in mammosphere number following PiB treatment is strong and statistically significant.

10.In Figures 1D and 6D the error bars are missing for controls.

The error bar was added for Figure 1D, and Fig. 6D (now Fig. 8B) was represented similar to another qRT-PCR experiment in the manuscript and error bars were added.

11.Figure 7B needs significant reconstruction. It is very confusing.

We agree with the referee, and we now have represented the data as a heat map (new Fig. 9B).

Referee #2 (Comments on Novelty/Model System):

In this study, Rustighi et al. describe an interesting new aspect of the oncogenic activity of PIN1 in breast cancer.

In details, the authors describe the ability of PIN1 to stabilize the intracellular NOTCH1 and 4 domains N1- and N4-ICD by protecting them from the pro-degradation activity of Fbxw7a. In the breast tumor cancer stem cells the sustained NOTCH pathway activity mediated by PIN1 promotes stem cells self-renewal and pro-survival stimuli under different genotoxic treatments.

The study is robust and the data presented support the conclusions. Referee #2 (Remarks):

In this study, Rustighi et al. describe an interesting new aspect of the oncogenic activity of PIN1 in breast cancer.

In details, the authors describe the ability of PIN1 to stabilize the intracellular NOTCH1 and 4 domains N1- and N4-ICD by protecting them from the pro-degradation activity of Fbxw7a. In the breast tumor cancer stem cells the sustained NOTCH pathway activity mediated by PIN1 promotes stem cells self-renewal and pro-survival stimuli under different genotoxic treatments.

The study is robust and the data presented support the conclusions.

The following comments highlight specific concerns and suggestions regarding the manuscript that should be considered by the authors to further increase the quality and the robustness of their data.

1. Del Sal's group has already published several important papers regarding PIN1, mostly focused on the functional interaction between PIN1 and p53 either wild type or mutant. One of the last in particular describes the oncogenic function of PIN1 in breast cancer through the activation of mutant p53. Surprisingly, however, in this manuscript the possible implications of PIN1 activity on wild type p53 in the context of breast cancer stem cell self renewal and maintenance especially under specific genotoxic stress are never discussed (MDA-MB-231 for example are characterized by mutant p53). An integrated discussion regarding the PIN1-NOTCH1 and PIN1-P53 (wild type or mutant) pathways in CSCs biology is recommended.

We agree with the reviewer that the interplay with the p53 pathway deserves a discussion. Infact, in mammary stem cells wtp53 has a pivotal role in preventing symmetric division and uncontrolled expansion of stem cells (Cicalese 2009; Insigna et al., 2013). Interestingly, expansion of mammospheres caused by lack of *p53* was reduced by treatment with a gamma-secretase inhibitor, suggesting that basal levels of wtp53 restrict mammary stem/progenitor cells through inhibition of Notch (Tao et al., 2011). As pointed out by the reviewer, wtp53 is also a relevant target of Pin1, which is critical under genotoxic stress conditions to its pro-apoptoctic functions (Zacchi et al. 2002; Wulf et al., 2002; Mantovani et al., 2007; Grison et al., 2011; Sorrentino et al., 2013), thus raising the question whether Pin1 could also play a role in modulating wtp53 functions in the stem cell compartment. We can not exclude the possibility that Pin1 could also modulate some wtp53 functions in this context, however the evidence that *Pin1*$^{-/-}$ mice have a reduced mammary stem cell compartment (Fig. 1C), as opposed to the *TP53*$^{-/-}$ that show instead their expansion, suggests that action of Pin1 on wtp53 in this case could be different from the one described in more differentiated cells. In addition the role of Pin1 could be relevant, in the absence of p53, when expansion of stem cells requires full activation of Notch signaling for which Pin1 is essential.

A different scenario could be hypothesized for oncogenic missense p53 mutants (gain-of-function mutants). Several evidences demonstrated that mutp53 proteins are able promote cellular reprogramming, EMT and to expand mammary epithelial stem cells giving rise to mammary tumors

in transgenic mouse models (Sarig et al., 2010; Chang et al., 2011; Dong et al., 2013; Lu et al. 2013). We have recently shown that Pin1 binds to mutp53 and is a critical determinant of its oncogenic functions in breast cancer (Girardini et al., 2011). On this bases it is conceivable that in cancer stem cells expressing oncogenic p53 mutants, their expansion could be fostered by Pin1 acting simultaneously on both mutp53 and Notch axes. Further studies could clarify in this context the cross talk between these pathways, however at the current stage a deep investigation of this issue goes beyond the scope of the present manuscript).

2. PIN1 dependent role in breast CSCs maintenance through stabilization on N1-ICD and N4-ICD is shown as the potential of M2 to form tertiary and quaternary mammospheres. What it is no clear to this reviewer is the final biological outcome (i.e. differentiation, apoptosis, senescence) responsible for the exhaustion of the breast CSCs that experienced PIN1 loss-of-function (shPIN1 or PiB). Up-regulation of epithelial marker E-Cadherin and down-regulation of the mesenchimal markers Vimentin and Fibronectin is puzzling since it suggests a mesenchimal signature in breast tumor mammospheres that growth as tightly associated cells. The authors should better define this point.

About the reviewers' first concern regarding the final biological outcome of CSCs following Pin1 downregulation, we have now analysed by Western blot the cell lysates from MDA-MB-231 M4. These experiments demonstrated at the protein level that Pin1 silenced MDA-MB-231 M4 have the epithelial markers upregulated while the mesenchymal markers are downregulated. These experiments now clearly indicate that following Pin1 shRNA cells move towards a more differentiated epithelial cell type, a process that may account for the loss of replicative potential. This result has now been added in Fig. 2C.

About the second concern regarding the mesenchymal signature in mammospheres, it has been demonstrated that the epithelial to mesenchymal transition (EMT) is instrumental for generating stem cells by reprogramming an epithelial/differentiated cell towards a mesenchymal/stem phenotype (Mani et al. 2008, Guo et al., 2012, Chaffer et al., 2013). Hence, by definition, a breast stem cell looses epithelial features while it acquires mesenchymal properties, and loss of E-cadherin is sufficient to induce stemness and mammosphere formation in HMLE cells (Gupta et al., 2009). Therefore, although we don't know the molecular bases of the association between cells in the mammospheres, these can form also in absence or with low levels of E-cadherin, as observed for MDA-MB-231 cells.

Notwithstanding, we agree with this reviewer that the MDA-MB-231 cells are not a suitable model to determine EMT or the inverse process of differentiation towards an epithelial cell type. We therefore transduced MCF10A mammary epithelial cells with pLPC-HA-Pin1 and made several observations: a) The levels of cleaved N1-ICD were stabilized following Pin1 overexpression; b) Pin1 overexpression induced a clear EMT of these cells when grown in in adherence (2D); c) Pin1 overexpressing cells successfully formed secondary mammospheres while empty vector transduced cells could not; d) Inhibition of Notch activation by DAPT clearly abolished EMT and mammosphere growth. All these results are now shown as a new Figure (new Fig. 3D).

3. Although the authors at the end exclude a direct competition between PIN1 and Fbxw7a for N1-ICD, the use of the T2512A N1-ICD mutant is useless to show that this site is fundamental for PIN1 dependent stabilization since the same site seems to be also important for the interaction between N1-ICD and Fbxw7a. Furthermore, although the half-life of the mutant form of N1-ICD is clearly longer than the wt, the levels of T2512A don't look to be higher than the wt at t=0 in Fig. 3D. The author should clarify if they transfected different amount of the 2 plasmids and if so, show the relative amount of the two transcripts by qPCR.

It might seem useless to analyze the T2512A mutant, since it is more stable than N1-ICD due to abrogation of the Fbxw7α recognition site. However, since Pin1 binds also the central domain of N1-ICD (S2122, S2135 and T2137), as shown in Rustighi et al., 2009, with this experiment we tried

to unveil effects on protein half-life independent of T2512A, that we were able to exclude with this experiment.

As to the second concern, we on purpose calibrated the amount of transfected DNA to reach equal levels of overexpressed N1-ICD and N1-ICD-T2512A in order to better visualize the difference in protein decay during the CHX chase. This Figure has now been moved to the Supporting Information as Fig. S5G, due to space constraints.

4. To correctly evaluate and compare the amount of a specific protein in different samples by IHC is very difficult. Yet, throughout the manuscript the authors appear to be able to collect primary mammary epithelial cells from both wt and Pin1-/- mice by FACS. The suggestion of this reviewer is to grow these cells, and compare by Western blot the amount of N1- and N4-ICDs.

As suggested by the reviewer, we have extracted primary mammary epithelial cells (MECs) from *Pin1*$^{+/+}$ and *Pin1*$^{-/-}$ mammary glands and analysed N1- and N4-ICD levels by Western blot analysis, confirming the notion that presence of Pin1 is required for sustained Notch1 and Notch4 pathway activity *in vivo*. This result is now included as a new Figure (new Fig. 5D), while IHC images have been moved to Supporting Information Fig. S5N and the text was modified accordingly.

5. The authors describe the correlation between PIN1, FBXW7a, and N1-ICD in human breast cancer patients in Figure 7. This pool of data is vital for all the manuscript, but the presentation of these results is at least unusual. The authors compare the IHC for N1-ICD (presented in a different manuscript submitted somewhere else) with the expression levels of PIN1 and FBXW7a mRNAs in 43 breast cancer specimens. Although the result looks encouraging, it is opinion of this reviewer that this analysis should be performed by IHC for PIN1, FBXW7a, and N1-ICD on serial sections of breast cancer specimens.

To address this question, we have modified the original Figure 7 (now Fig. 9). Old Fig. 7A was moved to the Supporting Information Section as Fig. S9A and was replaced by a new analysis as follows: thanks to an available group of tissues from patients with triple negative breast cancer (TNBC) subtype (from Dr. Zambelli, FSM Pavia, Italy) we could analyse by IHC the expression of Pin1, Fbxw7α, and N1-ICD on serial sections. As shown in the new Fig. 9A and Supporting Information Fig. S9C we found that in this group there is a substantial number of tumours (72.7%) where high levels of Fbxw7 were found co-expressed with its target N1-ICD in an activated form. Interestingly, 93.8% of these patients displayed also high levels of Pin1 that might be responsible for the simultaneous presence of high N1-ICD and its ub-ligase, in agreement with the our observations in the previous version.

Furthermore, since the authors are claiming that PIN1 dependent pro-NOTCH pathway activity is fundamental for the CSCs, a single cell evaluation by IHC becomes mandatory because these cells represent a small subgroup in the cancer tissue.

As suggested by the reviewer we performed IHC analysis on serial sections of breast cancer samples to verify a co-localization between high Pin1 and high Aldh expressing cells. We found that Aldh positive cells express Pin1 but it was also expressed in Aldh negative cells (Supporting information Fig. S3B). Although being regarded as stem cell factors, many proteins are not exclusive of these type of cells, but are expressed also in more differentiated cells (e.g. Slug, Guo et al., 2012; p63, Yalcin-Ozuysal et al, 2010). Therefore, to better address if high levels of Pin1 are required for the stem/Aldh+ population, we either enriched or isolated (by sorting) the stem cell population and performed qRT-PCR and Western blot analyses to quantitatively determine that Pin1 is up-regulated in the stem cell compartment with respect to the more differentiated counterpart, as it has been done also for other stem cell factors that are ubiquitously expressed in epithelial breast cells.

In detail, we now performed a new set of experiments in different cell types and conditions that allowed us to compare stem cells to non stem cells:

1) we sorted mouse mammary stem cells (MRU) and compared them to the total cell population (new panel in old Fig. 1D);

2) we sorted Aldh1-positive and Aldh1-negative cells from MDA-MB-231 secondary mammospheres (new Fig. 3C, left panel);

3) we performed M2 culture with primary cells from breast cancer patients and compared them to the same cells cultured in adherence (new Fig.3C, right panel).

In all these settings we consistently detected higher Pin1 protein levels in the stem cell compartments, both normal and transformed, suggesting that high levels of Pin1 are specifically required in this pool of cells. Indeed, following ablation of Pin1, both genetically (*Pin1*$^{-/-}$, shRNA) and pharmacologically (Pin1 small molecule inhibitor PiB), we repeatedly detected impairment of stem cell features.

6. The authors suggest in the discussion that PIN1 inhibition might be a new important clinical approach for breast cancer therapy. Even if the data presented in this manuscript and in a previous one published by the same group (Girardini et al., 2011) robustly indicate PIN1 as a potential important new target in breast cancer, this group has also described PIN1 as a potent oncosuppressor through its positive activity on wild type p53. Rustighi et al., should further discuss this point, addressing for example patient stratification on the status of p53.

The point made by the reviewer relates on whether Pin1 functions as a tumour suppressor, being able to regulate wtp53 functions -based on previous evidences (Zacchi et al., 2002; Zheng et al., 2002; Wulf et al., 2002; Mantovani et al., 2007) - or as an oncogene, since Pin1 has been also shown to promote mutp53 oncogenic activities in breast cancer (Girardini et al., 2011). From these apparently contradictory evidences an ambivalent role of Pin1 emerges, either as suppressor or promoter of tumourigenesis. This is intrinsic to the enzymatic nature of Pin1, the number of proteins that it controls, their availability, and to its function being strongly influenced by the cellular context (as also reported for several other proteins, e.g. TGF-b, Adorno et al., 2009). Considering the action of Pin1 on wtp53, our results suggest that in cells expressing wtp53 and following DNA damaging agents, Pin1 is important for a proficient p53-dependent apoptotic response that involves non transcriptional (mitochondrial) and transcriptional activities of p53. In contrast, in cells bearing mutp53, Pin1 can bind to mutp53 and enhance its pro-aggressive functions. Therefore Pin1 acts both as a fine tuner of wtp53 oncosuppressive activities and as an amplifier of mutp53 oncogenic functions. The final outcome of its activities depends on the specific cellular context. Based on these evidences, the choice of an anti-Pin1 therapy must consider p53 status of the patients. Combined or sequential therapies directed against stem or bulk cells of the tumor must also be taken into consideration. All these considerations have now been discussed in the new version.

References

Adorno, M, Cordenonsi, M, Montagner, M, Dupont, S, Wong, C, Hann, B, Solari, A, Bobisse, S, Rondina, MB, Guzzardo, V, et al (2009) A Mutant-p53/Smad complex opposes p63 to empower TGFbeta-induced metastasis. Cell 137: 87-98

Croker, AK, Goodale, D, Chu, J, Postenka, C, Hedley, BD, Hess, DA, Allan, AL (2009) High aldehyde dehydrogenase and expression of cancer stem cell markers selects for breast cancer cells with enhanced malignant and metastatic ability. J Cell Mol Med 13: 2236-2252

Cicalese, A, Bonizzi, G, Pasi, CE, Faretta, M, Ronzoni, S, Giulini, B, Brisken, C, Minucci, S, Di Fiore, PP, Pelicci, PG (2009) The tumour suppressor p53 regulates polarity of self-renewing divisions in mammary stem cells. Cell 138: 1083-1095

Chaffer, CL, Marjanovic, ND, Lee, T, Bell, G, Kleer, CG, Reinhardt, F, D'Alessio, AC, Young, RA, Weinberg RA (2013) Poised Chromatin at the ZEB1 Promoter Enables Breast Cancer Cell Plasticity and Enhances Tumorigenicity. Cell 154: 61-74.

Chang, CJ, Chao, CH, Xia, W, Yang, JY, Xiong, Y, Li, CW, Yu, WH, Rehman, SK, Hsu, JL, Lee HH, et al (2011) p53 regulates epithelial-mesenchymal transition and stem cell properties through modulating miRNAs. Nat Cell Biol 13: 317-323

Cordenonsi, M, Zanconato, F, Azzolin, L, Forcato, M, Rosato, A, Frasson, C, Inui M, Montagner M, Parenti AR, Poletti A, et al (2011) The Hippo transducer TAZ confers cancer stem cell-related traits on breast cancer cells. Cell. 147: 759-72

Dong, P, Karaayvaz, M, Jia, N, Kaneuchi, M, Hamada, J, Watari, H, Sudo, S, Ju, J, and Sakuragi, N (2013) Mutant p53 gain-of-function induces epithelial-mesenchymal transition through modulation of the miR-130b-ZEB1 axis. Oncogene 32, 3286-95

Girardini, JE, Napoli, M, Piazza, S, Rustighi, A, Marotta, C, Radaelli, E, Capaci, V, Jordan, L, Quinlan, P, Thompson, A, et al (2011) A Pin1/mutant p53 axis promotes aggressiveness in breast cancer. Cancer Cell 12: 79-91

Grison, A, Mantovani, F, Comel, A, Agostoni, E, Gustincich, S, Persichetti, F, Del Sal, G. (2011) Ser46 phosphorylation and prolyl-isomerase Pin1-mediated isomerization of p53 are key events in p53-dependent apoptosis induced by mutant huntingtin. Proc Natl Acad Sci U S A 108: 17979-17984

Grudzien, P, Lo, S, Albain, KS, Robinson, P, Rajan, P, Strack, PR, Golde, TE, Miele, L, Foreman, K,E (2010) Inhibition of Notch signaling reduces the stem-like population of breast cancer cells and prevents mammosphere formation. Anticancer Res 30: 3853-3867

Guo, W, Keckesova, Z, Donaher, JL, Shibue, T, Tischler, V, Reinhardt, F, Itzkovitz, S, Noske, A, Zürrer-Härdi, U, Bell, RA, et al (2012) Slug and Sox9 cooperatively determine the mammary stem cell state. Cell 148: 1015-28

Gupta, PB, Onder, TT, Jiang, G, Tao, K, Kuperwasser, C, Weinberg, RA, Lander, ES (2009) Identification of selective inhibitors of cancer stem cells by high-throughput screening. Cell 138: 645-59

Insinga, A, Cicalese, A, Faretta, M, Gallo, B, Albano, L, Ronzoni, S, Furia, L, Viale, A, Pelicci, PG. (2013) DNA damage in stem cells activates p21, inhibits p53, and induces symmetric self-renewing divisions. Proc Natl Acad Sci U S A 110: 3931-3936

Leong, KG, Niessen, K, Kulic, I, Raouf, A, Eaves, C, Pollet, I, Karsan, A (2007) Jagged1-mediated Notch activation induces epithelial-to-mesenchymal transition through Slug-induced repression of E-cadherin. J Exp Med 204: 2935-2948

Lu, X, Liu, DP, Xu, Y (2013) The gain of function of p53 cancer mutant in promoting mammary tumorigenesis. Oncogene. 2013 32: 2900-2906

Mani, SA, Guo, W, Liao, MJ, Eaton, EN, Ayyanan, A, Zhou, AY, Brooks, M, Reinhard, F, Zhang, CC, Shipitsin, M, et al (2008) The epithelial-mesenchymal transition generates cells with properties of stem cells. Cell 133: 704-715

Mantovani, F, Tocco, F, Girardini, J, Smith, P, Gasco, M, Lu, X, Crook, T, Del Sal, G. (2007) The prolyl isomerase Pin1 orchestrates p53 acetylation and dissociation from the apoptosis inhibitor iASPP. Nat Struct Mol Biol 14: 912-920

McGowan, PM, Simedrea, C, Ribot, EJ, Foster, PJ, Palmieri, D, Steeg, PS, Allan, AL, Chambers, AF (2011) Notch1 Inhibition Alters the CD44hi/CD24lo Population and Reduces the Formation of Brain Metastases from Breast Cancer. Mol Cancer Res 9: 834-844

Prud'homme, GJ, Glinka, Y, Toulina, A, Ace, O, Subramaniam, V, Jothy S (2010) Breast cancer stem-like cells are inhibited by a non-toxic aryl hydrocarbon receptor agonist. PLoS One 5: e13831.

Phillips, TM, McBride, WH, Pajonk, F (2006) The Response of CD24−/low/CD44+ Breast Cancer–

Initiating Cells to Radiation. Journal of the National Cancer Institute 98: 1777-1785

Rustighi, A, Tiberi, L, Soldano, A, Napoli, M, Nuciforo, P, Rosato, A, Kaplan, F, Capobianco, A, Pece, S, Di Fiore, PP, Del Sal G (2009) The prolyl-isomerase Pin1 is a Notch1 target that enhances Notch1 activation in cancer. Nat Cell Biol 11: 133-142

Sarig, R, Rivlin, N, Brosh, R, Bornstein, C, Kamer, I, Ezra, O, Molchadsky, A, Goldfinger, N, Brenner, O, and Rotter, V (2010) Mutant p53 facilitates somatic cell reprogramming and augments the malignant potential of reprogrammed cells. J Exp Med. 207, 2127-2140

Sorrentino, G, Mioni, M, Giorgi, C, Ruggeri, N, Pinton, P, Moll, U, Mantovani, F, Del Sal, G. (2013) The prolyl-isomerase Pin1 activates the mitochondrial death program of p53. Cell Death Differ. 20: 198-208

Wulf, GM, Liou, YC, Ryo, A, Lee, SW, Lu, KP (2002) Role of Pin1 in the regulation of p53 stability and p21 transactivation, and cell cycle checkpoints in response to DNA damage. J Biol Chem 277: 47976-47979

Xing, F, Kobayashi, A, Okuda, H, Watabe, M, Pai, SK, Pandey, P.R., Hirota, S, Wilber, A., Mo, YY, Moore, BE et al (2012) Reactive astrocytes promote the metastatic growth of breast cancer stem-like cells by activating Notch signaling in brain. EMBO Mol Med 5: 384-396

Yalcin-Ozuysal, O, Fiche, M, Guitierrez, M, Wagner, KU, Raffoul, W, Brisken, C (2010) Antagonistic roles of Notch and p63 in controlling mammary epithelial cell fates. Cell Death Differ 17: 1600-1612

Zacchi, P, Gostissa, M, Uchida, T, Salvagno, C, Avolio, F, Volinia, S, Ronai, Z, Blandino, G, Schneider, C, Del Sal, G. (2002) The prolyl isomerase Pin1 reveals a mechanism to control p53 functions after genotoxic insults. Nature 419: 853-857

Zheng, H, You, H, Zhou, XZ, Murray, SA, Uchida, T, Wulf, G, Gu, L, Tang, X, Lu, KP, Xiao, ZX (2002) The prolyl isomerase Pin1 is a regulator of p53 in genotoxic response. Nature 419:849-853.

2nd Editorial Decision 30 September 2013

Thank you for the submission of your revised manuscript to EMBO Molecular Medicine. We have now heard back from the two Reviewers whom we asked to re-evaluate your manuscript.

You will see that both Reviewers are now supportive. Reviewer 1, however, has remaining issues that require your action before we can accept your manuscript for publication.

Reviewer 1 would like you to provide discussion/explanations to reconcile the potential discrepancies and or unclear issues between your experimental findings and the observations on human tumours.

Please fully address Reviewer 1's remaining concerns. Provided these issues are fully addressed, I am prepared to make an editorial decision on your manuscript.

I look forward to receiving your re-revised manuscript as soon as possible and in any case within two weeks.

***** Reviewer's comments *****

Referee #1 (Comments on Novelty/Model System):

The manuscript has been strengthened significantly by improving functional analysis and including

additional experiments in support of the described findings. Moreover, clinical evidence has been extended, although some of the added results need clarification and further discussion (see below).

Referee #1 (Remarks):

The authors have significantly improved and strengthened the study described in this revised manuscript. The concerns of this reviewer have principally been addressed. However, there are a couple of points that require further attention. In the recently added figures 9D and S9E, the authors show that high Pin1 expression predicts poor survival in Notch direct target gene signature (NDT) high tumors while this distinction is absent in NDT low tumors. These results need to be discussed further in the light of the important role for Notch presented in the manuscript where Notch receptors are significantly induced by Pim1 and suggested to be key mediators of Pim1 promoted cancer progression. The survival data in figures 9D and S9E suggest a clinical importance of a role for Pim1 that is independent of Pim1 induced Notch activity. This should be discussed and clarified. Moreover, the distinct association of Pim1 to poor prognosis in grade 3 breast cancer and not in lower grade breast cancer should be discussed. The explanation referring to poor differentiation of grade 3 tumors and Pim1 promotion of stem cell properties is rather ambiguous. Particularly as Pim1 is presented as a major regulator of stemness, even in normal mammary epithelial stem cells. When Pim1 is expressed in low grade breast cancer, should it not promote stemness and thereby predict poor survival? Or is there an explanation for distinct biological consequences of Pim1 in high grade versus low grade tumors. After addressing and clarifying these points sufficiently, this reviewer would support the publication of the manuscript.

Referee #2 (Comments on Novelty/Model System):

In their revision, the authors have addressed the main concerns of this reviewer, making the technical aspects of their manuscript more robust.

Referee #2 (Remarks):

In this study, Rustighi et al. describe the ability of PIN1 to promote the NOTCH pathway through the stabilization of N1- and N4-ICD, which it protects from the pro-degradation activity of Fbxw7a. In the process, PIN1 activity counteracts different genotoxic treatments by promoting the self-renewal and survival of breast tumor cancer stem cells.

The authors have convincingly addressed the major concerns of this reviewer, making the manuscript clearer and more compelling, and therefore publication can now be recommended.

2nd Revision - authors' response 07 October 2013

Answers to the comments of referee #1

In the recently added figures 9D and S9E, the authors show that high Pin1 expression predicts poor survival in Notch direct target gene signature (NDT) high tumors while this distinction is absent in NDT low tumors. These results need to be discussed further in the light of the important role for Notch presented in the manuscript where Notch receptors are significantly induced by Pim1 and suggested to be key mediators of Pim1 promoted cancer progression. The survival data in figures 9D and S9E suggest a clinical importance of a role for Pim1 that is independent of Pim1 induced Notch activity. This should be discussed and clarified.

We agree with the reviewer's concern that we now clarify.

High levels of Pin1 have been shown to act as amplifier of phosphorylation dependent signaling pathways. It is worth noting, that, due to its enzymatic nature, Pin1 overexpression alone is able to induce aggressive phenotypes, only in function of its substrates (Wulf et al., 2005), as well as to

predict patients' outcome in dependence of activated oncogenic pathways such as mutp53 (Girardini et al., 2011) or Notch1, as we show here.

The results of the K-M show that patients with high *NDT* signature have the worst prognosis when Pin1 levels are concurrently high, whereas in the absence of either the oncogenic pathway to be amplified (i.e. Notch pathway, Fig. S9E) or of the amplifier (Pin1) (Fig. 9D), the outcome is more favorable. These findings suggest that the influence of Pin1 on the Notch pathway is likely not limited to its direct action on Notch proteins. Indeed, among Notch targets several are also Pin1 substrates (e.g. cyclin D1, NF-κB, Survivin) (Wulf et al., 2005; Cheng et al., 2013) suggesting that, in addition to its role in sustaining Notch activity, Pin1 could promote breast cancer aggressiveness also by enhancing their action. These aspects have been introduced in the discussion (page 26).

Moreover, the distinct association of Pim1 to poor prognosis in grade 3 breast cancer and not in lower grade breast cancer should be discussed. The explanation referring to poor differentiation of grade 3 tumors and Pim1 promotion of stem cell properties is rather ambiguous. Particularly as Pim1 is presented as a major regulator of stemness, even in normal mammary epithelial stem cells. When Pim1 is expressed in low grade breast cancer, should it not promote stemness and thereby predict poor survival? Or is there an explanation for distinct biological consequences of Pim1 in high grade versus low grade tumors. After addressing and clarifying these points sufficiently, this reviewer would support the publication of the manuscript.

Pin1 is an ubiquitously expressed protein in both normal breast ductal cells and in breast cancer tissues. However its levels have been clearly demonstrated to correlate with tumour grade (Wulf et al., 2001). We have shown that the stem cell compartment, both normal and tumoral, expresses higher levels of Pin1 with respect to the total considered cell population and that normal mammary stem cells require those Pin1 levels for normal development. Even though Pin1 levels are high in normal stem cells, the correct balance of stem/progenitors and differentiated cells in this context is guaranteed by physiological mechanisms. In breast cancer, Pin1 is transcriptionally up-regulated by oncogenic activation of several pathways (Wulf et al., 2005; Rustighi et al., 2009). As a consequence, Pin1 becomes gradually overexpressed, displaying highest levels in high grade breast cancer, compared to normal tissue or low grade tumors (Wulf et al., 2001). Low grade tumors are by definition morphologically similar to normal tissue. Accordingly, Pece et al. (2010) have clearly shown that normal tissue and G1/low grade tumors have a comparable stem cell content and explain this observation assuming that surveillance mechanisms are still active in controlling aberrant expansion of CSCs (e.g. presence of wt-p53). Hence, like in normal tissue, an eventual Pin1-dependent stem cell expansion in G1 tumors is still kept under control.

A different scenario can be envisaged in G3/high grade tumors, where genetic/epigenetic modifications accumulated over time have promoted the functional loss of surveillance mechanisms (among them p53 mutations). In this situation high Pin1 levels are able to unleash an abnormal cancer stem cell expansion by acting on the Notch pathway and on its cross talks.

References

Cheng, CW, Chow, AK, Pang, R, Fok, EW, Kwong, YL, Tse, E. (2013) PIN1 inhibits apoptosis in hepatocellular carcinoma through modulation of the antiapoptotic function of survivin. Am J Pathol 182: 765-75

Pece, S, Tosoni, D, Confalonieri, S, Mazzarol, G, Vecchi, M, Ronzoni, S, Bernard, L, Viale, G, Pelicci, PG, Di Fiore, PP (2010) Biological and molecular heterogeneity of breast cancers correlates with their cancer stem cell content. Cell 140: 62-73

Rustighi, A, Tiberi, L, Soldano, A, Napoli, M, Nuciforo, P, Rosato, A, Kaplan, F, Capobianco, A, Pece, S, Di Fiore, PP, Del Sal G (2009) The prolyl-isomerase Pin1 is a Notch1 target that enhances Notch1 activation in cancer. Nat Cell Biol 11: 133-142

Wulf, GM, Ryo, A, Wulf, GG, Lee, SW, Niu, T, Petkova, V, Lu, KP. (2001) Pin1 is overexpressed in breast cancer and cooperates with Ras signaling in increasing the transcriptional activity of c-Jun towards cyclin D1. EMBO J 20: 3459-3472

Wulf, G, Finn, G, Suizu F, Lu, KP. (2005) Phosphorylation-specific prolyl isomerization: is there an underlying theme? Nat Cell Biol 7: 435-441
