# Supplementary material for: Prolyl-isomerase Pin1 controls normal and cancer stem cells of the breast
Source: EMBO Mol Med. 2013 Dec 16;6(1):99–119. doi: 10.1002/emmm.201302909 (PMC3936488; doi:10.1002/emmm.201302909)
Supplement: Supplementary file 2 [file emmm0006-0099-sd2.pdf]

# Supporting Information for

# Prolyl-isomerase Pin1 controls normal and cancer stem cells of the breast

Alessandra Rustighi, Alessandro Zannini, Luca Tiberi, Roberta Sommaggio, Silvano Piazza, Giovanni Sorrentino, Simona Nuzzo, Antonella Tuscano, Vincenzo Eterno, Federica Benvenuti, Libero Santarpia, Iannis Aifantis, Antonio Rosato, Silvio Bicciato, Alberto Zambelli, and Giannino Del Sal

This supporting data section is composed by results supporting the main findings in the manuscript (9 figures and figure legends, 3 Tables), supporting materials and methods and supporting references.

## Table of content


- **Supporting Information Fig. S1:** Molecular characterization of the sorted MRU and Ma-CFC subsets of mammary epithelial cells.
- **Supporting Information Fig. S2:** Pin1 controls human breast CSC self-renewal and replicative potential.
- **Supporting Information Fig. S3:** Pin1 regulates breast CSCs self-renewal by keeping high levels of N1-ICD.
- **Supporting Information Fig. S4:** Pin1 downmodulation sensitizes breast CSCs to chemotherapeutic treatment.
- **Supporting Information Fig. S5:** Pin1 hampers Fbxw7α-mediated degradation of N1- and N4-ICDs.
- **Supporting Information Fig. S6:** Pin1 uncouples N1-ICD and N4-ICD from Fbxw7α in a PP2A dependent manner.
- **Supporting Information Fig. S7:** Pin1 sustains Notch-dependent stem cell features despite Fbxw7α.
- **Supporting Information Fig. S8:** Pin1 sustains Notch-dependent stem cell and tumorigenic features despite Fbxw7α *in vivo*.
- **Supporting Information Fig. S9:** Association of *FBXW7* or *PIN1* expression in N1-ICD high and low breast cancers.
- **Supporting Information Table S1.** Breast cancer re-organized cohorts comprised in the meta-dataset analyzed in this study.
- **Supporting Information Table S2.** Notch-dependent Direct Target (NDT) gene signature.
- **Supporting Information Table S3.** Enrichment of gene signatures in the list of genes preferentially expressed in *NDT* signature HIGH/*PIN1* HIGH versus *NDT* signature HIGH/*PIN1* LOW tumors in the metadataset (GRADE 3).
- **Supporting Information Materials and Methods**
- **Supporting Information References**

**Supporting Information Figure S1.**

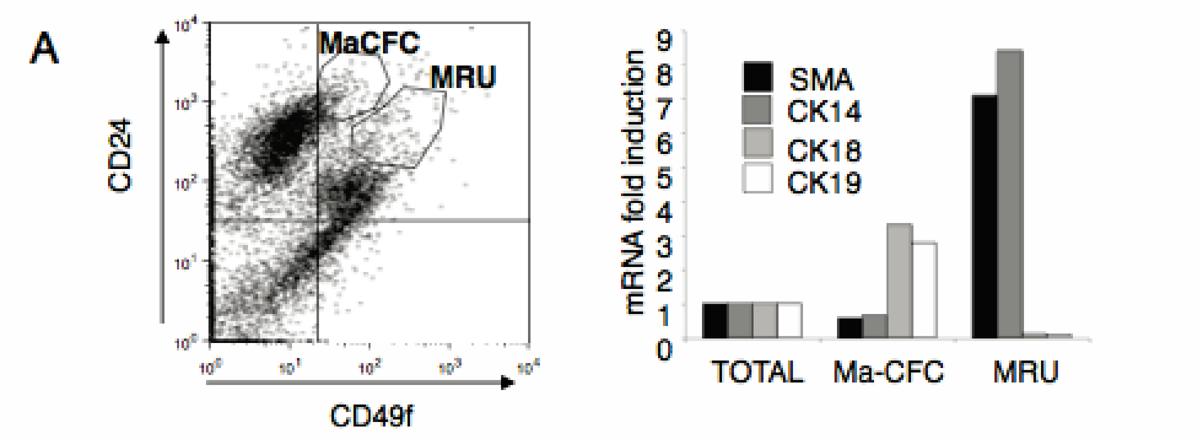


**Supporting Information Figure S1.** (**refers to Figures 1C and 1D**) **Molecular characterization of the sorted MRU and Ma-CFC subsets of mammary epithelial cells.** **(A)** Control qRT-PCR for myoepithelial (SMA, CK14) and luminal (CK18, CK19) markers of mRNA extracted from MRU and Ma-CFC (Stingl et al., 2006) sorted populations relative to the total population, shows accuracy of the FACS sorting procedure.

**Supporting Information Figure S2.**

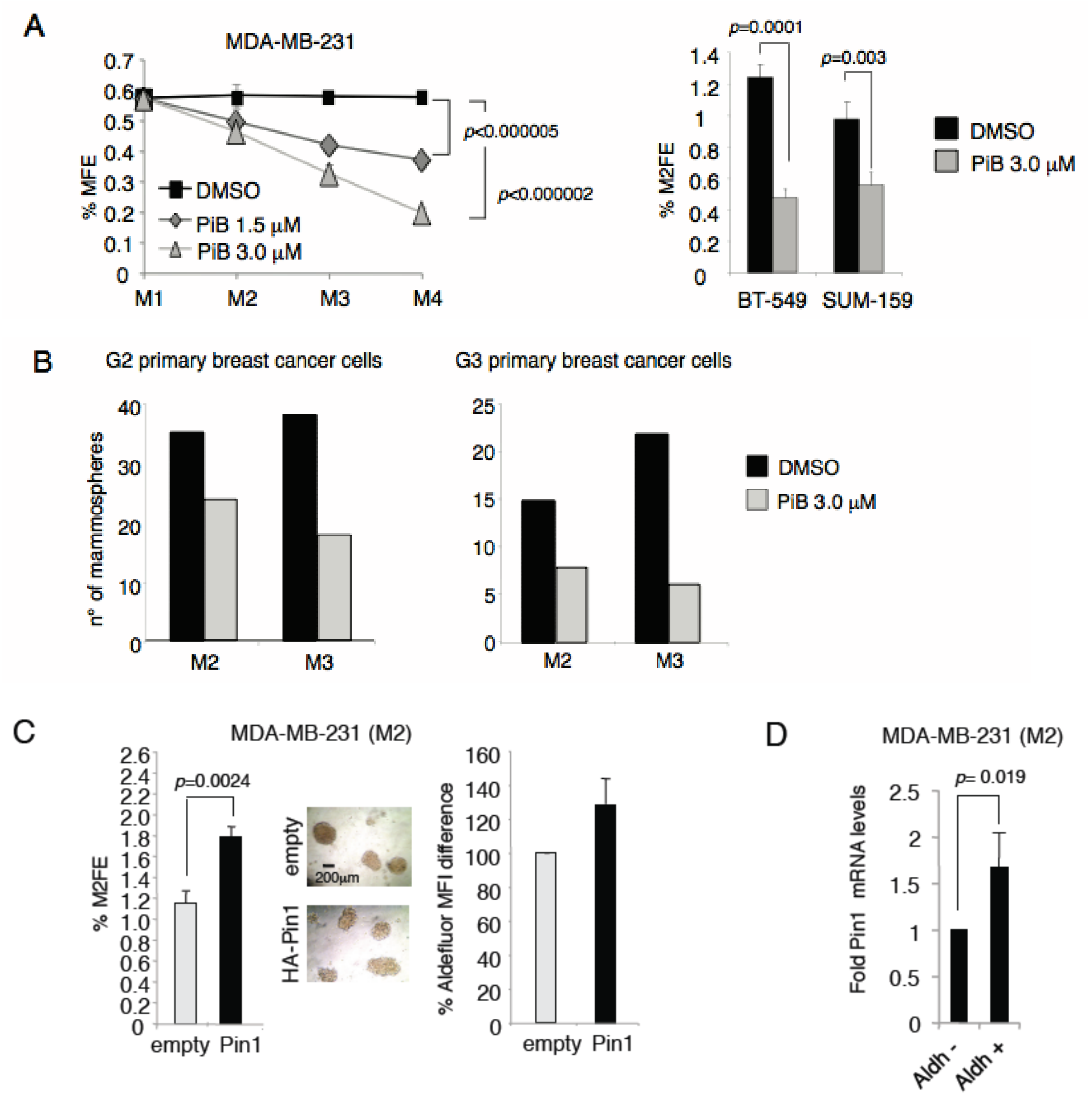


**Supporting Information Figure S2. (refers to Figure 2) Pin1 controls human breast CSC self-renewal and replicative potential.**

**(A)** Left: Serial replating of mammospheres (M1-M4) generated from MDA-MB-231 human breast cancer cells and treated with DMSO or PiB (1,5 μM and 3 μM). Mammosphere formation efficiency (%MFE) was calculated as percentage of mammospheres divided by the number of plated cells. Means, standard deviations and *p*-values are indicated (t-test, n=3, M4). Right: Percentage of M2FE of BT-549 and SUM-159 breast cancer cell lines treated with vehicle (DMSO) or PiB. Means, standard deviations and *p*-values are indicated (t-test, n=3). **(B)** Secondary and tertiary mammosphere formation efficiency of a grade 2 (G2) and grade 3 (G3) patient-derived breast cancer cells treated with vehicle (DMSO) or 3 μM PiB. **(C)** Left: Secondary mammosphere formation efficiency of MDA-MB-231 cells transduced with empty or HA-Pin1 expressing vectors. Representative microphotographs and scale bars are shown. Right: Histogram showing

percentage of Aldefluor median fluorescence intensity difference (MFI). **(D)** qRT-PCR of endogenous Pin1 mRNA in sorted MDA-MB-231 Aldh-positive compared to Aldh negative cells. Means, standard deviations and *p*-value are indicated (t-test, n=3, M2).

**Supporting Information Figure S3.**

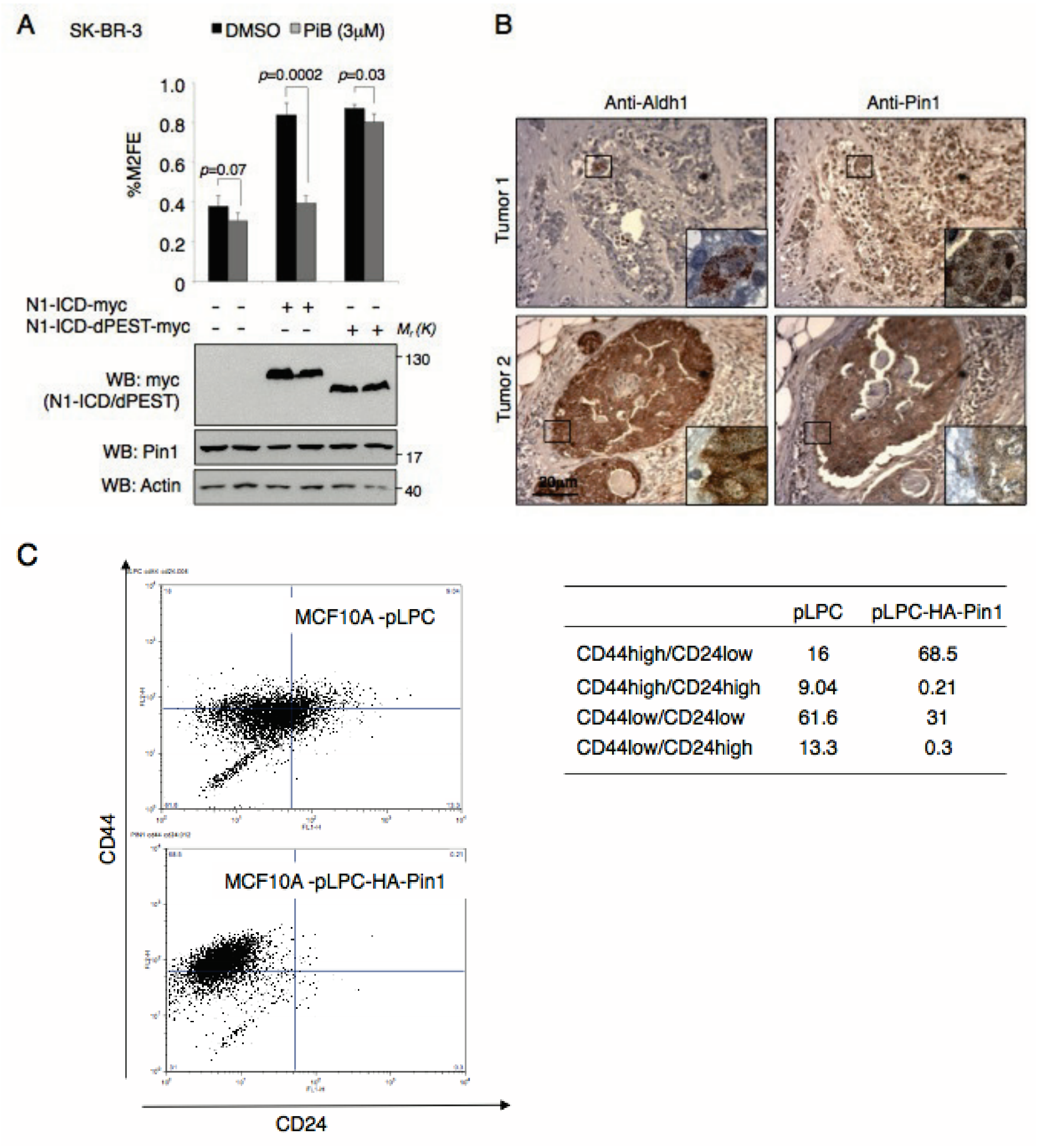


| | pLPC | pLPC-HA-Pin1 |
|---|---|---|
| CD44high/CD24low | 16 | 68.5 |
| CD44high/CD24high | 9.04 | 0.21 |
| CD44low/CD24low | 61.6 | 31 |
| CD44low/CD24high | 13.3 | 0.3 |

**Supporting Figure S3. (refers to Figure 3) Pin1 regulates breast CSCs self-renewal by keeping high levels of N1-ICD.**

**(A)** Upper: Percentage of secondary mammosphere formation efficiency (%M2FE) of SK-BR-3 cells transduced with empty (-), N1-ICD or N1-ICD-dPEST expressing vectors and treated with DMSO (black bars) or PiB (3 µM) (grey bars). Means, standard deviations and *p*-values (t-test, n=3) are indicated. Lower: Western blot analysis of the indicated proteins from cells grown as secondary mammospheres. **(B)** Representative bright field microphotographs at 100X magnification of immunohistochemical analyses of serial sections of two breast cancers with the indicated antibodies are shown. Part of the figure indicated with a square is shown by an inset at 400X magnification. Scale bar is indicated. **(C)** CD44/CD24 FACS analyses of MCF10A cells

transduced with pLPC or pLPC-HA-Pin1. Percentage of the different CD44/CD24 populations is indicated on the right.

**Supporting Information Figure S4.**

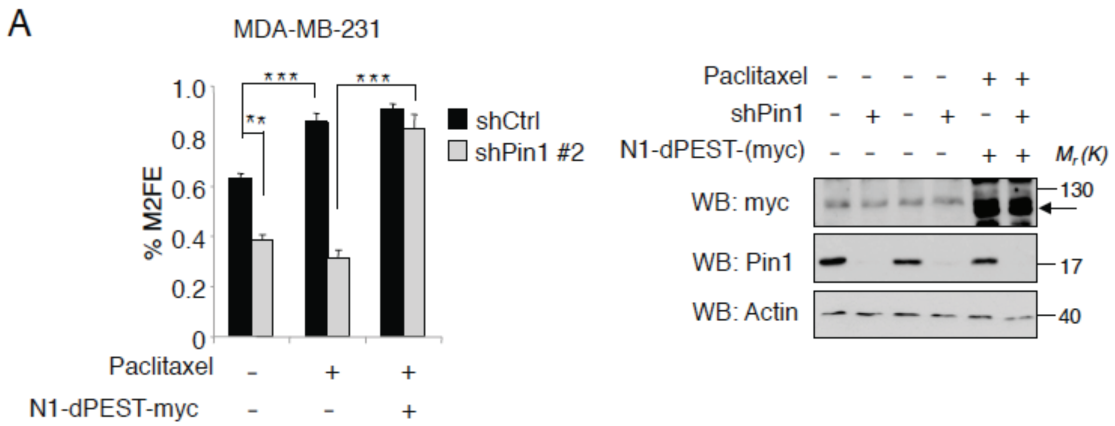


**Supporting Information Figure S4. (refers to Figure 4) Pin1 downmodulation sensitizes breast CSCs to chemotherapeutic treatment.**

**(A)** Left: Percentage of secondary MFE (%M2FE) of control (black bars) or Pin1 shRNA (sequence #2) (grey bars) expressing MDA-MB-231 cells transduced with empty (-) or N1-ICD-dPEST (+) expressing vectors, treated with Paclitaxel (+) or PBS (-). Means and standard deviations are indicated, p-values are ** = 0.0001, ***< 0.00003 (t-test, n=3). Right: the corresponding analysis of protein expression by Western blot is shown. Arrow indicates position of the specific band.

**Supporting Information Figure S5.**

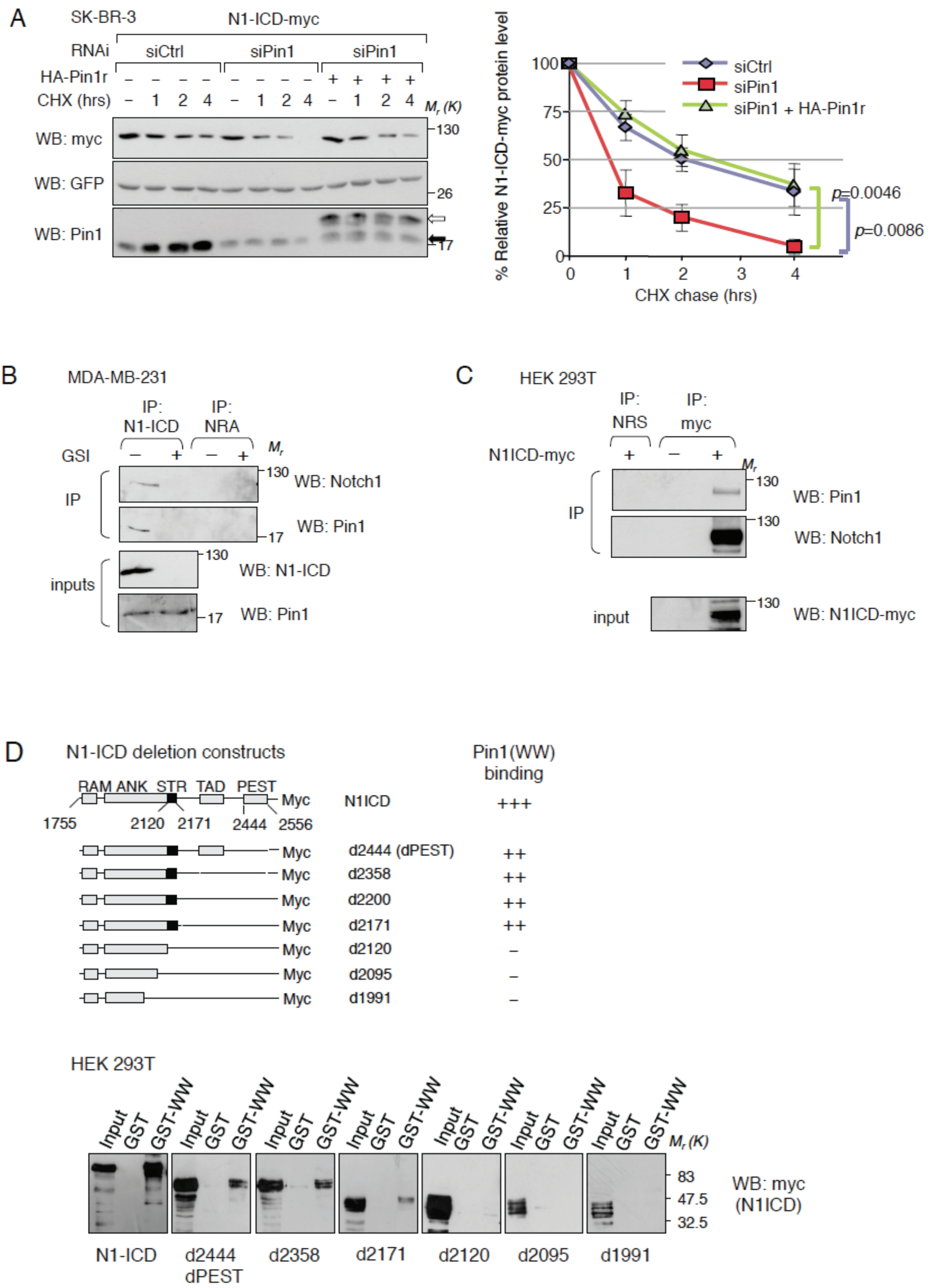

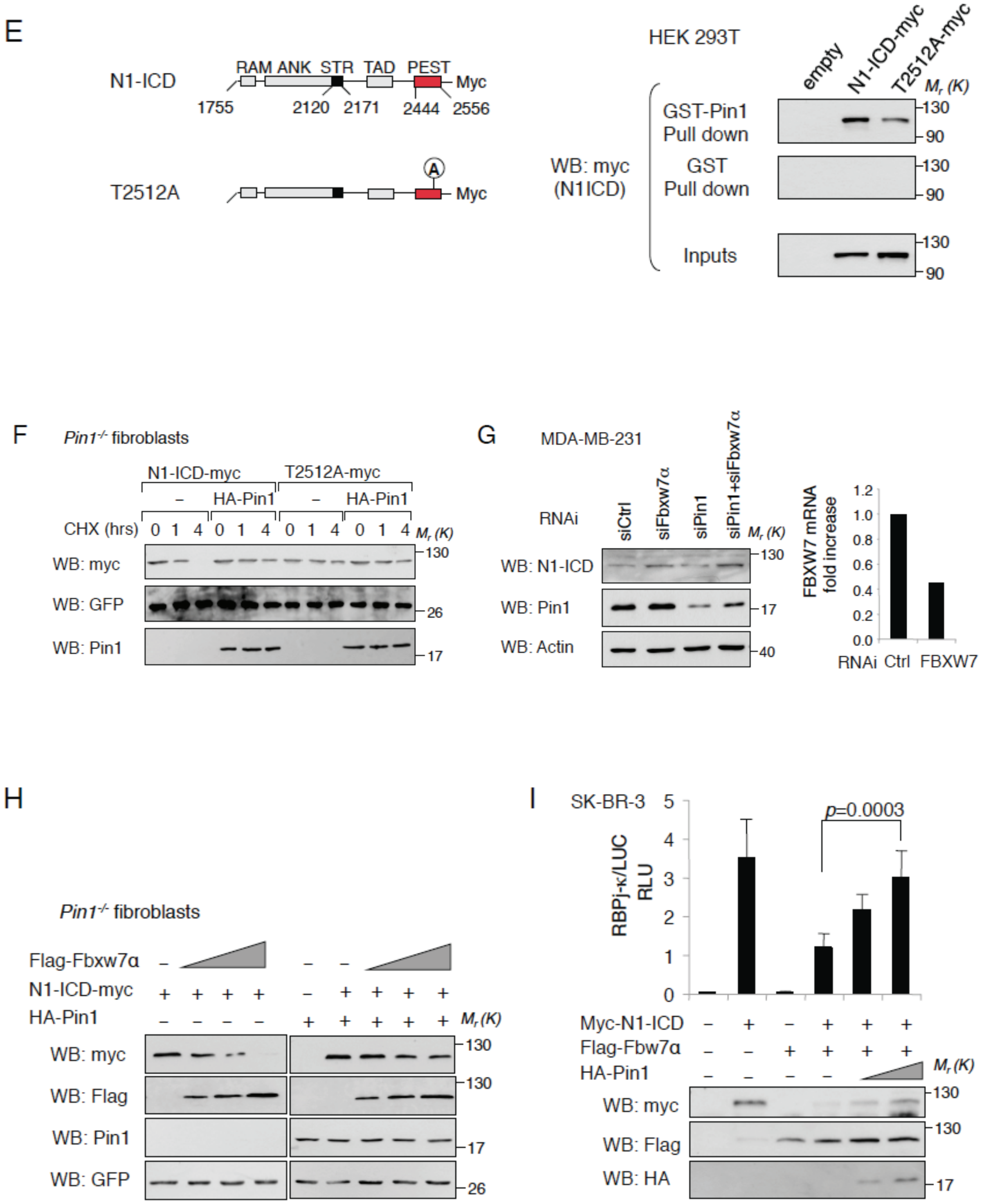
E
N1-ICD
RAM ANK STR TAD PEST
Myc
1755
2120
2171
2444
2556
T2512A
A
Myc
HEK 293T
empty
N1-ICD-myc
T2512A-myc
$M_r$ (K)
WB: myc
(N1ICD)
GST-Pin1
Pull down
GST
Pull down
Inputs
130
90
F
Pin1-/- fibroblasts
N1-ICD-myc
T2512A-myc
–
HA-Pin1
CHX (hrs)
0 1 4 0 1 4 0 1 4 0 1 4
WB: myc
WB: GFP
WB: Pin1
130
26
17
G
MDA-MB-231
RNAi
siCtrl
siFbxw7α
siPin1
siPin1+siFbxw7α
WB: N1-ICD
WB: Pin1
WB: Actin
40
FBXW7 mRNA
fold increase
1.2
1.0
0.8
0.6
0.4
0.2
0.0
RNAi Ctrl FBXW7
H
Pin1-/- fibroblasts
Flag-Fbxw7α
N1-ICD-myc
HA-Pin1
WB: myc
WB: Flag
WB: Pin1
WB: GFP
I
SK-BR-3
RBPj-κ/LUC
RLU
5
4
3
2
1
0
p=0.0003
Myc-N1-ICD
Flag-Fbw7α
HA-Pin1
WB: myc
WB: Flag
WB: HA

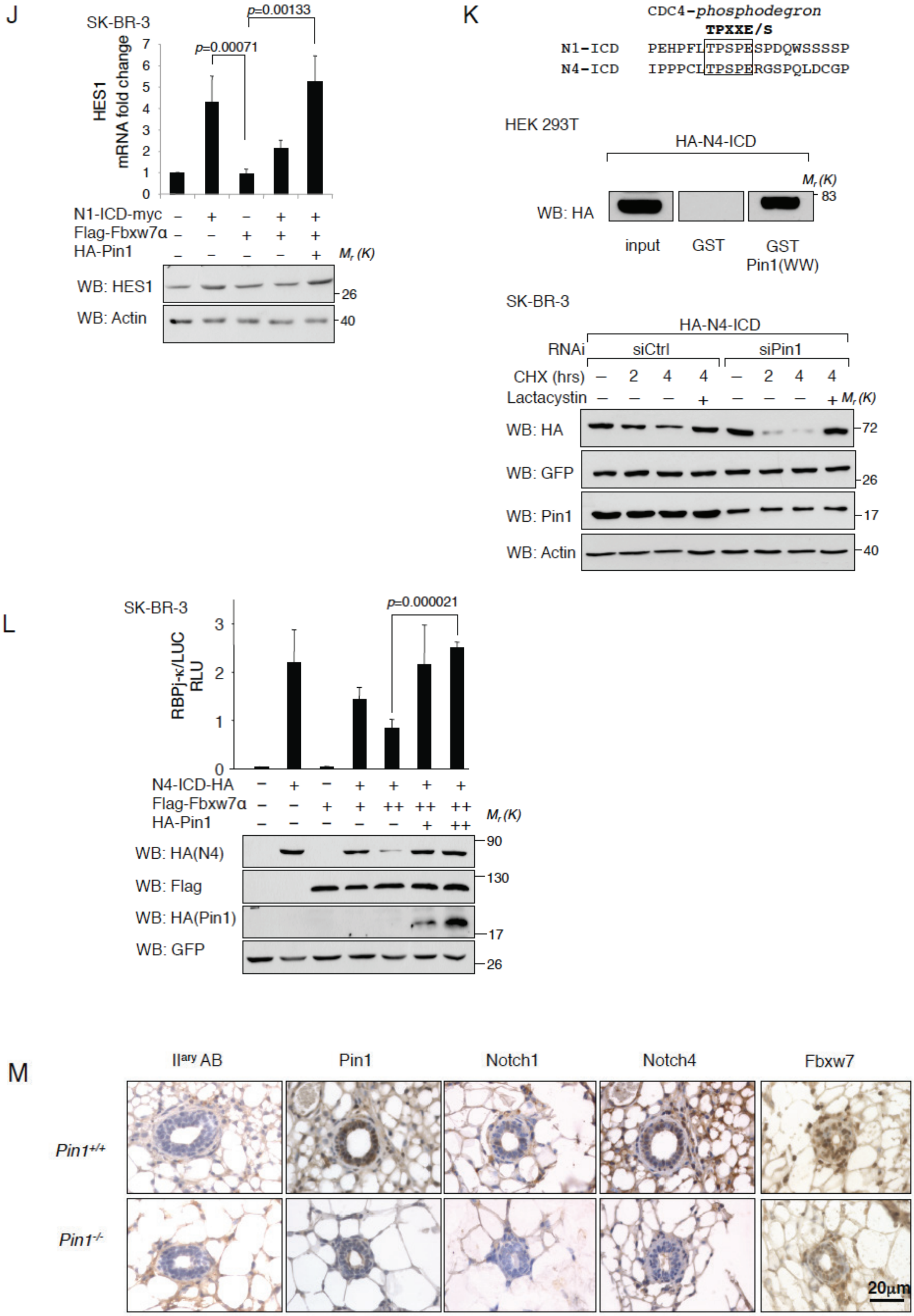

J
SK-BR-3
p=0.00133
p=0.00071
HES1 mRNA fold change
N1-ICD-myc
Flag-Fbxw7α
HA-Pin1
$M_r$ (K)
WB: HES1
WB: Actin
26
40
K
CDC4-phosphodegron
TPXXE/S
N1-ICD PEHPFLTPSPESPDQWSSSSP
N4-ICD IPPPCLTPSPERGSPQLDCGP
HEK 293T
HA-N4-ICD
WB: HA
83
input
GST
GST Pin1(WW)
SK-BR-3
HA-N4-ICD
RNAi
siCtrl
siPin1
CHX (hrs)
Lactacystin
WB: HA
WB: GFP
WB: Pin1
WB: Actin
72
26
17
40
L
SK-BR-3
p=0.000021
RBPj-κ/LUC RLU
N4-ICD-HA
Flag-Fbxw7α
HA-Pin1
WB: HA(N4)
WB: Flag
WB: HA(Pin1)
WB: GFP
90
130
17
26
M
II^ary AB
Pin1
Notch1
Notch4
Fbxw7
Pin1+/+
Pin1-/-
20µm

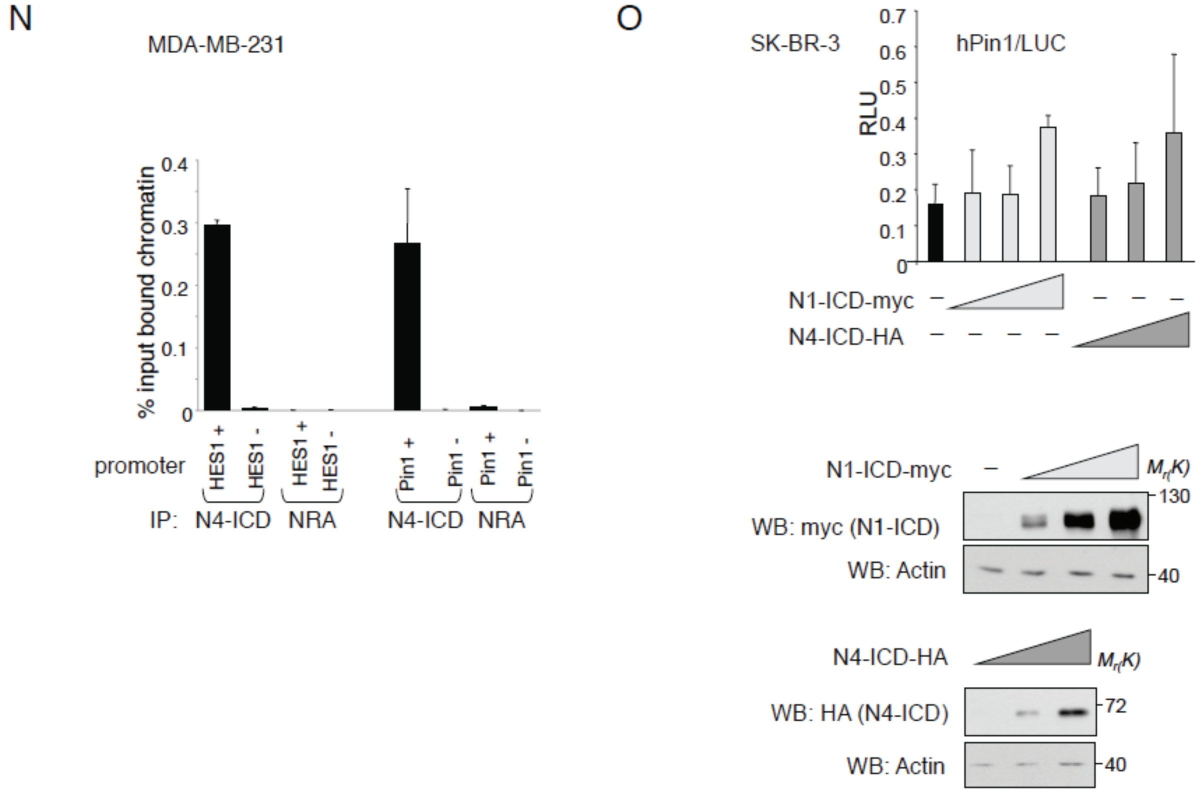


**Supporting Information Figure S5. (refers to Figure 5) Pin1 hampers Fbxw7α-mediated degradation of N1- and N4-ICDs.**

**(A)** Left panel: Western blot of over-expressed N1-ICD-myc along with indicated siRNAs and empty (-) or siPin1 resistant pCDNA3-HA-Pin1 (Pin1r) expression vectors, treated with DMSO (-) or CHX. Right panel: Graph indicating N1-ICD-myc protein levels *versus* time of CHX treatment in the three tested conditions (siCtrl, siPin1 and siPin1 + HA-Pin1r). Means, standard deviations and *p*-values (t-test, n=3) are indicated. **(B)** Non-related antibody (NRA) or anti-cleaved N1-ICD (Val1744) antibody immunoprecipitates from MDA-MB-231 cells, treated with GSI or vehicle (-), were subjected to either anti-Notch1 or anti-Pin1 Western blot analysis. Input lane shows 5% of total lysates. **(C)** N1-ICD binding to Pin1 is direct. Control non-related antibody (NRA) or anti-myc antibody immunoprecipitates from pcDNA3-N1ICD-myc transfected HEK 293T cells, were subjected to Far Western using purified GST-Pin1 as a probe, followed by anti-Pin1 Western blot. Anti-myc Tag Western analysis of the upper panel after stripping is shown. **(D)** Mapping of the Pin1-N1-ICD interaction. Upper: Schematic of pCDNA3-N1-ICD-myc C-terminal deletion constructs (from d2444 to d1991) used for mapping Pin1 binding domains in N1-ICD. TM: transmembrane, RAM: CSL interacting, ANK: ankyrin, STR: Serine-Threonine rich, TAD: transactivation, PEST: Pest domain. Numbering refers to Swissprot entry P46531. Interaction with Pin1 is indicated next to the constructs: +++ very strong, ++ strong, – no binding. A relevant drop in binding strength is observed by deletion of the PEST domain and the STR. Lower: Representative anti-myc tag Western blot analyses of GST-Pin1 pull down assays of indicated proteins overexpressed in HEK 293T cells is shown. The borders around the panels demarcate

juxtaposed parts of same or different gels aligned in function of the molecular weight. **(E)** N1-ICD-T2512A mutation hampers binding to Pin1. Left: scheme representing wildtype N1-ICD and T2512A mutation. Right: Anti-myc tag Western blot analysis of GST-Pin1 or GST pull down assay and input levels of indicated proteins overexpressed in HEK 293T cells is shown. **(F)** N1-ICD-T2512A mutant half-life is unaffected by Pin1 levels. Western blot of CHX chase experiment with N1-ICD-myc and N1-ICD-T2512A-myc mutant with empty (-) or HA-Pin1 expressing vectors. **(G)** Western blot analysis showing the levels of N1-ICD following RNA interference (RNAi) of the indicated factors. Modulation of Pin1 levels are shown in the same Western blot, whereas the relative decrease of *FBXW7* mRNA was detected by qRT-PCR, as shown in the histogram on the right. **(H)** $Pin1^{-/-}$ MEFs transfected with pCDNA3-N1-ICD-myc and increasing amounts of p3xFLAG-Fbxw7α along with either empty vector (-) or pCDNA3-HA-Pin1. The borders around the panels demarcate juxtaposed parts of the same gel. **(I)** Histogram of Luciferase reporter assays in SK-BR-3 cells co-transfected with pGL2-RBPjκ/LUC and empty vector (-) or pCDNA3-N1-ICD-myc, along with p3xFLAG-Fbxw7 and increasing amounts of pCDNA3-HA-Pin1 as indicated by a wedge. Means, standard deviations and *p*-value (t-test, n=3) are indicated. Cell lysates were analyzed by Western Blot and are shown below. **(J)** Histogram showing the fold change of mRNA expression of endogenous N1-ICD target (HES-1) following over-expression of N1-ICD-myc, Flag-Fbxw7α and HA-Pin1 in SK-BR-3 cells analyzed by qRT-PCR. Means and standard deviations are indicated for three independent experiments. The corresponding Western blot is shown below. **(K)** Upper: Alignment of cdc4-*phophodegron* sequence within the PEST domains of Notch1 and Notch4 (Swissprot P46531 and Q99466, respectively). The consensus is shown in bold, the aminoacids of N1- and N4-ICD corresponding to the *phosphodegron* are boxed. Middle: Western blot of a GST pull down analysis performed with GST-Pin1 binding domain WW and GST alone with overexpressed HA-N4-ICD. Input levels are indicated. The borders around the panels demarcate juxtaposed parts of the same gels. Lower: Western blot of a CHX chase in SK-BR-3 cells transfected with pCDNA4-N4-ICD-HA along with the indicated RNAi, in presence (+) or absence of proteasome inhibitor Lactacystin. **(L)** Histogram of Luciferase reporter assays in SK-BR-3 cells co-transfected with pGL2-RBPjκ/LUC and pCDNA4-N4-ICD-HA alone or with p3xFlag-Fbxw7α and increasing amounts of pCDNA3-HA-Pin1. Means, standard deviations and *p*-value (t-test) are indicated for three independent experiments. Cell lysates were analyzed by Western blot and are shown below the histograms. **(M)** Microscope images at 400X magnification of sectioned mammary glands from 8 weeks old female mice stained with the indicated antibodies by immunohistochemistry and counterstained with haematoxylin. Stainings were performed without primary antibody (only secondary, $II^{ary}$ AB) for specificity and with anti-Pin1 antibody as genotype control, respectively. Scale bar is indicated. **(N)** Histogram representing the means and standard deviations of ChIP analyses (n=3) with anti-N4-ICD (Val 1432) or a non related antibody

(NRA) in MDA-MB-231 cells on endogenous HES1 or Pin1 promoters, using specific RBP-jκ binding sites (HES1+ and Pin1+) or unrelated regions as negative controls (HES1- and Pin1-) on both promoters (Rustighi et al., 2009). **(O)** Histogram of Luciferase reporter assays in SK-BR-3 cells co-transfected with the luciferase under control of the human Pin1 promoter (pGL2-hPin1/LUC) along with increasing amounts of pCDNA4-N4-ICD-HA or pCDNA3-N1-ICD as a positive control (Rustighi et al., 2009). Means, and standard deviations are indicated for three independent experiments. Cell lysates were analyzed by Western blot and are shown below the histograms. **(A)-(L), (O)** Molecular weights $M_r$(K) are indicated in KDa.

**Supporting Information Figure S6.**

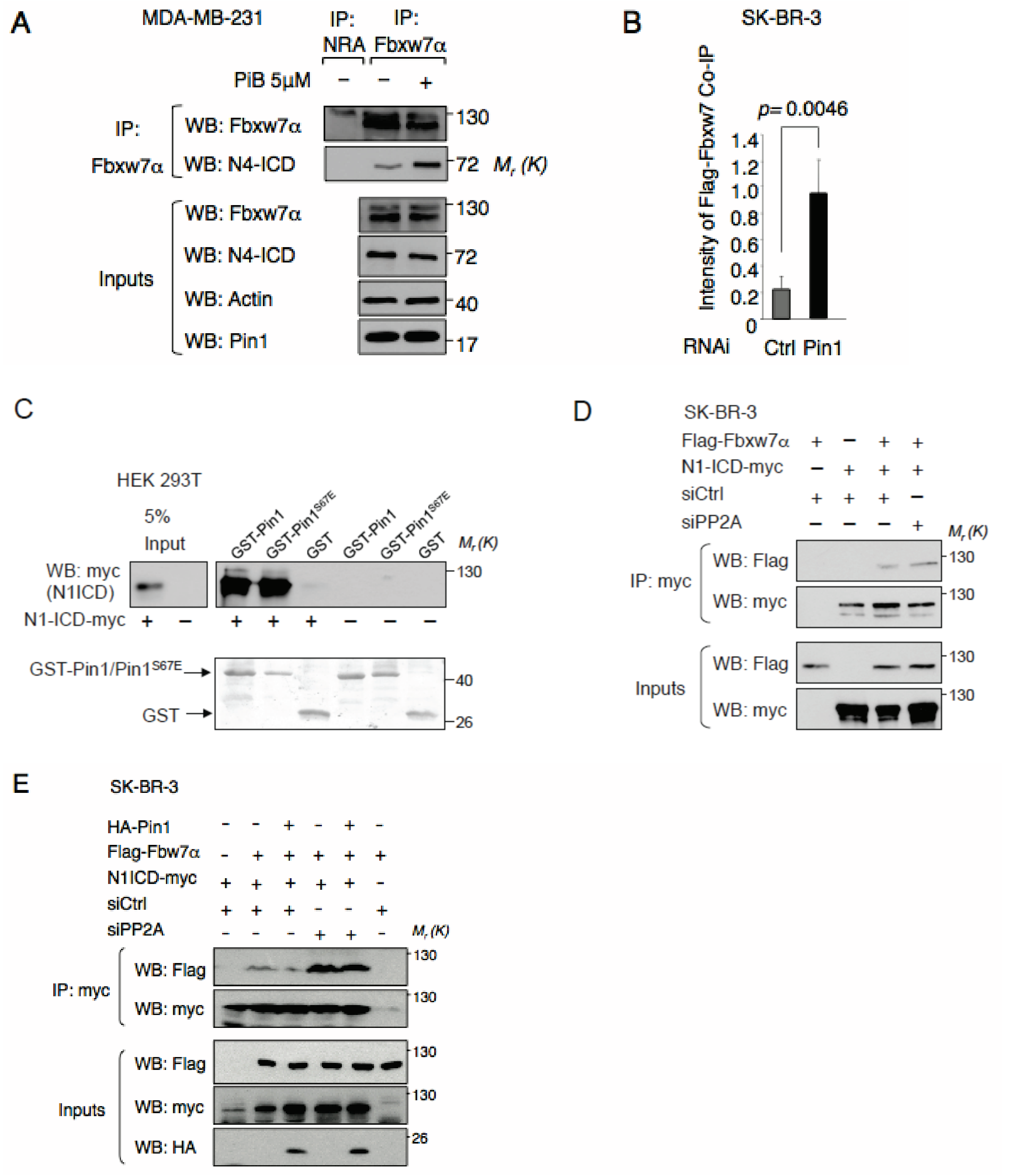


**Supporting Figure S6. (refers to Figure 6) Pin1 uncouples N1-ICD and N4-ICD from Fbxw7α in a PP2A dependent manner.**

**(A)** Western blot analysis of Co-IP experiments between endogenous Fbxw7α and N4-ICD from MDA-MB-231 cells treated with DMSO or PiB. Anti-Fbxw7α immunoprecipitates (IP) were recognized with anti-Notch4 antibodies and after stripping with an anti-Fbxw7α antibody. Input levels are shown below. **(B)** Graph depicting the mean of Flag-Fbxw7α Co-IP levels, normalized to immunoprecipitated N1-ICD. Standard deviations and *p*-value (t-test, n=3) are shown. **(C)** Upper panel: Western blot analysis of GST pull-down experiments using GST-Pin1 or GST-Pin1$^{S67E}$

with lysates from HEK 293T cells transfected with empty (-) or N1-ICD-myc expressing vector. Input levels of N1-ICD are shown. The borders around the panels demarcate juxtaposed parts of the same gels cropped from different positions. Lower panel: Ponceau staining of the indicated GST proteins transferred on the membrane used in the upper panel. **(D)** Western blot analysis of Co-IP between N1-ICD-myc and Flag-Fbxw7α from SK-BR-3 cells transfected with indicated vectors along with control (siCtrl) or PP2A (siPP2A) specific siRNA. Anti-Flag Western blot to reveal Flag-Fbxw7α Co-IP and, after stripping, anti-myc Western blot of immunoprecipitates (IP) are shown. Inputs are shown below. **(E)** Western blot analysis as in (C) with or without overexpression of HA-Pin1. **(A), (C)-(E)** Molecular weights ($M_r$ (K) are indicated in KDa.

**Supporting Information Figure S7.**

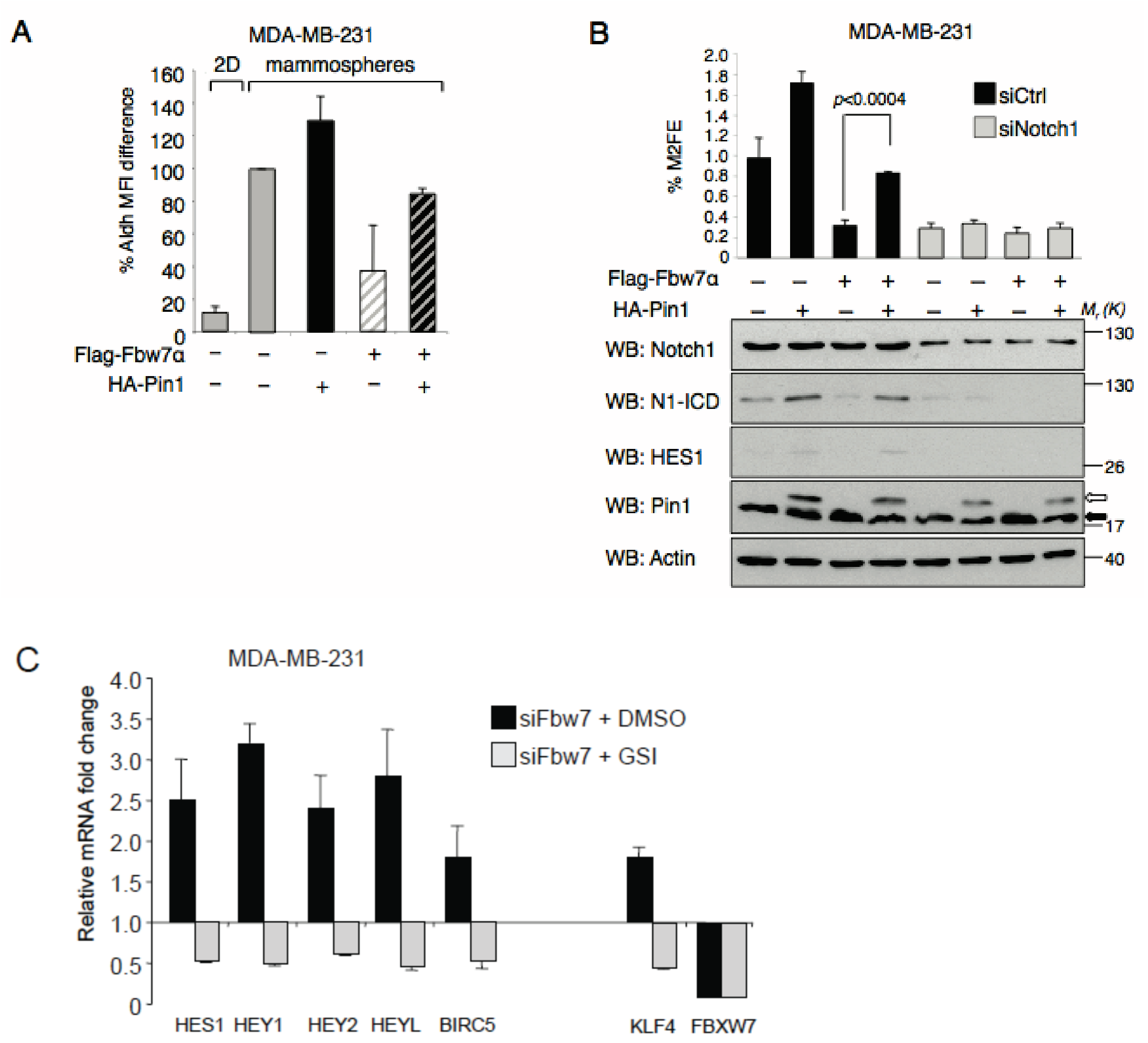


**Supporting Figure S7. (related to Figure 7) Pin1 sustains Notch-dependent stem cell features despite Fbxw7α.**

**(A)** Percentage of Aldh positive cells of clones from Figure 7A grown as mammospheres, calculated as Mean fluorescence intensity (MFI) difference between samples containing BAAA and that of the same samples containing also DEAB. **(B)** Histogram of the percentage of secondary mammosphere forming efficiency (%M2FE) of the same clones as in Figure 7A in presence of control- (siCtrl) or Notch1 RNA interference (siNotch1). Means, standard deviations and *p*-value (t-test) of three independent experiments, are indicated. Protein levels are shown in the Western blot below. White and black arrows indicate over-expressed and endogenous Pin1, respectively. Molecular weights ($M_r$) are indicated in KDa. **(C)** qRT-PCR of Notch target genes of MDA-MB-231 transfected with Fbxw7-specific siRNA and treated with vehicle (black bars) or GSI (grey bars). Means and standard deviations are indicated (n=3).

**Supporting Information Figure S8.**

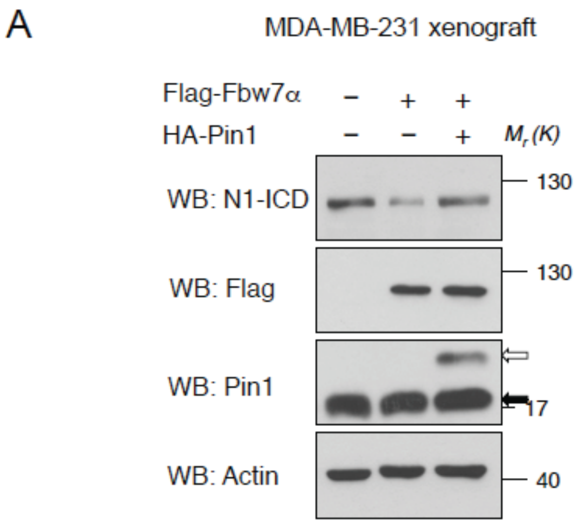


**Supporting Figure S8. (related to Figure 8A) Pin1 sustains Notch-dependent stem cell and tumorigenic features despite Fbxw7α *in vivo*. (A)** Western blot of Fbxw7α and Fbxw7α + Pin1 tumor xenografts relative to control tumors (empty) explanted at the end of the experiment.

**Supporting Information Figure S9**

A

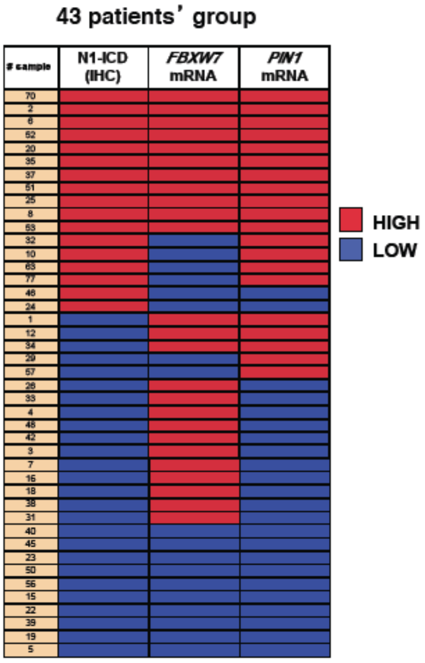


| | | FBXW7 | | PIN1 | |
|---|---|---|---|---|---|
| N1-ICD high | 17 (39.5%) | high | 11 (64.7%) | high | 11 (100%) |
| | | | | low | 0 (0.0%) |
| | | low | 6 (35.3%) | high | 4 (66.7%) |
| | | | | low | 2 (33.3%) |
| N1-ICD low | 26 (60.5%) | high | 14 (53.8%) | high | 3 (21.4%) |
| | | | | low | 11 (78.6%) |
| | | low | 12 (46.2%) | high | 2 (16.7%) |
| | | | | low | 10 (83.3%) |

B

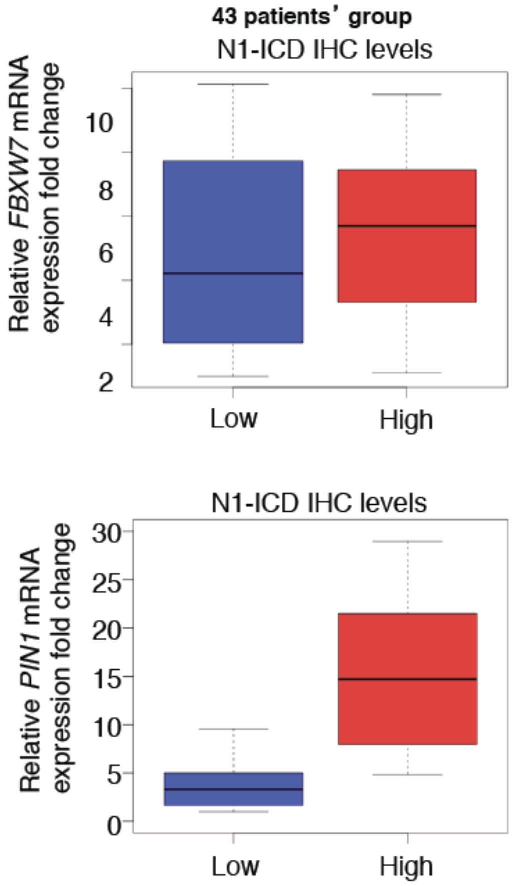


C

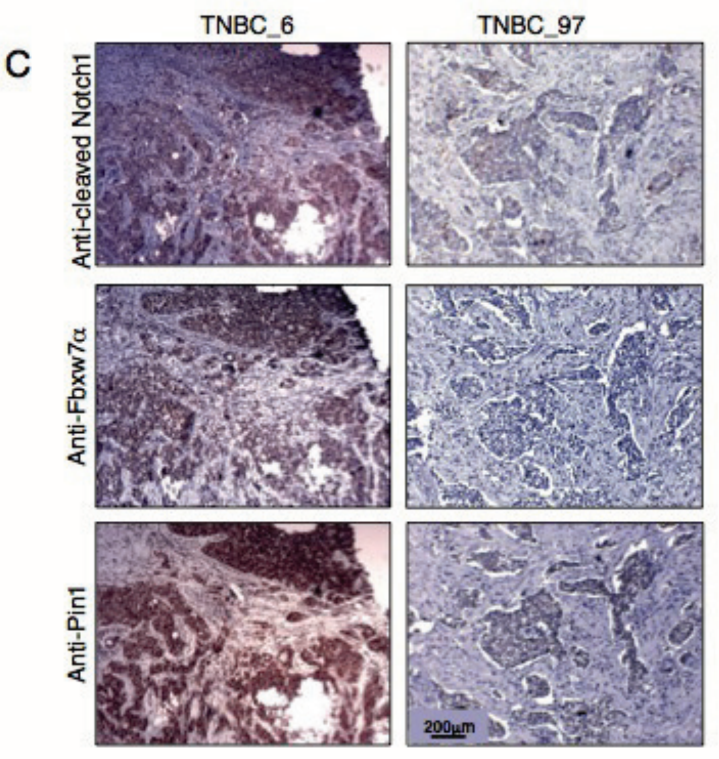

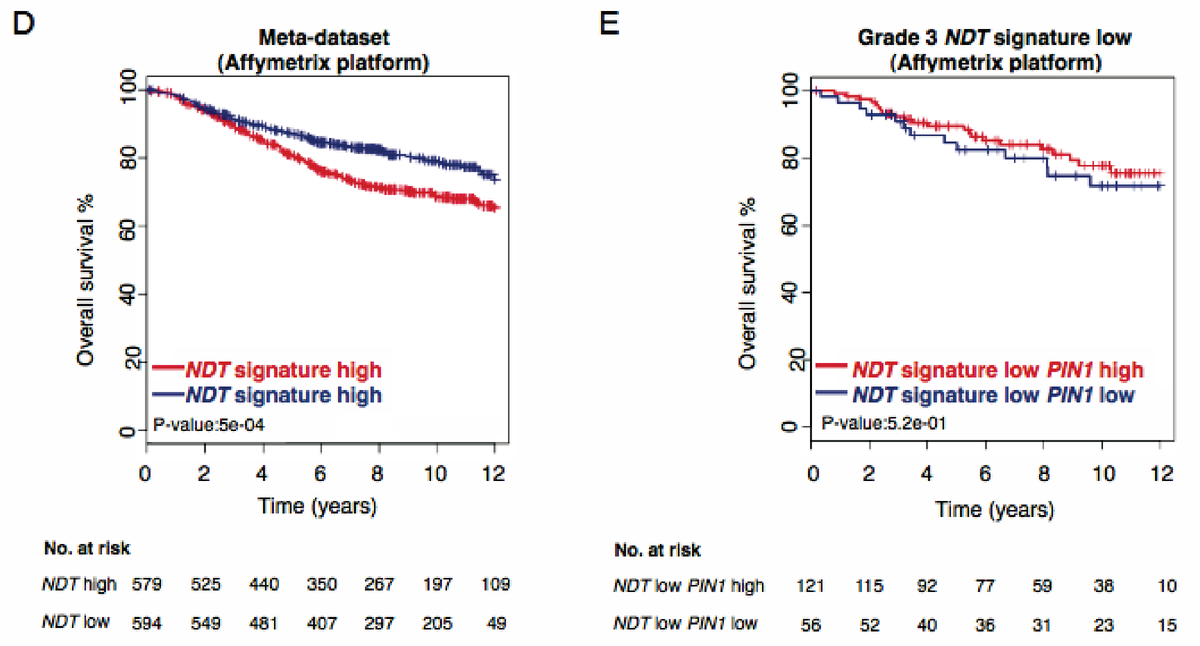


**Supporting Figure S9. (related to Figure 9) Association of *FBXW7* or *PIN1* expression in N1-ICD high and low breast cancers.**

**(A)** Upper panel: heatmap representing the expression of *PIN1* and *FBXW7* (detected at mRNA expression level) and N1-ICD (detected at protein level) in a cohort of 43 breast cancer patients. The colour in each block represents if the mRNA or protein is above (red) or below (blue) the average value of the samples for each gene or was scored high or low by immunohistochemistry, respectively. Lower panel: Contingency table showing percentage of each category calculated on the precedent category of patients. **(B)** Box-plot representations of gene expression values (of *FBWX7* and *PIN1*, upper and lower panels, respectively) in the breast cancer cohort of Figure S9A. Patients were binned in low or high categories for weak or strong N1-ICD staining (detected by IHC, see Methods for detail). Finally, to test the associations between N1-ICD staining and expression of *FBWX7* and *PIN1*, Wilcoxon rank sum tests were performed. The results showed no association for *FBWX7* (W = 192, p-value = 0.8998) and very strong association for *PIN1* (W = 35, p-value = $2.967 \times 10^{-6}$). **(C)** Representative images at 40X magnification of indicated protein expression analyses by immunohistochemistry of serial sections of tissues from the patients' cohort in Fig. 9A. Scale bar is indicated. **(D)** Kaplan-Meier graphs representing the probability of survival in 1173 breast cancer patients from the meta-dataset of Figure 9C with outcome information on survival. Tumor samples were classified as high or low Notch-dependent Direct Target (*NDT*) gene signature using the classifier described (Adorno et al., 2009). The log-rank test p value reflects the significance of the association between *NDT* gene signature low and longer survival. **(E)** Kaplan-Meier graphs representing the probability of survival in grade 3 patients from the meta-dataset with low *NDT* signature classified for high or low *PIN1* levels.

**Supporting Information Table S1**. Breast cancer re-organized cohorts comprised in the meta-dataset analyzed in this study.

| Cohort | Affymetrix platform | Samples | Data source | References |
|---|---|---|---|---|
| KI_Stockholm | HG-U133 A | 159 | GSE1456 | Pawitan et al., 2005 |
| EMC-344 | HG-U133A | 344 | GSE2034; GSE5327 | Wang et al., 2005; Minn et al., 2007 |
| MSKCC | HG-U133A | 82 | GSE2603 | Minn et al., 2005 |
| KI_Uppsala | HG-U133A | 253 | GSE3494; GSE4922; GSE6532 | Loi et al, 2008; Ivshina et al, 2006; Miller et al, 2005 |
| OXF | HG-U133A | 178 | GSE6532 | Ivshina et al., 2006 |
| TransBIG | HG-U133A | 198 | GSE7390 | Desmedt et al., 2007 |
| Mainz | HG-U133A | 200 | GSE11121 | Schmidt et al., 2008 |
| Veridex_MultiCenter | HG-U133A | 136 | GSE12093 | Zhang et al., 2009 |
| GUY | HG-U133 Plus 2.0 | 164 | GSE6532; GSE9195 | Loi et al., 2007; Loi et al., 2008; Loi et al., 2010 |
| UCSF | HG-U133AAofAV2 | 166 | E-TABM-158; GSE7378 | Merritt et al., 2008; Zhou T et al., 2007; Yau C et al., 2008 |
| IJB_TOP | HG-U133 Plus 2.0 | 107 | GSE16446 | Desmedt Cet al., 2011; Li Y et al., 2010; Juul N et al., 2010 |
| US_NCI | HG-U133 Plus 2.0 | 115 | GSE19615 | Li Y t al., 2010 |
| CRCM | HG-U133 Plus 2.0 | 252 | GSE21653 | Sabatier R et al., 2011 |
| KOOF | HG-U133 Plus 2.0 | 327 | GSE20685 | Kao KJ et al., 2011 |
| Goethe | HG-U133A | 65 | GSE31519 | Rody A. et al., 2011; Karn T. et al., 2011; Karn T. et al., 2012 |
| MDACC | HG-U133A | 313 | GSE25066 | Hatzis C. et al., 2012 |
| I-SPY-1 | HG-U133A | 83 | GSE25066 | Hatzis C. et al., 2012 |
| LBJ_INEN_GEICAM | HG-U133A | 58 | GSE25066 | Hatzis C. et al., 2012 |
| USO-02103 | HG-U133A | 54 | GSE25066 | Hatzis C. et al., 2012 |

**Supporting Information Table S2.** Notch-dependent Direct Target (NDT) gene signature.

| Gene name | ENTREZ ID | Reference |
|---|---|---|
| HES1 | 3280 | Grabher et al., 2006 |
| HEY1 | 23462 | Grabher et al., 2006 |
| HEY2 | 23493 | Grabher et al., 2006 |
| HEYL | 26508 | Grabher et al., 2006 |
| MYC | 4609 | Palomero et al., 2006; Weng et al., 2006 |
| BUB1B | 701 | Palomero et al., 2006 |
| BUB3 | 9184 | Palomero et al., 2006 |
| CDC25A | 993 | Palomero et al., 2006 |
| PHB | 5245 | Palomero et al., 2006 |
| RBL1 | 5933 | Palomero et al., 2006 |
| RPL3 | 6122 | Palomero et al., 2006 |
| USP5 | 8078 | Palomero et al., 2006 |
| PLAU | 5328 | Shimizu et al., 2011 |
| SHQ | 55164 | Chadwick et al., 2009 |
| CCND1 | 595 | Ronchini and Capobianco, 2001 |
| GATA3 | 2625 | Amsen et al., 2007 |
| SKP2 | 6502 | Sarmento et al., 2005 |
| ERBB2 | 2064 | Chen et al., 1997 |
| CDKN1A | 1026 | Rangarajan et al., 2001 |
| SNAI1 | 6615 | Sahlgren et al., 2008 |
| SNAI2 | 6591 | Leong et al., 2007 |
| NFKB2 | 4791 | Oswald et al., 1998 |
| BIRC5 | 332 | Lee et al., 2008 |
| NOTCH1 | 4851 | Weng et al., 2006; Hamidi et al., 2011 |
| NOTCH3 | 4854 | Weng et al., 2006; Hamidi et al., 2011 |
| NOTCH4 | 4855 | Hamidi et al., 2011 |
| IFRD2 | 7866 | Palomero et al., 2006 |
| ING3 | 54556 | Palomero et al., 2006 |
| PTCRA | 171558 | Grabher et al., 2006 |
| CD3D | 915 | Palomero et al., 2006 |

**Supporting Information Table S3.** Enrichment of gene signatures in the list of genes preferentially expressed in *NDT* signature HIGH/*PIN1* HIGH versus *NDT* signature HIGH/*PIN1* LOW tumors in the metadataset (GRADE 3).

| **SIGNATURES (BIOCARTA PATHWAY + OTHER SIGNATURES)** | **ENRICHMENT in *NDT* HIGH/*PIN1* HIGH (q-value)** |
|---|---|
| ES1 | 0.008710 |
| ES.like | 0.021817 |
| stem_tumorig | 0.029714 |
| Mut.p53_up | 0.034324 |

Differentially expressed genes (n=1460, q-value <0.01 and fold change >1.5) were subjected to Gene set enrichment analysis (GSEA) as already described (Montagner et al., 2012). Enrichment has been determined using a Fischer's exact test on all 254 signaling pathways in the database. P-values have been corrected using BH procedure.

## Supporting Material and Methods

**Oligonucleotides.** All oligonucleotides were supplied by MWG.

### Oligonucleotides for quantitative real-time PCR:

| Gene symbol | Forward 5'-3' | Reverse 5'-3' | Ref |
|---|---|---|---|
| Mouse Pin1 | GTCTCAGGGATGGGGCTTTT | TGGTGGGGCTCAGAGGTATT | |
| Mouse SMA | TGATCACCATTGGAAACGAACG | TGGTTTCGTGGATGCCCGCT | Stingl et al., 2006 |
| Mouse CK14 | TGAGAGCCTCAAGGAGGAGC | TCTCCACATTGACGTCTCCAC | Stingl et al., 2006 |
| Mouse CK18 | CTTGCTGGAGGATGGAGAAG | CTGCCATCCACGATCTTACGG | Stingl et al., 2006 |
| Mouse CK19 | CAGGCTGGAGCAGGAGATCG | TGGTAGCTCAGATGGCCTTGG | Stingl et al., 2006 |
| Mouse GAPDH | TTCACCACCATGGAGAAGGC | CCCTTTTGGCTCCACCCT | Li et al., 2009 |
| Human HES1 | GAGAAAAGACGAAGAGCA | TGTGCTCAGCGCAGCCGT | |
| Human HEYL | TCCCCACTGCCTTTGAG | GGCACTCTTCCCAGGAT | Leong et al., 2007 |
| Human BIRC5 | GCCCAGTGTTTCTTCTGCTT | CCGGACGAATGCTTTTTATG | Cheung et al., 2009 |
| Human CTGF | GTCCGCGTCGCCTTCGTGGTC | GAGCACCATCTTTGGCGGTGCAC | |
| Human SLUG | AGATGCATATTCGGACCCAC | CCTCATGTTTGTGCAGGAGA | Leong et al., 2007 |
| Human ABCG2 | TTTCCAAGCGTTCATTCAAAAA | TACGACTGTGACAATGATCTGAGC | |
| Human GLI1 | CCCCAGGGGCTGAGTCCTCC | TCCAGAGCTGCCCCGCTGAT | |
| Human PTCH | CTGAGCAACACTCTGATGAA | CAGTTAATGACTCCCAAGCA | |
| Human BMI-1 | GTCCAAGTTCACAAGACCAGACC | ACAGTCATTGCTGCTGGGCATCG | Liu et al., 2006 |
| Human VIM-1 | GAGAACTTTGCCGTTGAAGC | GCTTCCTGTAGGTGGCAATC | Casas et al., 2011 |
| Human HMGA2 | AGCAGAAGCCACTGGAGAAA | TCTTCGGCAGACTCTTGTGA | |
| Human KLF4 | ATTACGCGGGCTGCGGCAAAA | TTTTGGCACTGGAACGGGCGG | |
| Human DKK | GGGAATTACTGCAAAAATGGAATA | ATGACCGGAGACAAACAGAAC | Butler., 2010 |
| Human CDH1 | TGCCCAGAAAATGAAAAAGG | GTGTATGTGGCAATGCGTTC | Casas et al., 2011 |
| Human Pin1 | CTGGAGCTGATCAACGGCTACATCC | GCAGCGCAAACGAGGCGTCT | |
| Human Fbxw7 | GTGGACCTGCCCGTTCACCAACTCT | CGGACCTCAGAACCATGGTCCAACT | |
| Human GAPDH | GAGTCAACGGATTTGGTCGT | GACAAGCTTCCCGTTCTCAG | |
| Human H3 | GTGAAGAAACCTCATCGTTACAGGC CTGGT | CTGCAAAGCACCAATAGCTGCACTC TGGAA | |

### Oligonucleotides for cloning and mutagenesis:

| Plasmids | Forward 5'-3' | Reverse 5'-3' |
|---|---|---|
| pLKO-TetO-shPin1 | CCGGCGGGAGAGGAGGACTTTGACTCGAGTC AAAGTCCTCCTCTCCCGTTTTT | AATTAAAAACGGGAGAGGAGGACTTTGACTCG AGTCAAAGTCCTCCTCTCCCG |
| pCDNA3-N1-ICD Threonine 2512 to Alanine | CACCCCTTCCTCGCGCCGTCCCCTGAGTCCCC TGAC | GAGGAAGGGGTGCTCAGGCACCTGTAG |
| pMSCV-3XFlag-Fbxw7α | TATAAAGATCTTATGATCGACTACAAAGACG AT | GAGCGGATCCATGGCACGCAAGCGCCGG |

### siRNA sequences:

| siRNA | Sequence 5'-3' | Ref |
|---|---|---|
| siFbxw7α | ACCTTCTCTGGAGAGAGAAATGC | Welcker et al., 2004 |

| | | |
|---|---|---|
| siPP2A | CACCUUUGGGCAAGAUAUU | Guichard et al., 2006 |
| siPin1 #1 | CGGGAGAGGAGGACUUUGA | Girardini et al., 2011 |
| siPin1 #2 | GCCAUUUGAAGACGCCUCG | Girardini et al., 2011 |
| siNotch1 | GUGUCUGAGGCCAGCAAGA | |
| siCtrl | Allstar NEGATIVE control, Qiagen | |

**Plasmid, retroviral and lentiviral vectors.**

pCDNA3-N1-ICD-myc, pCDNA3-N1-ICD-deltaPEST(d2444)-myc, pCDNA3-N1-ICD-deltaTAD(2171)-myc, pCDNA3-N1-ICD-deltaSTR(d2120)-myc, pCDNA3-N1-ICD-T2512A-myc, were generated by PCR and standard cloning procedures starting from pCDNA3-NdeltaE-myc (Rustighi et al., 2009) constructs as templates. pCDNA3-N1-ICD-(d2095)-myc and pCDNA3-N1-ICD-(d1991)-myc were generated as above from corresponding pBabe constructs described elsewhere (Jeffries and Capobianco, 2000). pCDNA3-HA-Pin1 and pCDNA3-HA-S67E, retroviral pLPC-HA-Pin1, pCDNA3-HA-Pin1r, were already described (Rustighi et al., 2009; Girardini et al., 2011). Retroviral pMSCV-3X-Flag-Fbxw7α was obtained by PCR and subcloning from p3X-Flag-Fbxw7α coding for the human alpha isoform. pCDNA4-N4-ICD-HA was a kind gift of I. Prudovsky and was already described (MacKenzie et al., 2004). For lentiviral infection an shPin1 corrsponding to the siRNA sequence Pin1#1 was cloned into the Doxicyclin-inducible pLKO-Tet-ON (Addgene) following the "All-in-one" system described by D.Wiederschain (dmitri.wiederschain@novartis.com). For preparation of viral particles psPAX2 and pMD2.G were used in HEK 293T cells.

**Cell lines and treatments**

MDA-MB-231, SK-BR-3, BT-549, and SUM-159 are human breast carcinoma cells, HEK 293T is a human embryonic kidney cell line with SV40 large T, immortalized Pin1-/- fibroblasts have been obtained by spontaneous immortalization from Mouse Embryo Fibroblasts of C57BL6/129Sv mixed background (Rustighi et al., 2009). COS-7 are monkey kidney cells immortalized with SV40 large T antigen. NOP6 is a mouse mammary tumor cell line (Yang et al., 2009). All cells were cultured in DMEM, supplemented with 10% Fetal Bovine Serum (Gibco) and Penicillin/Streptomycin, as described (Rustighi et al., 2009). For NOP6, in addition Insulin supplements were added (Yang et al., 2009). MCF10A cells were maintained in DMEM:F12 Ham's (1:2; Sigma), supplemented with 5% horse serum (Gibco), insulin (10 μg $ml^{-1}$; Sigma), hydrocortisone (0.5 μg $ml^{-1}$) and EGF (20 ng $ml^{-1}$; Peprotech). Primary breast cancer cell lines were maintained in F12 Ham's supplemented with 10% FCS and insulin, hydrocortisone and EGF as above. Transient transfections, retroviral or lentiviral infections were performed by standard procedures, as described (Rustighi et al., 2009). pEGFP was included as control for transfection efficiency. For creation of stable clones, a selection corresponding to the expressed vectors was applied for 2 weeks to infected cells at the concentrations of 2 μgr/ml for Puromycin and 0,5mg/ml for Blasticidin. Cycloheximide (Sigma) for chase experiments was used at 50 nM concentration. Treatment with gamma-secretase inhibitor DAPT (Sigma), Pin1 inhibitor PiB (Calbiochem) or proteasome inhibitor lactacystin (Sigma) have been described previously (Rustighi et al., 2009).

Protein phosphatase inhibitor Okadaic Acid (Sigma) was dissolved in DMSO and used at a final concentration of 200 nM for 6h.

**Flow cytometric analyses and sorting (FACS)**

Mouse mammary epithelial lineage-depleted cells, pre-enriched using the EasySep Mouse Mammary Stem Cell Enrichment Kit (see above), were analysed by FACS and sorted to near purity (85%) with antibodies against CD49f and CD24. FACS analysis and sorting from mammospheres based on aldehyde dehydrogenase (Aldh) activity were performed using the Aldefluor kit (StemCell Technologies Inc) following the manufacturer's instructions and are detailed in Supporting Information. Briefly, cells were incubated with active substrate (BAAA) in presence or absence of a specific adehyde dehydrogenase inhibitor (DEAB) for 30-60 min at 37°C, to allow conversion of BAAA into a fluorescent product (BAA). Fluorescence was measured by flow cytometry (FACS Calibur) using adjusted FSC and SSC voltages to center the nucleated and viable cell population. DEAB treated vs. non treated cells were used to identify Aldefluor positive cells. Median fluorecence intensity (MFI) was derived from each sample and the baseline fluorescence, used to identify the Aldefluor positive and negative populations, was calculated by the difference between the MFI of sample containing BAAA and the MFI of the same sample containing also DEAB. Sorting of the populations of interest was performed on ARIA II cell sorter (Beckton Dickinson) to near purity (85%). CD44/CD24 flow cytometric analysis was performed with mouse anti-human PE conjugated anti-CD44 and FITC conjugated anti-CD24 antibodies (BD).

**Antibodies for Western blot, Far Western, Immunoprecipitations, and Immunohistochemistry.**

The following antibodies were used: rabbit and goat polyclonal anti-Notch1 (C-20: sc-6014, and S-20: sc-23304, SantaCruz), rabbit polyclonal anti-N1-ICD Val1744 (#2421, Cell Signalling), rabbit polyclonal anti-N4-ICD Val1432 (SAB4502023, Sigma), rabbit polyclonal anti-Notch4 (H-225, sc-5594), rabbit polyclonal anti-Pin1 (Rustighi et al., 2009) and mouse monoclonal anti-Pin1 (G-8: sc-46660, Santa Cruz), rabbit polyclonal anti-HES-1 (#AB5702, Millipore), rabbit polyclonal anti-Slug (#9585S, Cell Signaling), mouse monoclonal anti-Vimentin (ab8069, Abcam), mouse monoclonal anti-E-cadherin (610182, BD), rabbit monoclonal anti-cleaved Caspase-3 (5A1E, Cell Signaling), rabbit polyclonal anti-Mcl-1 (S-19: sc-819, Santa Cruz), for immunoprecipitation and Western blot of endogenous Fbxw7α mouse monoclonal (Abnova MO2, 3D1) and rabbit polyclonal (Abcam ab12292) antibodies were used, respectively. For tagged proteins rabbit polyclonal and mouse monoclonal (9B11) anti-myc (#2272 and #2276 respectively, Cell Signaling), mouse monoclonal anti-Flag clone M2 (F3165, Sigma), anti-GFP rabbit polyclonal

serum was raised against GST-GFP fusion protein expressed in bacteria, affinity purified and used 1:1000, mouse monoclonal (12CA5) and rabbit polyclonal (Y-11, sc-805 Santa Cruz) anti-HA. For immunohistochemical stainings anti-cleaved Notch1 (Abcam 8925) anti-Notch1 (Santa-Cruz C-20), anti-Notch4 (Santa Cruz H-225), anti-Fbxw7α (Abnova MO2, 3D1), and anti-Pin1 home made anti-rabbit (Rustighi et al. 2009) were used.

**Western blot, *in vitro* binding, immunoprecipitation.** *In vitro* binding assays, immuno- and co-immunoprecipitations and Western blot analyses were performed by standard procedures, as described (Rustighi et al., 2009). Briefly, for GST and GST-Pin1 pull-down analysis using cells were lysed in GST pull-down buffer (150 mM NaCl, 50 mM Tris/HCl pH 7.5, 10% glycerol, 0.1% Nonidet P-40, Sigma), supplemented with inhibitors of phosphatase (1mM sodium orthovanadate, 5mM NaF, Sigma) and protease (phenylmethylsulfonylfluoride (PMSF) 1mM and chymostatin, leupeptin, antipain, pepstatin 10 μg $ml^{-1}$ each, Sigma). For Notch1 immunoprecipitations cells were treated in all cases with proteasome inhibitor lactacystin for 8 hrs and collected in GST pull-down buffer as above. Cell lysates were cleared with proteinA Sepharose by rocking for 30 min, then Protein A/G sepharose (GE Healthcare) cross-linked antibodies, precleared with 10 mg/ml BSA (Sigma), were added. Binding reactions were left for a minimum of 4 hours to over night rocking at 4°C. Then beads were washed and bound proteins were loaded and separated in SDS-PAGE, followed by Western blotting on Nitrocellulose membranes (Scleicher & Schuell). For β-mercaptoethanol stripping of the primary antibody membranes were shortly boiled in 0.1 mM β-mercaptoethanol, 1% SDS, 50 mM Tris/HCl pH 6.9, then washed by shaking at RT with PBS, followed by blocking in Blotto-tween (PBS, 0.2% Tween-20, not fat dry milk 5%) or with TBST (Tris/HCl 25 mM pH7.5) plus 5% BSA (Sigma) depending on the antibody.

Purified GST-Pin1 protein for Far Western analysis was obtained by immobilization, after production in bacteria, on glutathione sepharose 4B beads (GEhealthcare) followed by elutions using reduced GSH as a competitor in Tris/HCl pH8 100mM and NaCl 100mM. The eluted protein was subsequently purified by dialysis.

For Far Western Blot analysis proteins were immunoprecipitated with the indicated antibody, resolved by SDS Page and blotted onto nitrocellulose membrane. Blocking was performed for 1h at 4°C in PBS plus not fat dry milk 10%. Blots were then incubated with 1μg/ml GST-Pin1 protein in blocking buffer for 1h. Membranes were washed 4 times in PBS, 0.2% Tween-20. Subsequently recognition by standard Western blot was performed.

**Immunohistochemical analyses.**

Mammary tissue from *Pin1*$^{+/+}$ and *Pin1*$^{-/-}$ mice was collected, formalin fixed and embedded in paraffin. 3 µm sections were stained with anti-Notch1 (Santa Cruz C-20, 1:50), anti-Notch4 (Santa Cruz H-225, 1:100), anti-Fbxw7α (Abnova MO2, 3D1, 1:250), and anti-Pin1 (home made anti-rabbit, 1:200, Rustighi et al. 2009) antibodies. For immunohistochemical analysis of human breast cancers, anti-cleaved Notch1 (Abcam 8925, 1:200), anti-Pin1 (Santa Cruz mouse monoclonal G-8: sc-46660) and the abovementioned anti-Fbxw7α antibody were used. Briefly, stainings were performed according to standard procedures for paraffin embedded tissue. Slides were deparaffinized, rehydrated and antigen retrieved in a calibrated steam pressure cooker with citrate buffer (pH 6.0). Endogenous peroxidase was blocked with peroxidase block from an EnVision™ Kit (DakoCytomation, Glostrup, Denmark) for 15 minutes. The slides were blocked in 5% nonfat dry milk or PBS + 5% BSA for 20 minutes to 1 hour at room temperature to minimize nonspecific binding due to hydrophobic interaction. The slides were then incubated in blocking buffer without any antibody (negative control) or with the indicated primary antibodies for 1 hour at 37°C or overnight at 4°C. After washing, slides were incubated with secondary universal antibody (Vectastain) for 45 minutes at room temperature. Colorimetric detection was completed with diaminobenzidine and hydrogen peroxidase for 6 minutes and counterstained with hematoxylin (Sigma-Aldrich, St. Louis, MO, USA). The immunostaining was scored semiquantitatively. In the evaluation of activated/cleaved Notch1 and Fbxw7α only nuclear staining was considered. The scores for IHC were: 0, no staining; 1, few nuclear staining of tumor cells (<5% of positive tumor cells); 2, 5-10% of positive tumor cells; 3, >10% positive tumor cells. For pairwise comparisons, the scores were collapsed to low (score, 1–2) versus high (score, 3) expression, excluding not interpretable samples.

**Data acquisition, Image processing, Equipment and settings**

Western blot films were scanned with an Epson Stylus DX7450 scanner at a grey scale 600 d.p.i. resolution and saved as Tiff files. Single panels were cropped to obtain individual layers by Photpshop software. Densitometric values of protein levels in Western blot analyses were obtained by Image J software. Where necessary, for the sake of clarity, parts of the same or different gels with equal molecular weights were cropped and juxtaposed, demarcated with borders and indicated in the figure legend. Images of mouse and human immunohistochemical analyses were obtained using Leica DM4000B Microscope with DFC420C photocamera. Pictures of Lymph node metastases were taken with an Olympus Super bright Zoom Lens F1.8, C-4040200M, 4.1 Megapixel, 7.5 digital zoom. Images of haematoxylin and eosin stained sections of pulmonary metastases were obtained with an Olympus BX40 microscope and Leica DFC295 CH-9435 camera and were used for Computer-aided assessment of percentage of lung tissue area occupied by

metastases. Microscope image files were obtained through Leica Application Suite LAS 4.1 software.

**Chromatin Immunoprecipitation analysis.**

Binding of N4-ICD to the genomic regions of the HES-1 and Pin1 promoters was performed as described (Rustighi et al., 2009). Binding of N4-ICD was quantified by calculating the percentage of input chromatin by real-time PCR. Cells were cross-linked with 1% formaldehyde for 15 min, neutralized with 125 mM glycine pH 2.5 and washed in PBS. Nuclei were prepared by hypotonic lysis (5mM Pipes pH 6.8, 85 mM KCl, 0.5% Nonidet-P40) and centrifugation, and resuspended in RIPA-100 buffer (20 mM Tris/HCl, pH 7.5, 100 mM NaCl, 1mM EDTA, 0.5% Nonidet P-40, 0.5% deoxycholate, 0.1% SDS) with protease inhibitor cocktail (Sigma), 1 mM PMSF and phosphatase inhibitors (NaF 5 mM, $Na_3VO_4$ 1 mM). Chromatin was sonicated by Bioruptor (Diagenode) to 500-1000 bp average fragment size and cleared by centrifugation. IP was performed overnight at 4°C with either 2 μg of a rabbit polyclonal anti-N4-ICD (Val1432) or an unrelated rabbit polyclonal antibody (anti-HA). DNA-protein complexes were recovered by protein A/G PLUS-Agarose (GE Heathcare) and washed sequentially with RIPA-100 and RIPA-250 buffer and LiCl solution (10 mM Tris/HCl, pH 8, 250 mM NaCl, 1mM EDTA, 0.5% Nonidet P-40, 0.5% deoxycholate), then resuspended in T.E., digested with 2U of DNAse-free RNAse (Calbiochem) for 30 min at 37°C, and incubated overnight at 68°C with 300 mg/ml proeinase K (NEB) in 0.5% SDS, 100 mM NaCl to digest proteins and reverse cross-links. After purification by phenol-chloroform separation and ethanol precipitation, DNA was resuspended in $H_2O$ and 1/10 volume was used for quantification. 1/10 of input chromatin was amplified as a standard for each experiment. PCR products were resolved on 2% agarose gels, visualized by EtBr staining, and quantified with Kodak Digital Science 1d 2.0.2 software. PCR settings are available upon request.

**Breast cancer data collection and processing**

We collected 21 datasets comprising microarray data of breast cancer samples and annotations on patients' clinical outcome. All data were measured on Affymetrix arrays and have been downloaded from Gene Expression Omnibus (http://www.ncbi.nlm.nih.gov/geo) and ArrayExpress (http://www.ebi.ac.uk/arrayexpress/). The complete list of datasets is provided in Table S1. Prior to analysis, we reorganized the datasets eliminating duplicate samples and samples without outcome information and renamed any original study after the medical center where patients were recruited. Briefly, the original studies have been modified as follows:

-Stockholm has been used as it is and re-named as KI_Stockholm (Karolinska Institutet Stockholm); -EMC-286 and EMC-58 have been merged into EMC-344 (Erasmus Medical

Center); -MSK has been used as is and re-named as MSKCC (Memorial Sloan-Kettering Cancer Center); -Uppsala-Miller, Ivshina-Miller, and Loi datasets (GSE3494, GSE4922, and GSE6532) included samples derived from Uppsala University Hospital, John Radcliffe Hospital in Oxford, and Guys Hospital in London. Moreover, a comparison of the hybridization dates on the raw files and of the patients' clinical information revealed that, although deposited twice, GSE3494 and GSE4922 were identical. As such, the 3 series have been split into KI_Uppsala, comprising all 253 unique patients of the Uppsala University Hospital, OXF composed of the 178 samples collected at the John Radcliffe Hospital in Oxford, and GUY composed of the 87 samples from the Guys Hospital in London;

-Sotiriou is entirely included in GSE6532;

-Desmedt has been used as is and renamed as TRANSBIG (after the consortium of cancer centers where samples have been collected);

-Schmidt has been used as is and re-named as Mainz (Johannes Gutenberg University in Mainz);

-Veridex has been used as is and re-named as Veridex_MultiCenter (after the three European and one US institutions where samples have been collected);

-Tamoxifen has been added to GUY;

-Chin and Zhou have been merged into UCSF (University of California, San Francisco) after removing samples deposited in both GEO (GSE7378) and ArrayExpress (E-TABM-158);

-TOP TRIAL has been re-named as IJB_TOP (Institut Jules Bordet /Trial of Principle) after removal of 13 samples lacking of outcome information;

-GSE19615 has been used as is and re-named as US_NCI (US National Cancer Institute);

-IPC has been re-named as CRCM (Centre de cancérologie de Marseille) after removal of 14 samples lacking of outcome information;

-KFSYSCC has been used as is and re-named as KOOF (Koo Foundation SYS Cancer Center);

-GSE31519 has been used as is and re-named as Goethe (Goethe-University, Frankfurt) after removal of 2 samples lacking of outcome information;

-Hatzis included samples derived from 4 cohorts, i.e., I-SPY-1 (Investigation of Serial Studies to Predict Your Therapeutic Response With Imaging and Molecular Analysis), LBJ_INEN_GEICAM (Lyndon B. Johnson Hospital, Instituto Nacional de Enfermedades Neoplásicas, and Grupo Español de Investigación en Cáncer de Mama), USO-02103 (US Oncology), and MDACC (M. D. Anderson Cancer Center, Houston). The orginal data has been split into I-SPY-1 comprising 83 samples, LBJ_INEN_GEICAM comprising 58 samples, MDACC comprising 313 samples, and USO-02103 comprising 54 samples.

This re-organization resulted in a meta-dataset comprising 3254 unique samples from 19 independent cohorts (Table S1). According to Cordenonsi et al. (2011), we standardized clinical

information among the various datasets redefining the outcome descriptions based on the clinical annotations of each individual study. Specifically, we defined survival as death because of cancer and includes overall survival, disease free survival, and disease specific survival. Raw expression data (i.e., CEL files) obtained from different platforms have been integrated using an approach inspired by geometry and probe content of HG-U133 Affymetrix arrays (Fallarino et al., 2010). Briefly, probes with the same oligonucleotide sequence, but located at different coordinates on different type of arrays, may be arranged in a virtual platform grid. As for any other microarray geometry, this virtual grid may be used as a reference to create a virtual Chip Definition File (virtual-CDF), containing the probes shared among the various HG-U133 platforms and their coordinates on the virtual platform, and a virtual-CEL files containing the fluorescence intensities of the original CEL files properly re-mapped on the virtual grid. Once defined the virtual platform through the creation of the virtual-CDF and transformed the CEL files into virtual-CELs, raw data, originally obtained from different HG-U133 arrays, are homogeneous in terms of platform and can be preprocessed and normalized adopting standard approaches, as RMA (Irizarry et al., 2003). Specifically, expression values were generated from intensity signals using the virtual-CDF, obtained merging HG-U133A, HG-U133AAofAV2, and HG-U133 Plus2 original CDFs, and the transformed virtual-CEL files. Intensity values for a total of 21981 meta-probe sets have been background adjusted, normalized using quantile normalization, and gene expression levels calculated using median polish summarization (RMA). The entire procedure has been implemented as an R script. To identify two groups of tumor samples with either high or low levels of the Notch-dependent Direct Target (NDT) gene signature we used the classifier described in Adorno et al (2009). Briefly, we defined a classification rule based on summarizing the standardized expression levels of each gene in NDT signature into a combined score with zero mean. Tumors were then classified as NDT signature Low if the combined score was negative and as NDT signature High if the combined score was positive. Similarly, we defined tumors as expressing high or low levels of Fbxw7 and Pin1 mRNA if the standardized expression signal of Fbxw7 and Pin1 probes was positive or negative, respectively. This classification was applied to log2 expression values obtained using RMA on the meta-dataset described above.

**Survival analysis**

To evaluate the prognostic value of the NDT signatures, we estimated, using the Kaplan-Meier method (Kalbfleisch and Prentice), the probabilities that patients would remain free of death (survival). To confirm these findings, the Kaplan-Meier curves were compared using the log-rank or Mantel-Haenszel test (Harrington and Fleming). P-values were calculated according to the standard normal asymptotic distribution. When comparing “NDT signature High” and “NDT

signature Low” groups, the group with low NDT levels displayed a significantly higher probability (at a significance level α=5x10-2) of a reduced survival (Figure S8B). Survival analysis and Kaplan-Meier plots were obtained using R *survcomp* package. Kaplan-Meyer curves have been compared using the log-rank test of the *surv_test* \function (*coin* R package).

## Supporting References


Adorno, M, Cordenonsi, M, Montagner, M, Dupont, S, Wong, C, Hann, B, Solari, A, Bobisse, S, Rondina, M B, Guzzardo, V, et al (2009) A Mutant-p53/Smad complex opposes p63 to empower TGFbeta-induced metastasis. Cell 137: 87-98

Amsen, D, Antov, A, Jankovic, D, Sher, A, Radtke, F, Souabni, A, Busslinger, M, McCright, B, Gridley, T, and Flavell, R A (2007) Direct regulation of Gata3 expression determines the T helper differentiation potential of Notch. Immunity 27: 89-99

Butler, J S, Queally, J M, Devitt, B M, Murray, D W, Doran, P P, and O'Byrne, J M (2010) Silencing Dkk1 expression rescues dexamethasone-induced suppression of primary human osteoblast differentiation. BMC Musculoskelet Disord 11: 210

Casas, E, Kim, J, Bendesky, A, Ohno-Machado, L, Wolfe, C J, and Yang, J (2011) Snail2 is an essential mediator of Twist1-induced epithelial mesenchymal transition and metastasis. Cancer Res 71: 245-254

Chadwick, N, Zeef, L, Portillo, V, Fennessy, C, Warrander, F, Hoyle, S, and Buckle, A M (2009) Identification of novel Notch target genes in T cell leukaemia. Mol Cancer 8, 35

Chen, Y, Fischer, W H, and Gill, G N (1997) Regulation of the ERBB-2 promoter by RBPJkappa and NOTCH. J Biol Chem 272: 14110-14114

Cheung, C H, Chen, H H, Kuo, C C, Chang, C Y, Coumar, M S, Hsieh, H P, and Chang, J Y (2009) Survivin counteracts the therapeutic effect of microtubule de-stabilizers by stabilizing tubulin polymers. Mol Cancer 8: 43

Cordenonsi, M, Zanconato, F, Azzolin, L, Forcato, M, Rosato, A, Frasson, C, Inui, M, Montagner, M, Parenti, A R, Poletti, A, et al (2011) The Hippo transducer TAZ confers cancer stem cell-related traits on breast cancer cells. Cell 147: 759-772

Deftos, M L, Huang, E, Ojala, E W, Forbush, K A, and Bevan, M J (2000) Notch1 signaling promotes the maturation of CD4 and CD8 SP thymocytes. Immunity 13: 73-84

Desmedt, C, Di Leo, A, de Azambuja, E, Larsimont, D, Haibe-Kains, B, Selleslags, J, Delaloge, S, Duhem, C, Kains, J P, Carly, B, et al (2011) Multifactorial approach to predicting resistance to anthracyclines. J Clin Oncol 29: 1578-1586

Desmedt, C, Piette, F, Loi, S, Wang, Y, Lallemand, F, Haibe-Kains, B, Viale, G, Delorenzi, M, Zhang, Y, d'Assignies, M S, et al (2007) Strong time dependence of the 76-gene prognostic signature for node-negative breast cancer patients in the TRANSBIG multicenter independent validation series. Clin Cancer Res 13: 3207-3214

Fallarino, F, Volpi, C, Fazio, F, Notartomaso, S, Vacca, C, Busceti, C, Bicciato, S, Battaglia, G, Bruno, V, Puccetti, P, et al (2010) Metabotropic glutamate receptor-4 modulates adaptive immunity and restrains neuroinflammation. Nat Med 16: 897-902

Girardini, JE, Napoli, M, Piazza, S, Rustighi, A, Marotta, C, Radaelli, E, Capaci, V, Jordan, L, Quinlan, P, Thompson, A, et al (2011) A Pin1/mutant p53 axis promotes aggressiveness in breast cancer. Cancer Cell 20: 79-91

Guichard, C, Pedruzzi, E, Fay, M, Marie, J C, Braut-Boucher, F, Daniel, F, Grodet, A, Gougerot-Pocidalo, M A, Chastre, E, Kotelevets, L, et al (2006) Dihydroxyphenylethanol induces apoptosis by activating serine/threonine protein phosphatase PP2A and promotes the endoplasmic reticulum stress response in human colon carcinoma cells. Carcinogenesis 27: 1812-1827

Hamidi, H, Gustafason, D, Pellegrini, M, and Gasson, J (2011) Identification of novel targets of CSL-dependent Notch signaling in hematopoiesis. PLoS One 6: e20022

Hatzis, C, Pusztai, L, Valero, V, Booser, D J, Esserman, L, Lluch, A, Vidaurre, T, Holmes, F, Souchon, E, Wang, H, et al (2011) A genomic predictor of response and survival following taxane-anthracycline chemotherapy for invasive breast cancer. JAMA 305: 1873-1881

Irizarry, R A, Hobbs, B, Collin, F, Beazer-Barclay, Y D, Antonellis, K J, Scherf, U, and Speed, T P (2003) Exploration, normalization, and summaries of high density oligonucleotide array probe level data. Biostatistics 4: 249-264

Ivshina, A V, George, J, Senko, O, Mow, B, Putti, T C, Smeds, J, Lindahl, T, Pawitan, Y, Hall, P, Nordgren, H, et al (2006) Genetic reclassification of histologic grade delineates new clinical subtypes of breast cancer. Cancer Res 66: 10292-10301

Iwamoto, T, Bianchini, G, Booser, D, Qi, Y, Coutant, C, Shiang, C Y, Santarpia, L, Matsuoka, J, Hortobagyi, G N, Symmans, W F, et al (2011) Gene pathways associated with prognosis and chemotherapy sensitivity in molecular subtypes of breast cancer. J Natl Cancer Inst 103: 264-272

Izon, D J, Aster, J C, He, Y, Weng, A, Karnell, F G, Patriub, V, Xu, L, Bakkour, S, Rodriguez, C, Allman, D, and Pear, W S (2002) Deltex1 redirects lymphoid progenitors to the B cell lineage by antagonizing Notch1. Immunity 16: 231-243

Jeffries, S, and Capobianco, A J (2000) Neoplastic transformation by Notch requires nuclear localization. Mol Cell Biol 20: 3928-3941

Juul, N, Szallasi, Z, Eklund, A C, Li, Q, Burrell, R A, Gerlinger, M, Valero, V, Andreopoulou, E, Esteva, F J, Symmans, W F, et al (2010) Assessment of an RNA interference screen-derived mitotic and ceramide pathway metagene as a predictor of response to neoadjuvant paclitaxel for primary triple-negative breast cancer: a retrospective analysis of five clinical trials. Lancet Oncol 11: 358-365

Kao, K J, Chang, K M, Hsu, H C, and Huang, A T (2011) Correlation of microarray-based breast cancer molecular subtypes and clinical outcomes: implications for treatment optimization. BMC Cancer 11: 143

Karn, T, Pusztai, L, Holtrich, U, Iwamoto, T, Shiang, C Y, Schmidt, M, Muller, V, Solbach, C, Gaetje, R, Hanker, L, et al (2011) Homogeneous datasets of triple negative breast cancers enable the identification of novel prognostic and predictive signatures. PLoS One 6: e28403

Karn, T, Pusztai, L, Ruckhaberle, E, Liedtke, C, Muller, V, Schmidt, M, Metzler, D, Wang, J, Coombes, K R, Gatje, R, et al (2012) Melanoma antigen family A identified by the bimodality index defines a subset of triple negative breast cancers as candidates for immune response augmentation. Eur J Cancer 48: 12-23

Lamar, E, Deblandre, G, Wettstein, D, Gawantka, V, Pollet, N, Niehrs, C, and Kintner, C (2001) Nrarp is a novel intracellular component of the Notch signaling pathway. Genes Dev 15: 1885-1899

Lee, C W, Raskett, C M, Prudovsky, I, and Altieri, D C (2008) Molecular dependence of estrogen receptor-negative breast cancer on a notch-survivin signaling axis. Cancer Res 68: 5273-5281

Lee, T H, Chen, C H, Suizu, F, Huang, P, Schiene-Fischer, C, Daum, S, Zhang, Y J, Goate, A, Chen, R H, Zhou, X Z, and Lu, K P (2011) Death-associated protein kinase 1 phosphorylates Pin1 and inhibits its prolyl isomerase activity and cellular function. Mol Cell 42: 147-159

Leong, K G, Niessen, K, Kulic, I, Raouf, A, Eaves, C, Pollet, I, and Karsan, A (2007) Jagged1-mediated Notch activation induces epithelial-to-mesenchymal transition through Slug-induced repression of E-cadherin. J Exp Med 204: 2935-2948

Li, H, Collado, M, Villasante, A, Strati, K, Ortega, S, Canamero, M, Blasco, M A, and Serrano, M (2009) The Ink4/Arf locus is a barrier for iPS cell reprogramming. Nature 460: 1136-1139

Li, Y, Zou, L, Li, Q, Haibe-Kains, B, Tian, R, Desmedt, C, Sotiriou, C, Szallasi, Z, Iglehart, J D, Richardson, A L, and Wang, Z C (2010) Amplification of LAPTM4B and YWHAZ contributes to chemotherapy resistance and recurrence of breast cancer. Nat Med 16: 214-218

Liu, L, Andrews, L G, and Tollefsbol, T O (2006) Loss of the human polycomb group protein BMI1 promotes cancer-specific cell death. Oncogene 25: 4370-4375

Loi, S, Haibe-Kains, B, Desmedt, C, Lallemand, F, Tutt, A M, Gillet, C, Ellis, P, Harris, A, Bergh, J, Foekens, J A, et al (2007) Definition of clinically distinct molecular subtypes in estrogen receptor-positive breast carcinomas through genomic grade. J Clin Oncol 25: 1239-1246

Loi, S, Haibe-Kains, B, Majjaj, S, Lallemand, F, Durbecq, V, Larsimont, D, Gonzalez-Angulo, A M, Pusztai, L, Symmans, W F, Bardelli, A, et al (2010) PIK3CA mutations

associated with gene signature of low mTORC1 signaling and better outcomes in estrogen receptor-positive breast cancer. Proc Natl Acad Sci U S A 107: 10208-10213

MacKenzie, F, Duriez, P, Wong, F, Noseda, M, and Karsan, A (2004) Notch4 inhibits endothelial apoptosis via RBP-Jkappa-dependent and -independent pathways. J Biol Chem 279: 11657-11663

Miller, L D, Smeds, J, George, J, Vega, V B, Vergara, L, Ploner, A, Pawitan, Y, Hall, P, Klaar, S, Liu, E T, and Bergh, J (2005) An expression signature for p53 status in human breast cancer predicts mutation status, transcriptional effects, and patient survival. Proc Natl Acad Sci U S A 102: 13550-13555

Minn, A J, Gupta, G P, Padua, D, Bos, P, Nguyen, D X, Nuyten, D, Kreike, B, Zhang, Y, Wang, Y, Ishwaran, H, et al (2007) Lung metastasis genes couple breast tumor size and metastatic spread. Proc Natl Acad Sci U S A 104: 6740-6745

Minn, A J, Gupta, G P, Siegel, P M, Bos, P D, Shu, W, Giri, D D, Viale, A, Olshen, A B, Gerald, W L, and Massague, J (2005) Genes that mediate breast cancer metastasis to lung. Nature 436: 518-524

Oswald, F, Liptay, S, Adler, G, and Schmid, R M (1998) NF-kappaB2 is a putative target gene of activated Notch-1 via RBP-Jkappa. Mol Cell Biol 18: 2077-2088

Palomero, T, Lim, W K, Odom, D T, Sulis, M L, Real, P J, Margolin, A, Barnes, K C, O'Neil, J, Neuberg, D, Weng, A P, et al (2006) NOTCH1 directly regulates c-MYC and activates a feed-forward-loop transcriptional network promoting leukemic cell growth. Proc Natl Acad Sci U S A 103: 18261-18266

Pawitan, Y, Bjohle, J, Amler, L, Borg, A L, Egyhazi, S, Hall, P, Han, X, Holmberg, L, Huang, F, Klaar, S, et al (2005) Gene expression profiling spares early breast cancer patients from adjuvant therapy: derived and validated in two population-based cohorts. Breast Cancer Res 7: R953-964

Popovici, V, Chen, W, Gallas, B G, Hatzis, C, Shi, W, Samuelson, F W, Nikolsky, Y, Tsyganova, M, Ishkin, A, Nikolskaya, T, et al (2010) Effect of training-sample size and classification difficulty on the accuracy of genomic predictors. Breast Cancer Res 12: R5

Rangarajan, A, Talora, C, Okuyama, R, Nicolas, M, Mammucari, C, Oh, H, Aster, J C, Krishna, S, Metzger, D, Chambon, P, et al (2001) Notch signaling is a direct determinant of keratinocyte growth arrest and entry into differentiation. EMBO J 20: 3427-3436

Rody, A, Karn, T, Liedtke, C, Pusztai, L, Ruckhaeberle, E, Hanker, L, Gaetje, R, Solbach, C, Ahr, A, Metzler, D, et al (2011) A clinically relevant gene signature in triple negative and basal-like breast cancer. Breast Cancer Res 13: R97

Ronchini, C, and Capobianco, A J (2001) Induction of cyclin D1 transcription and CDK2 activity by Notch(ic): implication for cell cycle disruption in transformation by Notch(ic). Mol Cell Biol 21: 5925-5934

Rustighi, A, Tiberi, L, Soldano, A, Napoli, M, Nuciforo, P, Rosato, A, Kaplan, F, Capobianco, A, Pece, S, Di Fiore, P P, and Del Sal, G (2009) The prolyl-isomerase Pin1 is a Notch1 target that enhances Notch1 activation in cancer. Nat Cell Biol 11: 133-142

Sabatier, R, Finetti, P, Adelaide, J, Guille, A, Borg, J P, Chaffanet, M, Lane, L, Birnbaum, D, and Bertucci, F (2011a) Down-regulation of ECRG4, a candidate tumor suppressor gene, in human breast cancer. PLoS One 6: e27656

Sabatier, R, Finetti, P, Cervera, N, Lambaudie, E, Esterni, B, Mamessier, E, Tallet, A, Chabannon, C, Extra, J M, Jacquemier, J, et al (2011b) A gene expression signature identifies two prognostic subgroups of basal breast cancer. Breast Cancer Res Treat 126: 407-420

Sahlgren, C, Gustafsson, M V, Jin, S, Poellinger, L, and Lendahl, U (2008) Notch signaling mediates hypoxia-induced tumor cell migration and invasion. Proc Natl Acad Sci U S A 105: 6392-6397

Sarmento, L M, Huang, H, Limon, A, Gordon, W, Fernandes, J, Tavares, M J, Miele, L, Cardoso, A A, Classon, M, and Carlesso, N (2005) Notch1 modulates timing of G1-S progression by inducing SKP2 transcription and p27 Kip1 degradation. J Exp Med 202: 157-168

Schmidt, M, Bohm, D, von Torne, C, Steiner, E, Puhl, A, Pilch, H, Lehr, H A, Hengstler, J G, Kolbl, H, and Gehrmann, M (2008) The humoral immune system has a key prognostic impact in node-negative breast cancer. Cancer Res 68: 5405-5413

Shi, L, Campbell, G, Jones, W D, Campagne, F, Wen, Z, Walker, S J, Su, Z, Chu, T M, Goodsaid, F M, Pusztai, L, et al (2010) The MicroArray Quality Control (MAQC)-II study of common practices for the development and validation of microarray-based predictive models. Nat Biotechnol 28: 827-838

Sotiriou, C, and Desmedt, C (2006) Gene expression profiling in breast cancer. Ann Oncol 17 Suppl 10: x259-262

Stingl, J, Eirew, P, Ricketson, I, Shackleton, M, Vaillant, F, Choi, D, Li, H I, and Eaves, C J (2006) Purification and unique properties of mammary epithelial stem cells. Nature 439: 993-997

Wang, Y, Klijn, J G, Zhang, Y, Sieuwerts, A M, Look, M P, Yang, F, Talantov, D, Timmermans, M, Meijer-van Gelder, M E, Yu, J, et al (2005) Gene-expression profiles to predict distant metastasis of lymph-node-negative primary breast cancer. Lancet 365: 671-679

Welcker, M, Orian, A, Jin, J, Grim, J E, Harper, J W, Eisenman, R N, and Clurman, B E (2004) The Fbw7 tumor suppressor regulates glycogen synthase kinase 3 phosphorylation-dependent c-Myc protein degradation. Proc Natl Acad Sci U S A 101: 9085-9090

Weng, A P, Millholland, J M, Yashiro-Ohtani, Y, Arcangeli, M L, Lau, A, Wai, C, Del Bianco, C, Rodriguez, C G, Sai, H, Tobias, J, et al (2006) c-Myc is an important direct target of Notch1 in T-cell acute lymphoblastic leukemia/lymphoma. Genes Dev 20: 2096-2109

Yang, T, Martin, M L, Nielsen, J S, Milne, K, Wall, E M, Lin, W, Watson, P H, and Nelson, B H (2009) Mammary tumors with diverse immunological phenotypes show differing sensitivity to adoptively transferred CD8+ T cells lacking the Cbl-b gene. Cancer Immunol Immunother 58: 1865-1875

Yau, C, and Benz, C C (2008) Genes responsive to both oxidant stress and loss of estrogen receptor function identify a poor prognosis group of estrogen receptor positive primary breast cancers. Breast Cancer Res 10: R61

Zhang, Y, Sieuwerts, A M, McGreevy, M, Casey, G, Cufer, T, Paradiso, A, Harbeck, N, Span, P N, Hicks, D G, Crowe, J, et al (2009) The 76-gene signature defines high-risk patients that benefit from adjuvant tamoxifen therapy. Breast Cancer Res Treat 116: 303-309

Zhou, Y, Yau, C, Gray, J W, Chew, K, Dairkee, S H, Moore, D H, Eppenberger, U, Eppenberger-Castori, S, and Benz, C C (2007) Enhanced NF kappa B and AP-1 transcriptional activity associated with antiestrogen resistant breast cancer. BMC Cancer 7: 59
